# Multicomponent synthesis of chromophores – The one-pot approach to functional π-systems

**DOI:** 10.3389/fchem.2023.1124209

**Published:** 2023-03-17

**Authors:** Larissa Brandner, Thomas J. J. Müller

**Affiliations:** Institut für Organische Chemie und Makromolekulare Chemie, Heinrich-Heine-Universität Düsseldorf, Düsseldorf, Germany

**Keywords:** absorption, chromophores, diversity-oriented synthesis, donor-acceptor systems, fluorescence, multicomponent reactions, one-pot synthesis

## Abstract

Multicomponent reactions, conducted in a domino, sequential or consecutive fashion, have not only considerably enhanced synthetic efficiency as one-pot methodology, but they have also become an enabling tool for interdisciplinary research. The highly diversity-oriented nature of the synthetic concept allows accessing huge structural and functional space. Already some decades ago this has been recognized for life sciences, in particular, lead finding and exploration in pharma and agricultural chemistry. The quest for novel functional materials has also opened the field for diversity-oriented syntheses of functional π-systems, i.e. dyes for photonic and electronic applications based on their electronic properties. This review summarizes recent developments in MCR syntheses of functional chromophores highlighting syntheses following either the framework forming scaffold approach by establishing connectivity between chromophores or the chromogenic chromophore approach by *de novo* formation of chromophore of interest. Both approaches warrant rapid access to molecular functional π-systems, i.e. chromophores, fluorophores, and electrophores for various applications.

## Graphical Abstract

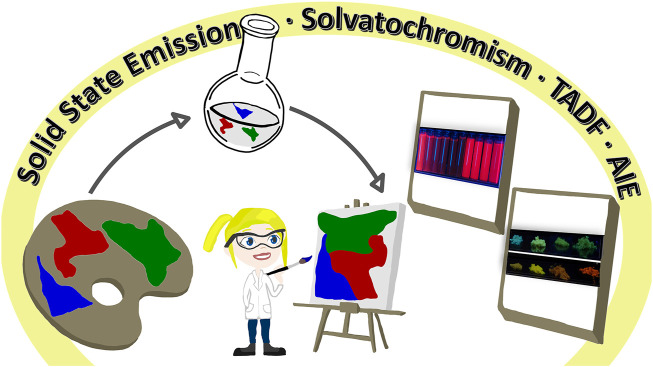

## 1 Introduction

Chromophores have increasingly become functional organic materials (Müller, 2007). As a consequence of their photophysical properties, such as fluorescence and aggregation-induced emission (AIE) (Hong et al., 2009; Hong et al., 2011; Hu et al., 2014; Mei et al., 2015; Mazumdar et al., 2016), and their electrochemical characteristics they find broad application in organic light-emitting diodes (OLEDs) (Müllen and Scherf, 2006; Park et al., 2011; Kalyani and Dhoble, 2012; Li, 2015), dye-sensitized solar cells (DSSCs) (Grätzel, 2001; Mishra et al., 2009), organic photovoltaics (OPVs) (Yam, 2010; Su et al., 2012; Ameri et al., 2013), organic field effect transistors (OFETs) (Kymissis, 2008; Torsi et al., 2013) and in bio- or environmental analytics (Chen et al., 1998; Nilsson et al., 2002; Wagenknecht, 2008; Kim and Park, 2009; Cairo et al., 2010), as well as in medicinal and pharmaceutical applications (Dua et al., 2011; Arora et al., 2012; Saini et al., 2013; Kumar and Kumar Jain, 2016; Taylor et al., 2016; Asif, 2017; Taek Han et al., 2017; Petronijevic et al., 2018; Soleymani and Chegeni, 2019). The ongoing quest for novel, efficient syntheses of new functional chromophores with well-defined features and thoroughly fine-tuned properties remains a challenge. Research on diversity-oriented syntheses concepts for functional chromophores has become highly relevant over the past one and a half decades (Müller and D'Souza, 2008; Briehn and Bäuerle, 2002; Zhu et al., 2014; D'Souza and Müller, 2007). In particular, multicomponent reactions (MCRs) (Weber et al., 1999; Bienaymé et al., 2000; Dömling and Ugi, 2000; Zhu and Bienaymé, 2006; Müller, 2016; Riva et al., 2016) have been established as a promising tool for the synthesis of functional π -electron systems (Levi and Müller, 2016a; Levi and Müller, 2016b; de Moliner et al., 2017; Merkt and Müller, 2018) in recent years.

By definition MCRs proceed in a one-pot fashion, where more than two starting components react to form a product containing most of the deployed atoms. In *sensu stricto*, the process is not only conducted in a single reaction vessel, but also without changing the solvent, filtration or any other workup operations. Indeed, MCR represent a reactivity-based concept (Müller, 2014), where reactivities of functional groups can be employed in three different ways. In a domino MCRs, all compounds are present in the vessel from the beginning of the process. In a sequential MCR, reactants are added in a well-defined order while the reaction conditions maintained constant. Finally, MCRs taking advantage of defined order of transformation of the reactants, yet, with variably adjusting the reaction conditions from step to step are considered as consecutive processes. All MCR have in common high levels of functional and structural diversity with a minimum of purification operations at the end of the one-pot processes.

**SCHEME 1:**
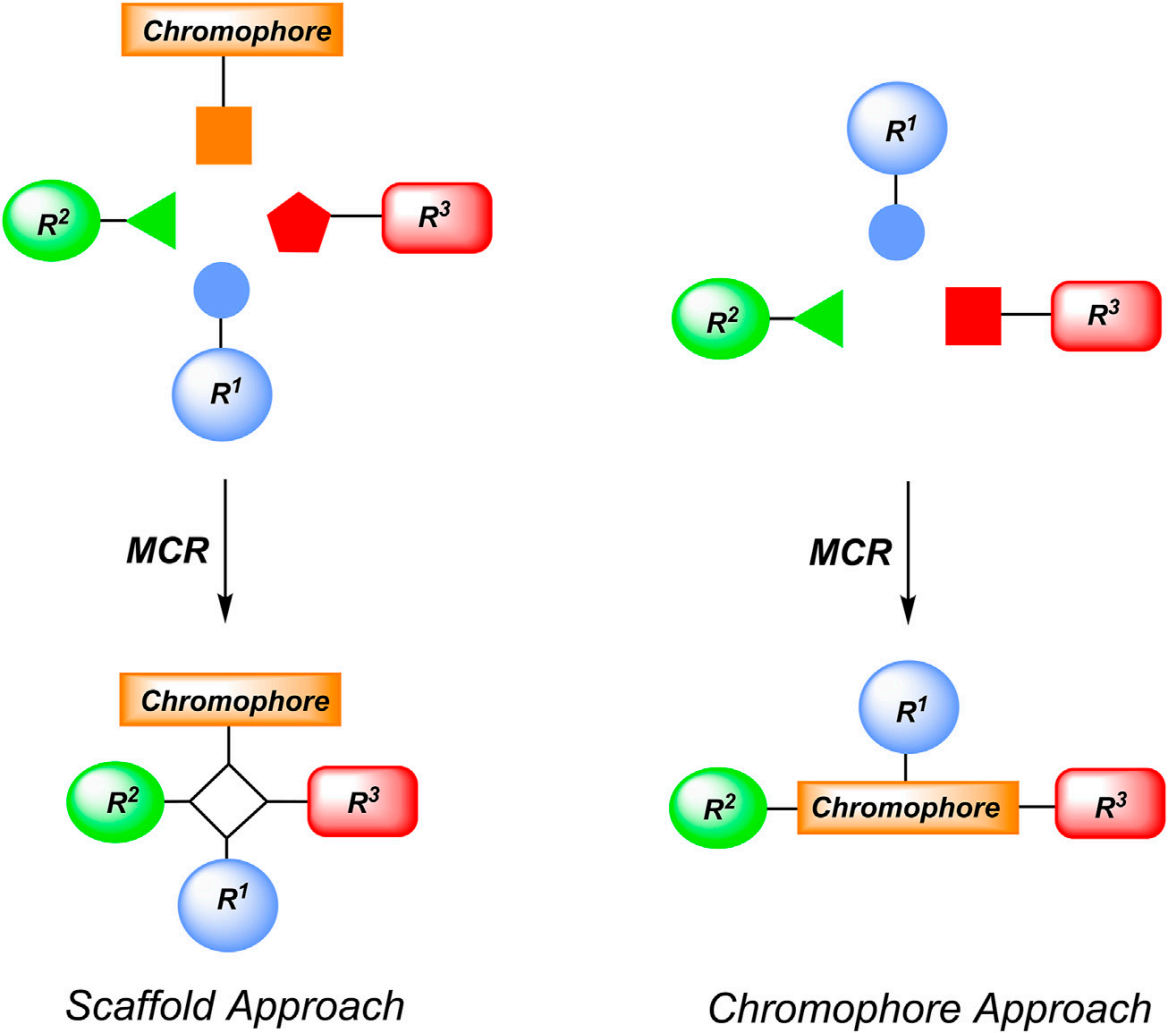
Conceptual MCR formation of functional chromophores by scaffold and chromophore approaches (Reprinted from Levi and Müller (2016b), Copyright (2016), with permission from The Royal Society of Chemistry).

MCRs are generally well suited to generate acyclic or cyclic scaffolds that either bear chromophores as functional substituents (scaffold approach) or can be considered *de novo* formed chromophores (chromogenic approach) (Scheme 1) (Levi and Müller, 2016b). While the former approach allows placing multiple chromophores in close proximity with defined configuration and conformation by the constituted scaffold, also in an electronically non-conjugative fashion, the latter approach is chromogenic and forms the chromophore scaffold of interest with broad structural diversity in a one-pot fashion.

Both approaches are advantageous for advanced chromophore research since large chromophore libraries are rapidly and convergently generated. This allows extensive exploration of electronic properties by electrochemical and photophysical investigation in combination with advanced computational chemistry. Ultimately, the evolving structure-property relationships lead to a more comprehensive understanding and to a rational experimentally founded design of functional chromophores, which are highly requested for high-tech applications.

Guided by the synthetic chromogenic concept of MCR we have structured this review, which updates our previous overview on MCR approaches to functional chromophores (Levi and Müller, 2016b), according to the chromophore classes as defined by their acyclic or (hetero)cyclic central structural elements. Starting from merocyanines, we span the arc from five-to six-membered heterocycles or (hetero)arene and azo chromophores to complexes, discussing predominantly chromophore approaches and some scaffold approaches for illustrating and highlighting in a flashlight fashion most recent developments.

## 2 Merocyanines

Merocyanines are donor-acceptor polyenes (Mishra et al., 2000; Kulinich and Ishchenko, 2009a; Kulinich and Ishchenko, 2009b; Hamer, 2009). Due to the polymethine chain and the resulting conjugated π -system, they can be classified as polymethine dyes. According to the classical definition, the class of merocyanines comprises the streptocyanines and their analogues, in which both the nitrogen atom and the carbonyl group form part of a heterocyclic system. In general, merocyanines exhibit tunable electronic properties (Hamer, 1964; Mishra et al., 2000; Kulinich and Ishchenko, 2009b; Arjona-Esteban et al., 2015; Lenze et al., 2015). By varying the terminal groups, the substituents, and the polymethine chain length, functional dyes can be prepared with applications in optoelectronic and non-linear optical materials, optical information carriers, solar concentrators, electroluminescent devices (Kronenberg et al., 2008; Bürckstümmer et al., 2010; Würthner and Meerholz, 2010), and as fluorescent probes and markers (Valinsky et al., 1978; Williamson et al., 1983; Nandi et al., 2014). Due to their polar nature, merocyanines are generally characterized by absorption and/or emission solvatochromism (Brooker et al., 1951; Reichardt and Welton, 2010). Predominantly, merocyanine dyes are prepared *via* aldol condensation. Only a few multicomponent synthesis routes for the preparation of these dyes are reported (Wurthner, 1999; Wurthner et al., 1999). One of the first MCRs for the synthesis of merocyanine dyes gave access to dye **1** and **2** through a formylation and condensation sequence of thiazoles or indolines with hydroxypyridones and a formylation reagent generated *in situ* from DMF in acetic anhydride in almost quantitative yields in the sense of the chromophore concept. Mechanistically, the DMF in acetic anhydride first reacts with hydroxypyridones and the subsequently formed intermediate reacts with an electron-rich methylene base or heterocycle. In addition, benzothiazole and benzoxazole dyes **3** and **4**, respectively, were obtained in remarkable yields (39%–42%) from the *in situ* generated methylene bases (Supplementary Material S1).

It should be noted that all alkyl-substituted derivatives are characterized by a single extremely narrow absorption band in the visible (*λ*_*max,abs*_ = 495–533 nm). All of the obtained indoline **2**, benzothiazole **3** and benzoxazole dyes **4** display red or orange solid-state luminescence. Only a few of the thiazole dyes **1** fluoresce in solid state. Thiazole dyes **1** absorb light in a range of 528–555 nm. Because of their electronic peculiarity that contributions of the non-polar and the zwitterionic resonance structures are almost identical both in the electronic ground and excited state, these dyes find application in color copy (Gregory, 2012).

A variety of indolone-based merocyanines with a push-pull character on the basis of insertion-alkynylation Michael addition sequence can be synthesized (Muschelknautz et al., 2014; D'Souza et al., 2010; Muschelknautz et al., 2013). Recently, even a white light emitter generated by aggregation-induced double emission (AIDE) in combination with partial energy transfer is postulated (Denißen et al., 2020). Indolone-based merocyanines, with a Boc group, also exhibit interesting photophysical properties such as aggregation-induced emission (AIE) or crystallization-induced emission enhancement (CIEE) (Denissen et al., 2017a).

**SCHEME 2:**
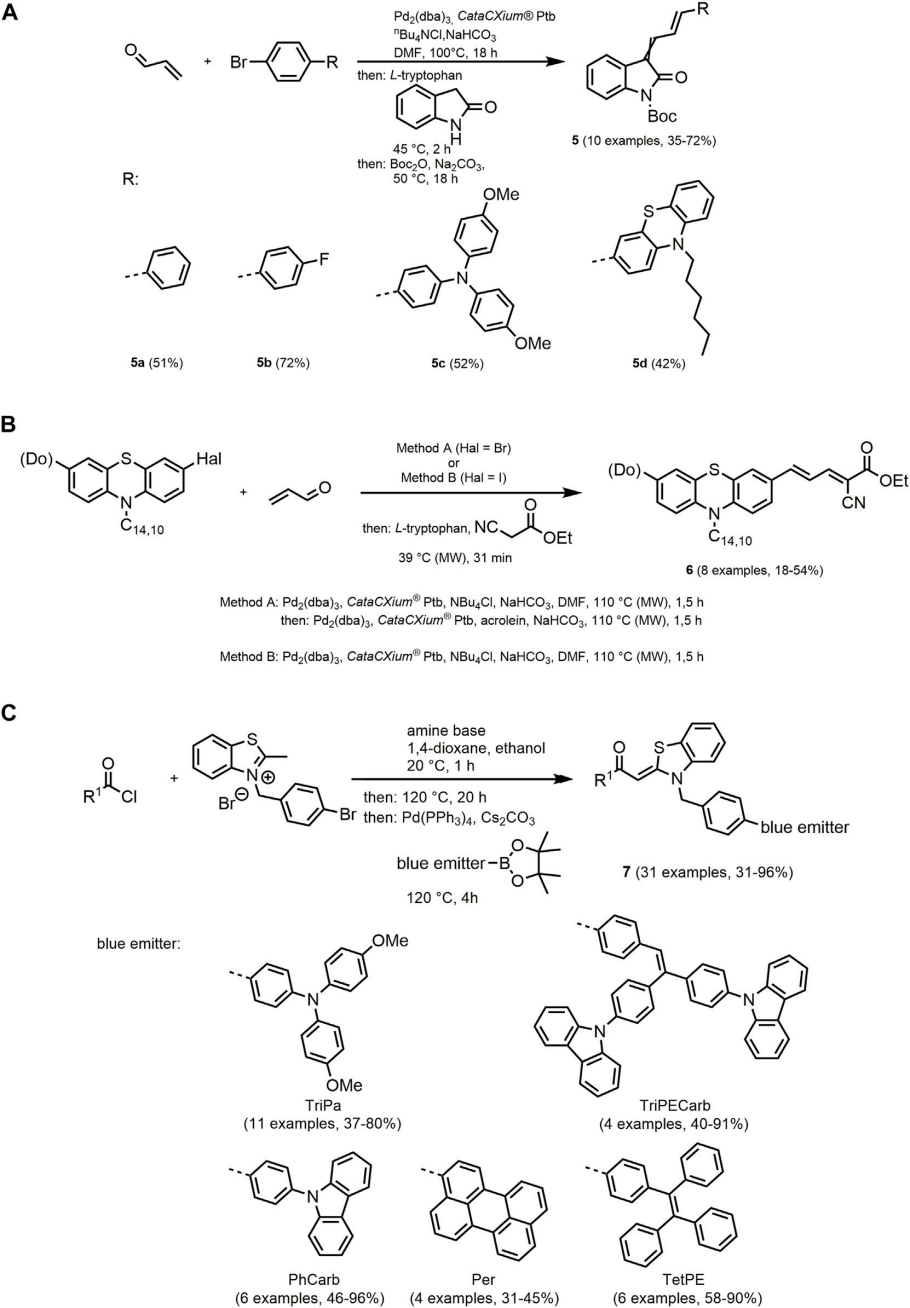
**(A)** Consecutive one pot four-component synthesis of allylidene indolone chromophores **5** by a Heck condensation sequence and selected examples (Wilbert and Müller, 2022). **(B)** Consecutive three-component Heck-Knoevenagel synthesis of merocyanine esters **6** (Stephan et al., 2022). **(C)** Consecutive three-component reaction to synthesize *N*-benzyl aroyl-*S,N*-keteneacetal bichromophores **7** and an insight into the compound library sorted by the employed blue emitters (Biesen et al., 2021).

The Boc-substituted 3-arylallylidene indolones **5** can be synthesized *via* a consecutive four-component approach following the chromophore concept. The synthesis *via* Heck condensation sequence is not stereoselective regarding the allylidene double bond adjacent to the indolone, which exhibits photoisomerization in daylight (Scheme 2A) (Wilbert and Müller, 2022).

Notably, the respective diastereomers of compound **5** share very similar photophysical properties. In general, most of the derivatives display exclusively solid-state emission. Among them, compounds **5a** and **5b** possess the highest fluorescence quantum yields reaching 18%. Furthermore, the oxindole-based merocyanines **5c** and **5d** are also luminescent in solution and exhibit positive emission solvatochromism. Additionally, **5c** exhibits aggregation-induced emission enhancement (AIEE) (Supplementary Material S2).

Using a Heck-Knoevenagel sequence, 7-donor-substituted phenothiazinyl merocyanines **6** are prepared with yields up to 54%. The optimal reaction conditions of the three-component reaction involving heteroaryl halides, acrolein and ethyl cyanoacetate were developed by statistical design of experiments and Bayesian optimization (Scheme 2B) (Stephan et al., 2022).

The phenothiazine-based merocyanines **6** are red-orange dyes that reveal longest absorption bands in a range from 466 to 491 nm. Using the Hammett plot, a correlation of the red shift of the absorption band with increasing donor strength can be proven, which indicates an essential charge transfer character. The fluorescence of the chromophores is located in a range from 730 to 780 nm with Stokes shifts of 6,500–8,200 cm^−1^. The carboxylic acids obtained by saponification of merocyanine esters can be implemented in DSSC and achieve solar cell performance with relative efficiencies up to 93% compared to standard ruthenium dye N3.

Further comparably small merocyanines with outstanding photophysical properties such as AIE behavior and tunable solid-state emission are proposed by chromophores with aroyl-*S,N*-ketene acetal building blocks (Biesen et al., 2020). The previous reported systems were modified by synthesis of bichromophores. For this purpose, blue emitters were introduced after condensing aroyl chlorides and a *N*-(*p*-bromobenzyl) 2-methyl-benzothiazolium salt by a Suzuki coupling of pinacolboronic esters. This three-component one-pot procedure enabled an extraordinarily large substance library of *N*-benzyl aroyl-*S,N*-keteneacetal bichromophores **7** (Scheme 2C) (Biesen et al., 2021).

The synthesized fluorophores show a rainbow-like tuning of the solid-state emission color. Depending on the substitution pattern, a color impression from blue to orange-red can be achieved (Supplementary Material S3).

The variation of both chromophores allows different communication pathways between them. This can result in full or partial energy transfer processes leading to dual emission, or aggregation-induced switching of fluorescence. This selective excitation of either the blue emitter or the aroyl *S,N*-ketene acetal provides specific emission behavior in solution or in aggregates, which can be exploited for application as a p*H* sensor or as a detector of water content in alcoholic beverages.

The bichromophore systems could be further developed by preparing asymmetrically bridged aroyl-*S,N*-ketene acetals and aroyl-*S,N*-ketene acetal multichromophores **8** *via* multiple Suzuki one-pot sequences starting from electron donating *p*-bromo substituted benzylaroyl-*S,N*-ketene acetals. Phenyl and more complex dye systems were accepted as linker systems within the three-component Suzuki reaction. In order to synthesize the multichromophore systems, triborylated benzene and triphenylamine as well as tetraborylated tetraphenylethene were used as linkers (Supplementary Material S4) (Biesen et al., 2022).

Similar to previous synthesized bichromophores, the bridged compounds **8** exhibit AIE and ET properties. The emission behavior in solution and in the solid state as well as starting point for the formation of aggregates can be determined by the employed linker molecule. The interaction of the aroyl *S,N*-ketene acetals and the linker influences photophysical properties such as emission color and intensity, fluorescence quantum yield and lifetime. Furthermore, due to the different combination of the linker and aroyl *S,N*-ketene acetal, partial and complete energy transfer processes as well as different emission behavior upon aggregation (AIE, AIEE and ACQ) is observed. Interestingly, the emission properties of bichromophores **7** and multichromophores **8** can be enhanced by encapsulation in polystyrene particles.

**SCHEME 3:**
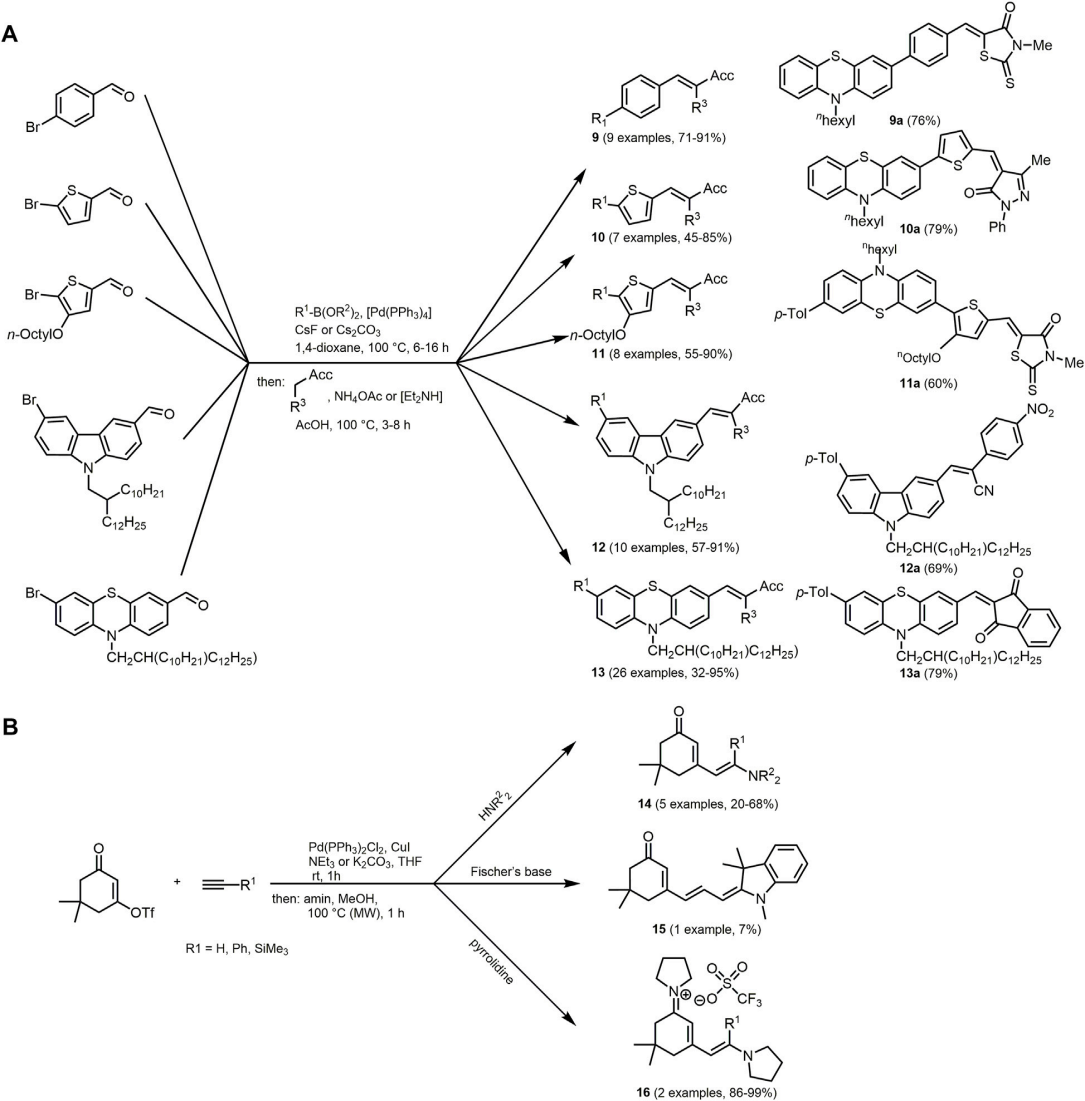
**(A)** Three-component Suzuki–Knoevenagel synthesis of merocyanines **9**–**13** as well as representative derivatives of the substance libraries. The position of the acceptor group is indicated by Acc (Meyer et al., 2021). **(B)** One-pot synthesis of a triflate compound, terminal alkyne and various amines to form cyclohexene-embedded merocyanines **14** and **15** as well as cyanines **16** (Papadopoulos et al., 2022).

Just recently a Suzuki-Knoevenagel condensation sequence was applied to synthesize merocyanines with good DSSC performance in a one-pot fashion (Meyer and Müller, 2020). The consecutive three-component reaction of boronic acids/esters, (hetero)aromatic bromoaldehydes and methylene-active compounds was expanded to a library of 60 donor- π -bridge-acceptor structures with *p*-phenylene- (**9**), thienylene- (**10**), 4-octyl thienylene- (**11**), carbazole- (**12**), or phenothiazine-bridged (**13**) merocyanines (Scheme 3A) (Meyer et al., 2021).

The UV/Vis spectra of merocyanine **9**–**13** exhibit the longest wavelength absorption maxima in a broad spectral range of *λ*_*max,abs*_ = 367–580 nm with molar extinction coefficients ε between 21,000 and 52,000 M^−1^ cm^−1,^ which accounts to a dominant charge transfer transition from the donor to the acceptor part. The emission maxima can be detected in a range of 412–668 nm with Stokes shifts ranging from 1,200 to 8,000 cm^−1^ (0.147–0.990 eV). The energy of the hypothetical *E*_*0-0*_ transition determined from the intersection of the absorption and emission bands represents the optical band gap and lies between 2.083 and 3.197 eV. A correlation analyses by plotting the optical band gaps *E*_*0-0*_ against the first oxidation potentials *E*_*1/2*_ of redox active systems of consanguineous series furnishes linear correlations and, by extension, two parameter correlations (oxidation potential and emission maximum) with the optical band gaps. Thus, given this planar correlation, for a number of merocyanines optical band gaps can be predicted based on the first oxidation potentials and emission maxima.

Consecutive three-component alkynylation addition sequences can be performed to synthesize cyclohexene-embedded merocyanines **14** and **15**. A stronger nucleophilic amine moiety like pyrrolidine can be employed to synthesize cyanines **16** in excellent yields *via* a pseudo four-component synthesis by carbonyl condensation of the heterocyclic amine (Scheme 3B) (Papadopoulos et al., 2022).

Merocyanines **14** and **15** and cyanines **16** are obtained as yellow to orange solids. The prepared merocyanine derivatives are found to be non-luminescent in solution and in solid state, whereas the cyanines exhibit luminescence with low quantum yield of 1%. The absorption maxima of **16** are located at 443 and 459 nm with absorption coefficients over 110·10^3^ M^−1^cm^−1^ for the two derivates. The absorption maxima of the merocyanines **14**/**15** and component **16** are located at 365–390 nm and 442 nm, respectively. Due to the extension of the system, the red shift of the absorption band can be explained.

## 3 Five-membered heterocycles

### 3.1 Oxygen heterocycles

Furans are found in many natural products (Keay and Dibble, 1996) and as a result of their biological activity, they are often structural moieties in pharmaceuticals (Iyer and Gopalachari, 1973; Dikshit et al., 1974; Durani et al., 1989; Dikshit et al., 1991; Nieves-Neira et al., 1999; Mortensen et al., 2001; Chène, 2003; Issaeva et al., 2004; Thuita et al., 2008; Wenzler et al., 2009; Barrett and Croft, 2012). The Molisch test is a colometric detection method that can be used to detect carbohydrates that have been converted into furfuraldehydes by thermal acid degradation. This detection is carried out *via* reaction with α-naphthol and results in a violet coloration (Molisch, 1886). But aside from their pharmacological importance, they can also be considered as photonic chromophores (Liu and Luh, 2002). In general, many furan heterocycles exhibit strong fluorescence. Due to their excellent semiconducting properties, they are found in organic solar cells (Zheng and Huo, 2021). In addition, furan possesses strong non-linear optical properties which makes it useful in a number of non-linear optical applications (Kamada et al., 2000; Gündüzalp et al., 2016). Classic synthetic routes for the production of substituted furans rely either on cyclocondensation of dicarbonyl compounds or on furan ring substitution (Zeni and Larock, 2004; Brown, 2005; Duc, 2019). The preparation of 2,5-disubstituted furans is commonly carried out *via* a Paal-Knorr reaction in the sense of a cyclocondensation of 1,4-diketones (Amarnath and Amarnath, 1995), yet a consecutive multicomponent approach to symmetrically 2,5-disubstituted furans *via* a Sonogashira-Glaser addition-cyclization sequence is known (Klukas et al., 2014).

**SCHEME 4:**
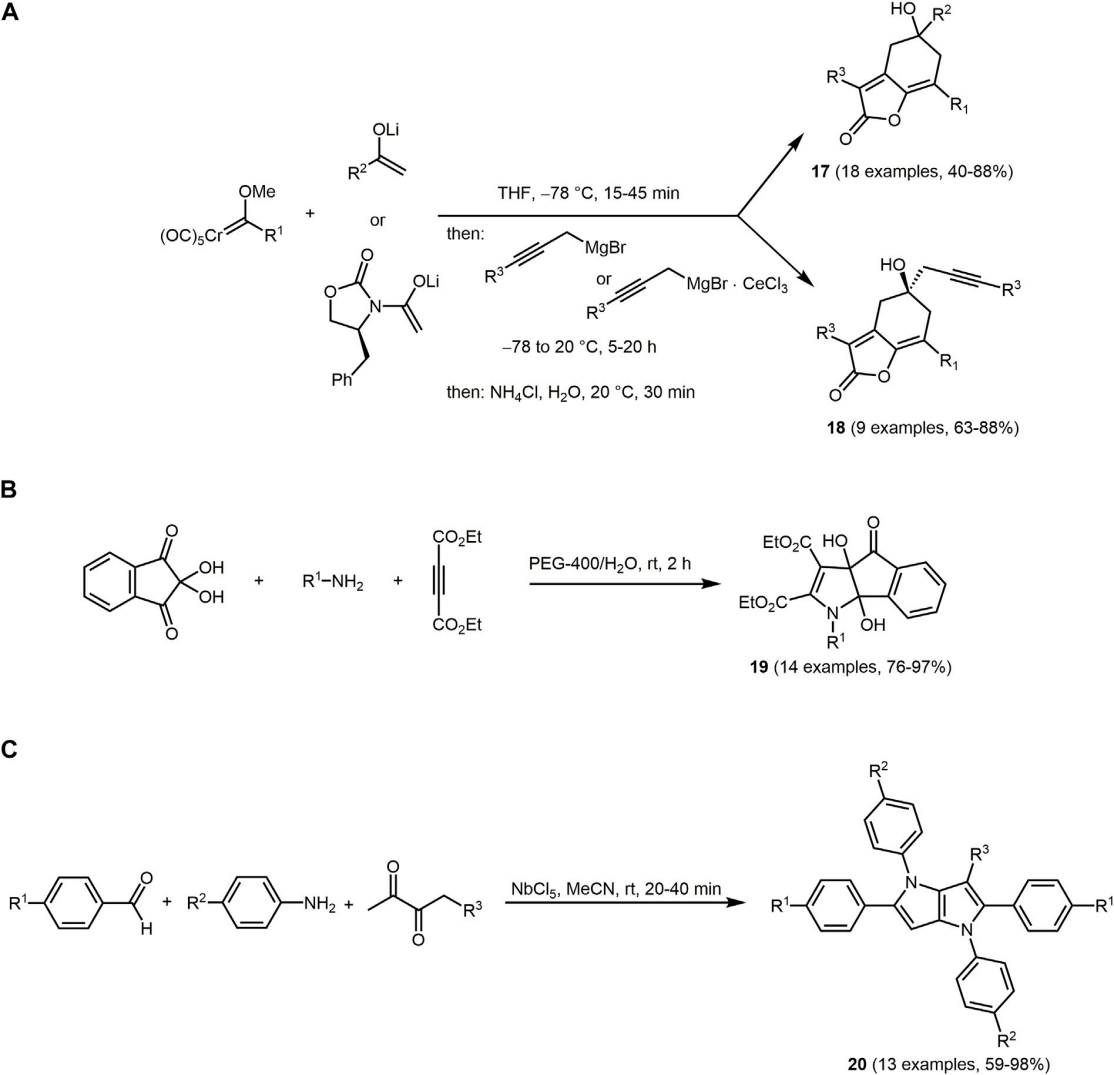
**(A)** One-pot synthesis of 6-5-bicyclic γ-alkylidene-2-butenolides **17** and **18** from ketone lithium enolates or from imide lithium enolates (Suero et al., 2012). **(B)** Three-component synthesis of highly functionalized dihydroindeno[1,2-*b*]pyrrole fluorophores **19** (Mal et al., 2018). **(C)** Pseudo five-component synthesis of tetraaryl-1,4-dihydropyrrolo-[3,2-*b*]pyrrole derivatives **20** (Martins et al., 2018).

Butenolides are unsaturated lactones consisting of a dihydrofuran-2-one group. They are important structural building blocks in natural products (Seitz and Reiser, 2005; Roethle and Trauner, 2008; Kitson et al., 2009; Igarashi et al., 2011) and often possess high biological activity such as antibiotic, anticancer and antitumor (Shiomi et al., 2005; Wu et al., 2005; Fei et al., 2007; Liu et al., 2008; Isaka et al., 2009; Wang et al., 2009; Kitani et al., 2011; Uchida et al., 2011; Chen et al., 2012; Csuk et al., 2012). The coupling of a chromium methoxycarbene complex with a ketone or an imide lithium enolate followed by a propargylic organomagnesium reagent lead to the synthesis of novel hydroxy-substituted bicyclic [4.3.0]-galkylidene-2-butenolides **17** and **18** (Scheme 4A) (Suero et al., 2012). Upon modulating substituents on the starting materials, high diversity and complexity of the bicycles can be realized. For instance, alkyl, aryl, heteroaryl, alkenyl and alkynyl groups are well tolerated in the bicyclic compound **17** as a group R^2^ resulting from the ketone component. The synthesis of compound **18** proceeds enantioselectively due to the inserted chiral imide enolate auxiliary.

Both heterocycles embed the γ-(2-arylalkylidene)-2-butenolide fluorophore and the bicycles **17** and **18** fluoresce intensively blue in solution with large Stokes shifts.

### 3.2 Nitrogen heterocycles

Pyrrole is the five-membered azol and appears as a structural core in many biologically active molecules with antibacterial (Daidone et al., 1990), antifungal (Kaiser and Glenn, 1972), anti-inflammatory (Battilocchio et al., 2013), antioxidant (Meshram et al., 2010), antitumour and ionotropic properties (Jonas et al., 1993). Besides, pyrroles naturally and non-naturally occurring pyrroles are *per se* interesting as functional dyes (Jonas et al., 1993; Fürstner et al., 1998; Fürstner, 2003; Jones and Bean, 2013; Khajuria et al., 2016). Thus, aryl-substituted pyrrole derivatives with pronounced AIE properties are known (Feng et al., 2010; Han et al., 2012; Shi et al., 2012) and in addition, for instance, indenopyrrole derivatives can serve as Al(III) selective OFF-ON chemosensors for bioimaging in live human hepatocellular carcinoma cells (Mal et al., 2018). For this purpose, the reaction of ninhydrin, primary amines and dialkyl acetylenedicarboxylates furnishes a series of dihydroindeno[1,2-*b*]pyrrole derivatives utilizing the chromophore approach (Scheme 4B). The selected green solvent is water and PEG-400 [polyethylene glycol (400)] acts as a phase transfer catalyst. First, a nucleophilic enamine diester is formed upon reaction of the amine and dialkyl acetylenedicarboxylate, which nucleophilically attacks the carbonyl center of the ninhydrin. After water elimination, intramolecular cyclization and tautomerization results in the formation of indeno[1,2-*b*]pyrrole derivatives **19**.

Chromophores **19** exhibit a weak emission in the range of around 485 and 515 nm. However, the fluorescence intensity increases selectively in the presence of Al(III) ions due to CHEF (chelation-enhanced fluorescence). Complexation ensues between the Al(III) ions and presumably the two *syn*-O-atoms of the functional alcohol groups, which can be experimentally verified by ^1^H NMR as well as *via* calculations (Sun et al., 2012; Jo et al., 2016; Naskar et al., 2018).

Several multicomponent syntheses of pyrroles have been reported (Balme, 2004; Estevez et al., 2010; Estévez et al., 2014). For instance, tetraaryl-1,4-dihydropyrrolo[3,2-*b*] pyrroles **20** are formed *via* Mannich reaction of benzaldehydes, anilines and β-diketone derivatives in the sense of a chromophore approach (Scheme 4C) (Martins et al., 2018). The *in situ* generated Schiff base reacts with the enol form of butane-2,3-dione. Then, the cyclic enamine intermediate adds to another Schiff base. The pyrrolopyrrole is finally formed cyclization and subsequent oxidation (Janiga et al., 2013). These compounds can be classified as heteropentalenes which consist of two fused heterocyclic five-membered rings (Gribble and Joule, 2009) with 10 π-electrons and, thus, elevating them for further usage in electronic devices (Biswas et al., 2016). Chromophores **20** are promising candidates as sensitizing dyes in optoelectronic applications. For the MCR synthesis of compounds **20**, niobium pentachloride is used as a Lewis acid catalyst. The reaction proceeds *via* two competing mechanisms, which can be controlled by the reactivity of the selected benzaldehyde and aniline derivatives.

Most components absorb between 294 and 382 nm and emit around 420 nm. As said, the photophysical properties suggest that these materials can be used as sensitizing dyes in optoelectronic devices. The modulation of the optical properties can be particularly controlled by the substituent R^1^ benzaldehyde derivative. For the electron-withdrawing substituent NO_2_, the absorption bands redshifts to the region between 500 and 600 nm. In contrast, the electron-releasing *p*-methyl substituent CH_3_ causes a blueshift of the absorption bands.

**SCHEME 5:**
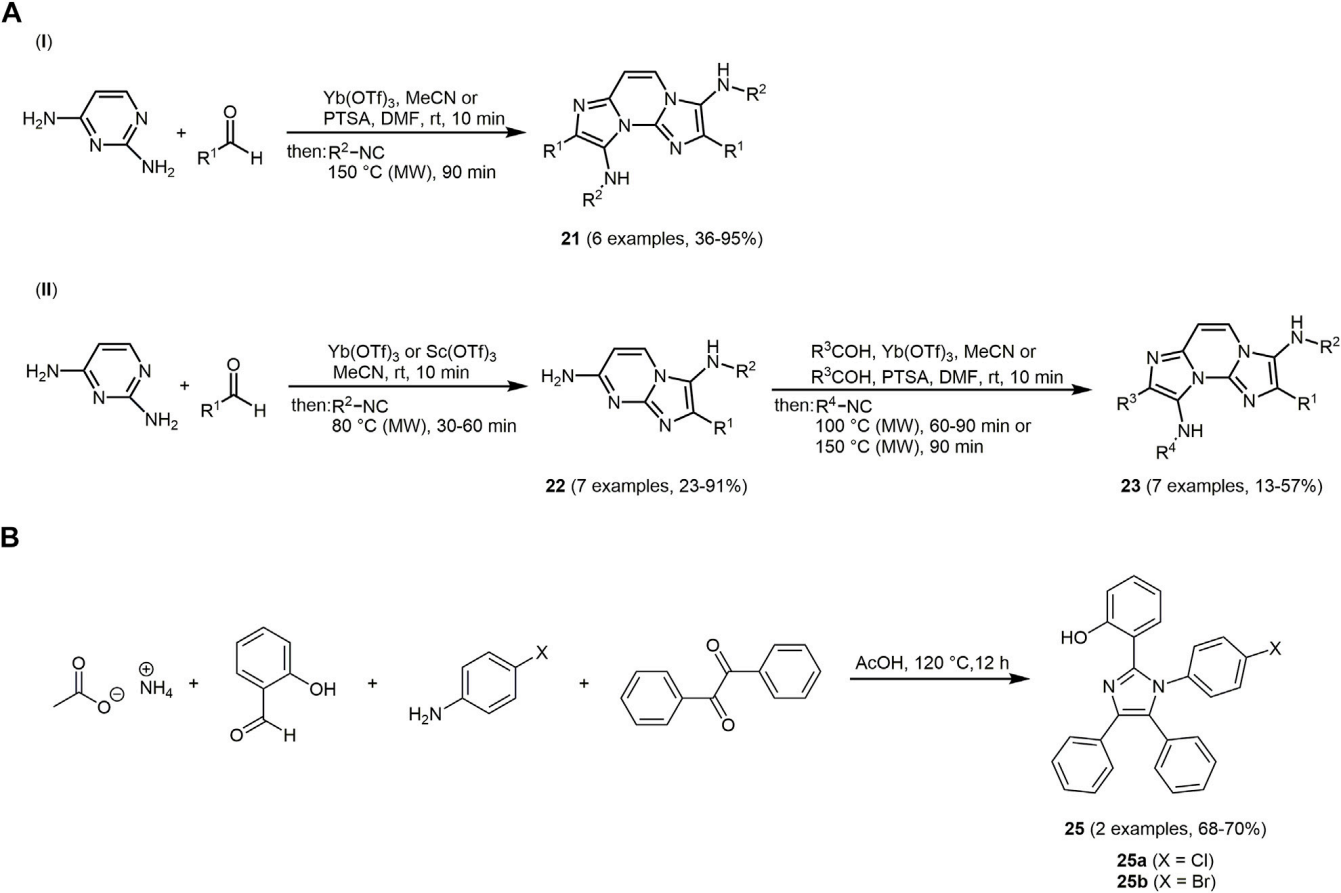
**(A)** Synthesis of GBB products **21** and **23** *via* twofold GBBR of 2,4-diaminopyrimidine in a direct (I) and a sequential approach (II) (Ghashghaei et al., 2018). **(B)** Debus-Radziszwski multicomponent reaction to form imidazole-based fluorophores **25** (Somasundaram et al., 2018).

Imidazole is a key structural motif of considerable importance in biomolecules as represented by the essential amino acid histidine (Grimmett et al., 1984; Luca, 2006; Bellina et al., 2007). Imidazoles have been shown to possess antibacterial (Khabnadideh et al., 2003; Khalafi-Nezhad et al., 2005), antifungal (Pestellini et al., 1987; Jones et al., 1990; Silvestri et al., 2004), farnesyltransferase inhibitory (Curtin et al., 2003; Lin et al., 2003; Tong et al., 2003), and anti-inflammatory activity properties (Raingeaud et al., 1995; Beyaert et al., 1996) or function as selective ligands at histamine receptors (Ganellin et al., 1996; De Esch et al., 1999; Elz et al., 2000). Imidazole-fused heterocycles can exhibit interesting fluorescence properties such as excited-state intramolecular proton transfer (ESIPT) (Heller and Williams, 1970; Goodman and Brus, 1978; Kasha, 1986; Park et al., 2005; Skonieczny et al., 2012; Ali et al., 2015). In addition, some non-linear optical (NLO) imidazole-based chromophores (Stähelin et al., 1992; Bu et al., 1996; Santos et al., 2001; Wang et al., 2002) as well as donor-acceptor chromophores are known (Murata et al., 2007; Chang et al., 2008; Chang et al., 2009; Kulhánek et al., 2011). The Groebke-Blackburn-Bienaymé reaction (GBBR) represents an MCR approach to imidazoazines by acid-catalyzed condensation of aminoazines, aldehydes and isocyanides (Bienaymé and Bouzid, 1998; Blackburn et al., 1998; Groebke et al., 1998). Using the concept of GBBR, heterocyclic scaffolds with adjustable properties can be prepared. Chromophores **21** are synthesized *via* a double GBBR of 2,4-diaminopyrimidine and a variety of aldehydes and isocyanides in moderate to good yields following the chromophore concept (Scheme 5AI) (Ghashghaei et al., 2018). The process can also be conducted in a sequential fashion to give unsymmetrically substituted chromophores **23** *via* the intermediate GBB monoadducts **22** (Scheme 5A II). The high selectivity is rationalized by preferential formation of an imine from the amino group at 2-position in the initial step of the GBB. Likewise, various polyaminopolyazines such as 2,4-diaminoquinazolines and 3,6-diaminopyridazines as substrates are equally successfully transformed.

The emission maxima of selected GBBR dyes **21** and **23** are found in a narrow range from 447 to 460 nm. By the substituent diversity on the GBB cores, the emission can be tuned. Thus, a redshift in absorption and emission wavelengths occurs by expanding the π-electron conjugation with conjugated (hetero)aryl groups and their modification. For instance, the orange bispyridinium salt **24** is prepared by alkylation of the pyridine ring of yellow chromophore **21a** (Supplementary Material S5). While compound **21a** shows no specific interaction with quadruplex DNA structures, the bispyridinium salt **24** reveals significant strong interaction and can be potentially applied as selective binder for the further development of new anticancer drugs.

A further established approach for the synthesis of imidazoles is the Debus-Radziszwski reaction (Debus, 1858; Radzisewski, 1882). Upon reaction of ammonium acetate, salicylaldehyde, 4-chloro/bromoaniline and benzil in acetic acid, 2-(1-(4-chlorophenyl)-4,5-diphenyl-1*H*-imidazol-2-yl)phenol **25a** and 2-(1-(4bromophenyl)-4,5-diphenyl-1*H*-imidazol-2-yl)phenol **25b** are obtained in good yields (Scheme 5B) (Somasundaram et al., 2018).

The synthesized imidazole chromophores **25** are blue fluorescent and show excited state proton transfer (ESIPT). In the solid state as well as in solution, intense fluorescence is exhibited due to the four phenyl rings on the imidazole core limiting the intermolecular interactions between neighboring molecules.

**SCHEME 6:**
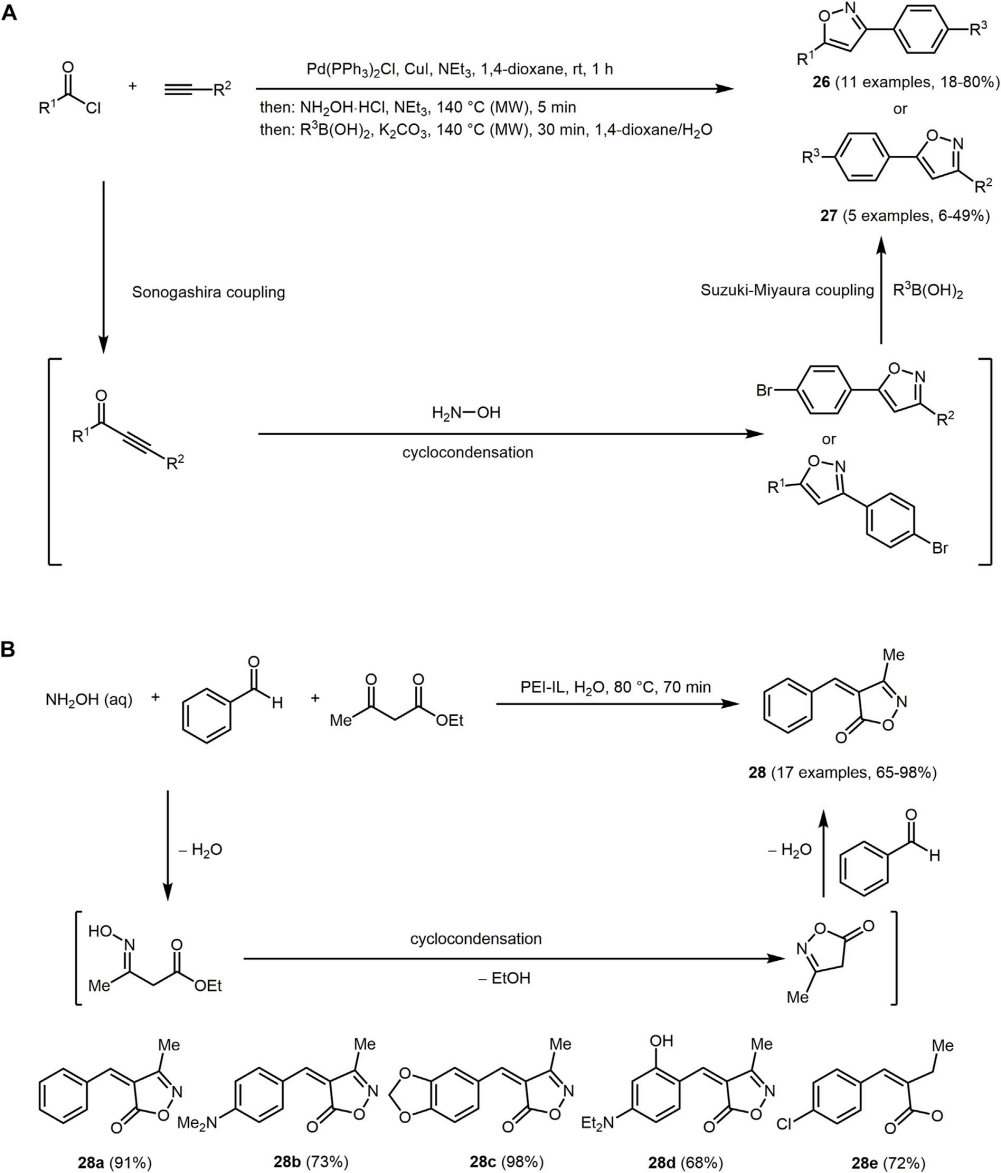
**(A)** Synthesis of biaryl-substituted isoxazoles **26** and **27** *via* a coupling-cyclocondensation-coupling (C^3^) sequence (Deden et al., 2020). **(B)** Enzyme-catalyzed three-component isoxazol-5(4*H*)-one **28** synthesis and selected fluorophores (Oliveira et al., 2021).

Isoxazoles rarely occur in nature, yet, these heterocycles possess enormous bioactive properties (Palmer and Venkatraman, 2003) and are therefore used in therapeutics (Kumbhare et al., 2012; Joshi et al., 2017; Pairas et al., 2017). However, their photophysical properties are still largely unexplored (Irfan et al., 2016; de Brito et al., 2018; Sato et al., 2020). Some one-pot methods for the formation of isoxazoles have been reported (Merkul and Müller, 2006; Kaim et al., 2009; Wachenfeldt et al., 2013). Just recently, a sequentially palladium catalyzed consecutive four-component coupling-cyclocondensation-coupling (C^3^) synthesis of isoxazoles from aroyl chloride, alkyne, hydroxylamine and boronic acids has been disclosed (Scheme 6A) (Deden et al., 2020). Fluorescent biaryl-substituted isoxazoles **26** and **27** are obtained in the sense of a chromophore approach. Indeed, *p*-bromophenyl acetylene or *p*-bromobenzoyl chloride can be successfully employed in a sequential catalyzed Sonogashira-cyclocondensation-Suzuki process.

The representatives with donor-acceptor substitution pattern strongly fluoresce in solution (Supplementary Material S6). The absorption spectrum of **26a**, which contains the biaryl substituent in 3-position, is hypsochromically shifted (*λ*_*max,abs*_ = 294 nm). Compound **26a** exhibits the highest molecular absorption coefficient (ε = 66,000 M^−1^cm^−1^) and Stokes shift (Δ $\tilde{\nu }$ = 10,400 cm^−1^) in this series of biaryl-substituted isoxazoles. However, the quantum yield of **26a** (*Φ*_*F*_ = 0.17) is considerably lower compared to 5-biaryl-substituted isoxazoles **27**, which range from 0.62 to 0.86. Emission maxima of compounds **26a–c** are detected at 376, 411, and 554 nm, respectively. The bathochromic shift of **27b** is caused by the electron-withdrawing cyano substituent in comparison to the trifluoromethyl substituent of **26a**. The strong bathochromic shift of compound **27c** results from the strong electron-donating dimethylamino donor.

Another interesting catalytic approach for the generation of isoxazol-5(4*H*)-one is catalysis by enzymes. Polyethylene imine (PEI) derivatives are synthetic enzymes with good catalytic activities for various reactions (Vasylyev et al., 2006; Abu-Reziq et al., 2008; Avenier and Hollfelder, 2009). The benefit of this catalyst system is that it can be recycled up to 15 times without any noticeable loss of catalyst activity. Mechanistically, the condensation reaction of hydroxylamine and ethyl acetoacetate is followed by cyclization. The resulting isoxazolones undergo a Knoevenagel-type condensation with aromatic aldehydes to form isoxazol-5(4*H*)-ones **28** in good to excellent yields (Scheme 6B) (Oliveira et al., 2021).

The shown derivatives **28** fluoresce in solution and they also display positive solvatochromicity. Thus, small Stokes shifts are observed in non-polar solvents and medium to large Stokes shifts in polar solvents. In general, the chromophores have large molar extinction coefficients and low fluorescence quantum yields. In particular, the fluorescent derivative **28d** represents a promising candidate as a probe for bioimaging due to its ability to selectively stain early endosomes in living cells.

**SCHEME 7:**
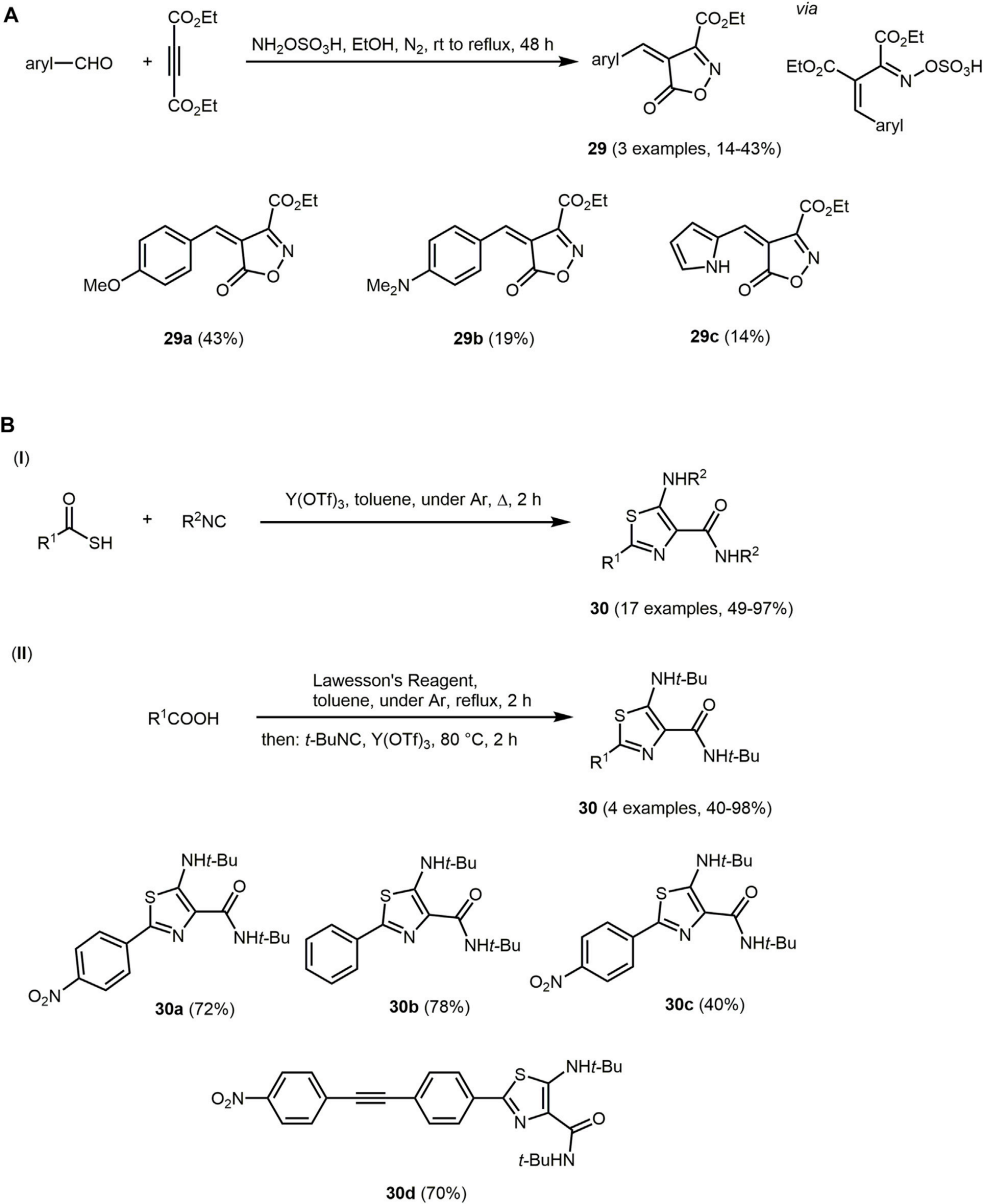
**(A)** Synthesis of isoxazol-5-one **29** *via* a two-step one-pot reaction and the three obtained fluorophores (Tasior et al., 2021). **(B)** One-step synthesis of 5-amino-4-carboxamidothiazoles **30** based on the chromophore approach and the synthesized chromophores (Tong et al., 2017).

Another push-pull chromophore, where the isoxazole scaffold acts as the electron acceptor and the aromatic ring as the donor, is 4-(arylmethylene)-5-oxo-4,5-dihydroisoxazole-3-carboxylic acid **29**. The reaction proceeds *via* a two-step one-pot reaction starting from aromatic aldehydes, diethyl acetylenedicarboxylate and hydroxylamine-*O*-sulfonic acid (Scheme 7A) (Tasior et al., 2021). The reaction is initiated by a Michael addition of hydroxylamine to diethyl acetylenedicarboxylate. The formed enamine then reacts with aromatic aldehydes and the isoxazole derivatives are obtained by elimination of the sulfur trioxide and subsequent transesterification.

All isoxazole derivates **29** are non-emissive, probably due to thermal relaxation caused by rotation of the methine bridge. The absorption maxima of **29b** and **29c** are bathochromically shifted (*λ*_*max,abs*_ = 491 and 435 nm, respectively) in comparison to the maxima of **29a** (*λ*_*max,abs*_ = 400 nm) caused their stronger electron-donating character.

Another five-membered ring containing a nitrogen and a sulfur atom is thiazole. The thiazole core is found in naturally occurring compounds such as vitamin B_1_, penicillins and dolastatin analogues. The latter are cytostatic drugs (Maderna et al., 2014), which can possibly be used in the treatment of cancer (Bhat et al., 2009; Halasi et al., 2010; Anuradha et al., 2019; Sharma et al., 2020). In general, thiazoles show biological activity and are therefore of great interest for medicinal chemistry (Kashyap et al., 2012; Mahmoodi and Ghanbari Pirbasti, 2016; Hussein and Turan, 2018). Furthermore, organic semiconductors based on thiazole for organic electronics are known (Lin et al., 2012a; Lin et al., 2012b). The synthesis of thiazoles proceeds *via* MCR, for example *via* the Hantzsch reaction or *via* cyclization/oxidation of a corresponding peptide precursor in a biomimetic approach (Hantzsch, 1881; Evans et al., 1979; Boden and Pattenden, 1994; Videnov et al., 1996). Thiazoles can also be prepared *via* condensation of thiocarboxylic acids with isocyanides (Scheme 7B I) (Tong et al., 2017). 5-Amino-4-carboxamidothiazoles **30** obtained by the triflate-catalyzed reaction display interesting fluorescence properties as potential ESIPT chromophores. Alternatively, thiazole-based chromophores **30** are also produced by reacting a suspension of carboxylic acid with Lawesson’s reagent in boiling toluene followed by addition of an isocyanide and Y(OTf)_3_ in a one-pot fashion (Scheme 7B II).

5-Amino-4-carboxamidothiazoles **30** (*λ*_*max,abs*_ = 333–460 nm) possess high extinction coefficients (ε > 10^4^ M^−1^ cm^−1^) and fluoresce with violet, blue, green to yellow color depending on the substitution pattern (*λ*_*max,em*_ = 393–558 nm). Upon UV excitation, compound **30a** emits in the green region, **30b** in the in violet to blue region and **30c** as well as **30d** in the yellow region. An aryl substituent in 2-position with electron-withdrawing and electron-donating antiauxochromes and auxochromes can significantly increase the quantum yield. The quantum yield can also be increased by expanding the conjugated system *via* group R^1^. The insertion of a multiple bond between the thiazole ring and further arenes in substituent R^1^ causes a bathochromic shift. In particular, nitro-phenyl substituted thiazoles can be excited with visible light and fluoresce with large Stokes shifts, high quantum yields and pronounced solvatochromism. These dyes have potential for the double ESIPT process.

**SCHEME 8:**
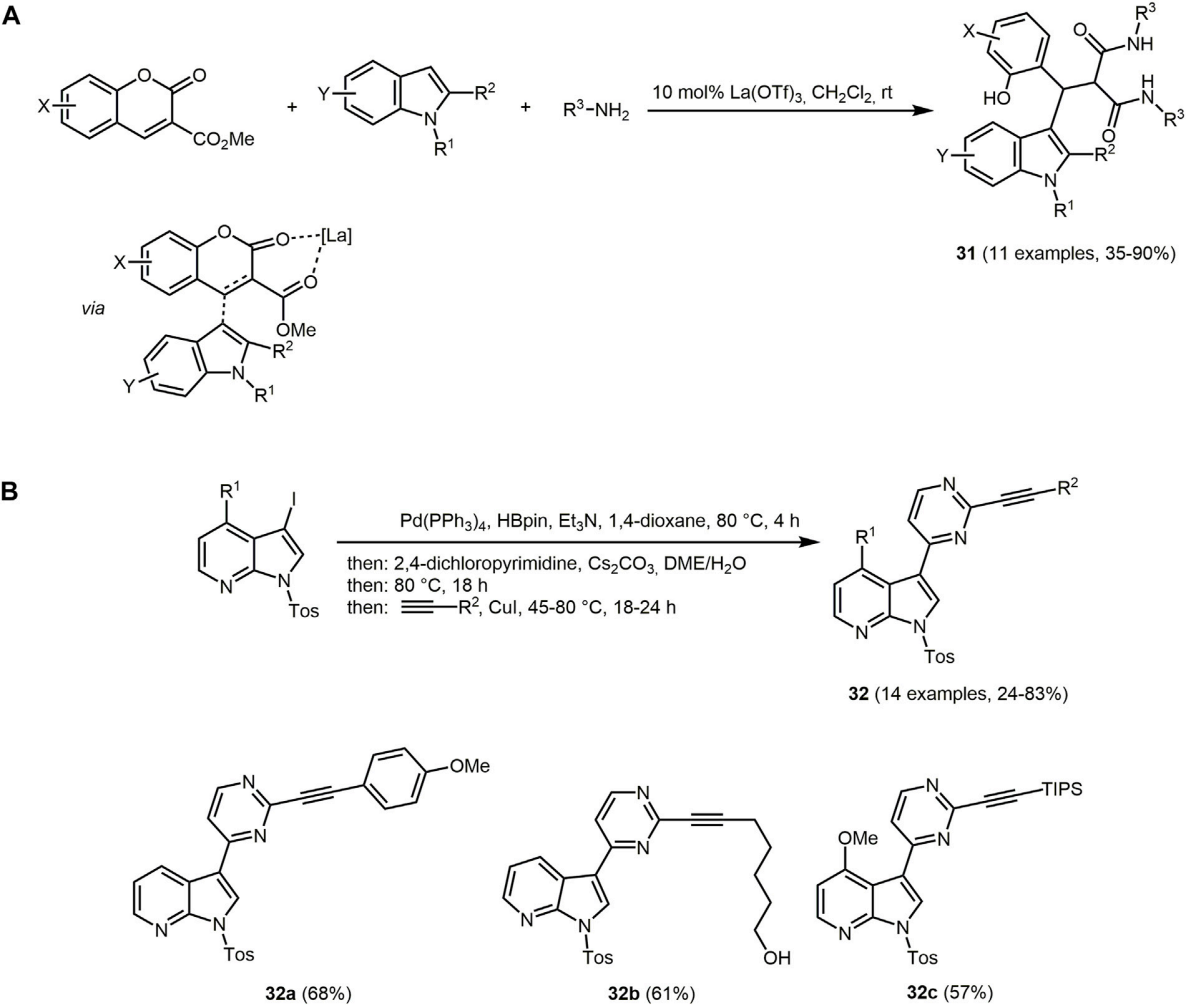
**(A)** Three-component synthesis of indolylmalonamides **31** (Jennings et al., 2016). **(B)** Sequentially catalyzed consecutive three-component Masuda–Suzuki–Sonogashira synthesis of 2-alkynyl-4-(7-azaindol-3-yl) pyrimidines **32** and selected fluorophores (Drießen et al., 2021).

Indoles are desirable due to their widespread medical use (Kang et al., 2009; Kochanowska-Karamyan and Hamann, 2010; Kaushik et al., 2013; Álvarez et al., 2013; Zhang et al., 2015a; Zhang et al., 2015b; Parisi et al., 2015; Hu et al., 2017) and their occurrence in many natural products (Lindquist et al., 1991; Pettit et al., 2002; Cruz-Monserrate et al., 2003; Simon and Petrášek, 2011). For instance, indole constitutes the core of many alkaloids, some hormones and also dyes. Among indole dyes indigo is the most prominent representative (Amat et al., 2011). Considering indoles photophysically, the heterocycle displays fluorescence and strong phosphorescence (Bünau and Birks, 1970). The absorption and emission maxima of indole derivatives in aqueous solution lie typically at 270 and 355 nm, respectively (Bridges and Williams, 1968). Similar absorption and emission values are found for indolylmalonamides **31**. In addition, when irradiated with long-wave UV light ( λ _*exc*_ = 366 nm) chromophores **31** with high molar absorptivity fluoresce and exhibit large Stokes shifts. The synthesis of indolylmalonamides **31** are performed *via* a lanthanum (III)-catalyzed three-component amidation reaction of coumarin-3-carboxylates, indoles and amines in the sense of the chromophore approach (Scheme 8A) (Jennings et al., 2016). After Lewis acid-catalyzed Michael addition indolylmalonamides **31** are obtained by Lewis acid-catalyzed amidation.

The sequentially Pd-catalyzed three-component Masuda-Suzuki-Sonogashira reaction combines three Pd-catalyzed processes (borylation, arylation, and alkynylation) in a one-pot fashion to give fluorescent 2-alkynyl-4-(7-azaindol-3-yl) pyrimidines **32** as illustrated with *N*-tosyl 3-iodo-7-azaindoles, 2,4-dichloropyrimidines and terminal alkynes as starting materials (Scheme 8B) (Drießen et al., 2021).

The UV/Vis spectra of 2-alkynyl-4-(7-azaindol-3-yl) pyrimidines **32** show absorption maxima at *λ*_*max,abs*_ = 293–296 nm with molar extinction coefficients ranging between 23,100 and 43,900 L mol^−1^ cm^−1^ for aliphatic substituents and 48,000 and 77,000 L mol^−1^ cm^−1^ for aromatic substituents, respectively. Electron-donating substituents R^2^ red-shift the absorption maxima to longer wavelengths. Most of the compound fluoresce with emission maxima at about 447 nm with high Stokes shifts (Δ $\tilde{\nu }$ = 10,300–11,900 cm^–1^). It should be noted that this method provides an efficient access to alkynyl meriolins, a new biological active class of potential apoptosis inducers.

**SCHEME 9:**
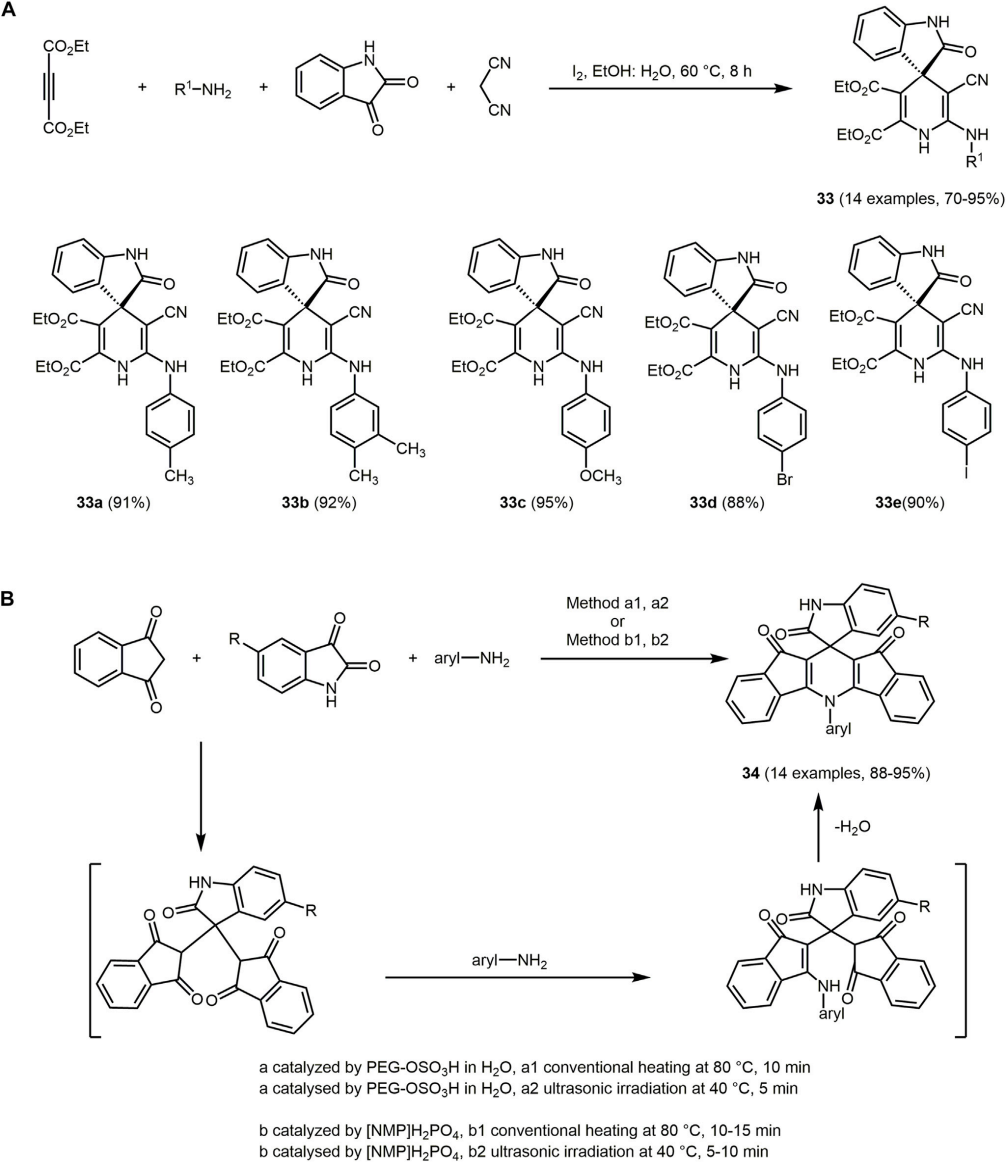
**(A)** Amine-appended spiro[indoline-3,4′-pyridine] ON–OFF chemosensor **33** and selected derivates (Mondal et al., 2018). **(B)** MCR synthesis of spiro[diindenopyridine-indoline]triones **34** with different catalyst systems (PEG-OSO_3_H or [NMP]H_2_PO_4_) under conventional heating and ultrasonic irradiation (Sindhu et al., 2015).

Amine-appended spiro[indoline-3,4′-pyridines] **33**, for example, can be applied as ON-OFF chemosensors for Cu(II) ions *via* a fluorescence response. These chemosensors show high selectivity and have already been applied for imaging Cu(II) ions in human hepatocellular liver carcinoma cells. Spiro[indoline-3,4'-pyridine] **33** are prepared *via* a one-pot four-component reaction involving dialkyl but-2-ynedioate, primary amines, isatin, and malononitrile (Scheme 9A) (Mondal et al., 2018). The environmental benign reaction is catalyzed by iodine and carried out in aqueous ethanol solution. The proposed mechanism suggests activation of the C-3 carbon of isatin by iodine. The isatin-iodine complex reacts with malononitrile *via* Knoevenagel condensation. In the presence of the iodine catalyst, the nucleophilic attack of the primary amine occurs. Addition of dialkyl but-2-ynedioate initiates the intramolecular Michael addition. Subsequently, chromophores **32** are formed by proton tautomerization.

The chromophores **33** display strong emission in DMSO in a range of 458–493 nm with large Stokes shifts (Δ $\tilde{v}$ = 5,335–11,641 cm^−1^). In the presence of Cu(II) ions the fluorescence decays and the color change can be observed by the naked eye. Chemosensor **33d** with the highest quantum yield (0.95) has successfully been used for *in vitro* fluorescence cell imaging of Cu(II) ions in human hepatocellular liver carcinoma cells.

Further spiro indoles **34** can also be generated under sustainable conditions. The three-component reaction of 1,3-indanediones, isatins, and aromatic amines is catalyzed either by PEG-OSO_3_H or by [NMP]H_2_PO_4_ to produce spiro[diindenopyridine-indoline]triones **34** under conventional heating and ultrasonication (Scheme 9B) (Sindhu et al., 2015). The PEG-OSO_3_H is a polymeric acid-surfactant based catalyst that can be recycled and reused without significant loss of activity. Implementing of acidic ionic liquid [NMP]H_2_PO_4_ as a catalyst, solvents are not required as it also functions as the medium. The condensation reaction proceeds with a variety of aromatic amines and different isatins with excellent yields (88%–95%) following the chromophore approach.

All dyes **34** are deep red with broad absorption bands with maxima between 264 and 274 nm and they show strong fluorescence in methanol in a range of 282–596 nm with large Stokes shifts (Δ $\tilde{v}$ = 11,000–13,000 cm^−1^).

**SCHEME 10:**
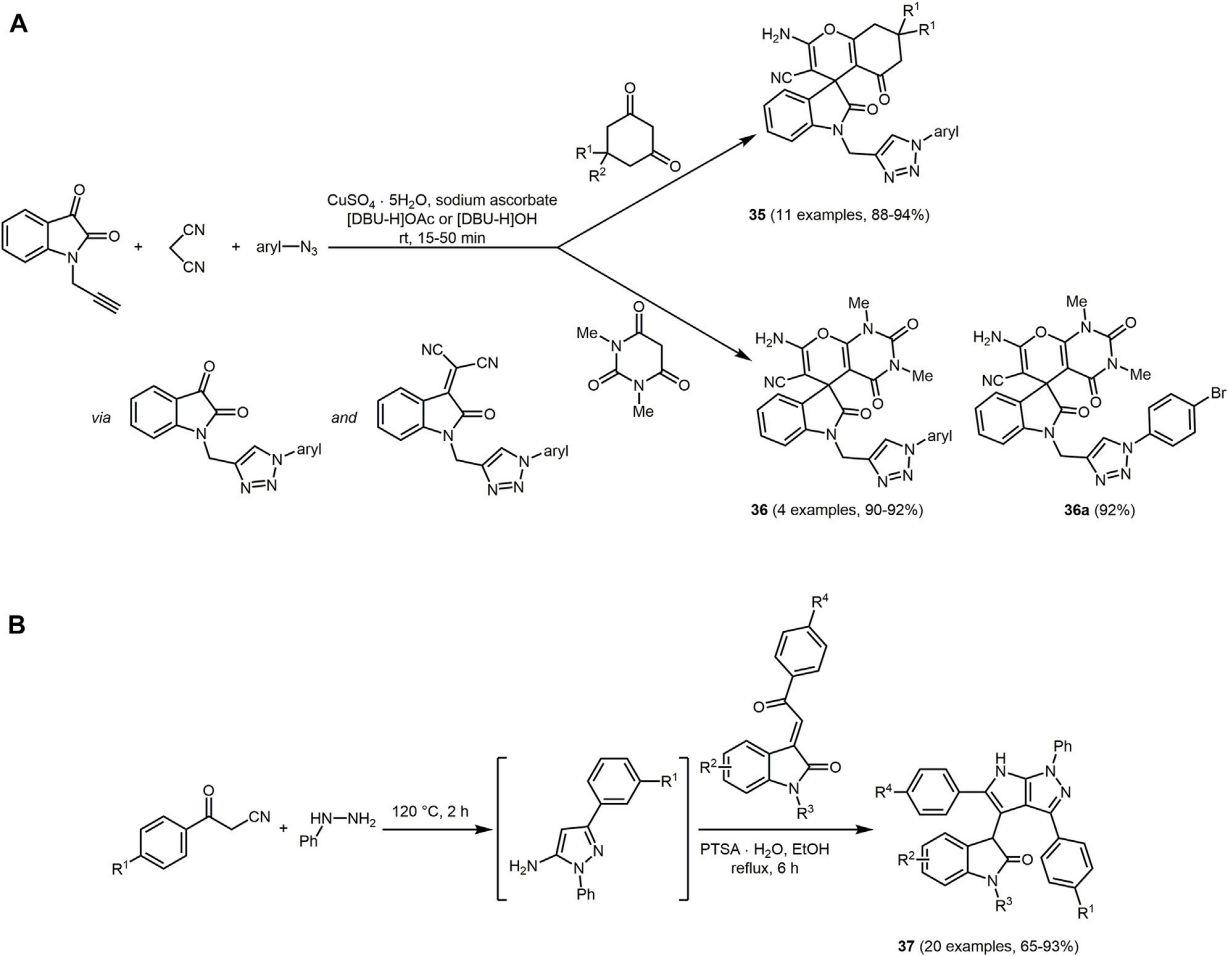
**(A)** Domino reaction for synthesis of triazolyl spirocyclic oxindoles **35** and **36** in the sense of the chromophore concept and the most potent fluorescent, antibacterial triazolyl spirocyclic oxindole **36a** (Singh et al., 2014) **(B)** Sequential one-pot synthesis of oxindole bearing pyrrolo[2,3-*c*]pyrazoles **37** (Nazeri et al., 2020).

As indoles, also oxindoles show considerable biological activity (Silva and Pinto, 2001; Peddibhotla, 2009; Kaur et al., 2016; Saraswat et al., 2016). Thus, fluorescent triazolylspirocyclic oxindole derivatives **35** and **36** containing pyran and 1,2,3-triazole moieties possess antimicrobial activity (Kerru et al., 2021). The synthesis proceeds *via* an one-pot domino reaction of 1-(prop-2-ynyl)indoline-2,3-dione, cyclic 1,3-diketones, malononitrile, and various arylazides in diazabicycloundecene-based ionic liquids ([DBU-H]OAc or [DBU-Bu]OH) under ultrasonic irradiation (Scheme 10A) (Singh et al., 2014). Mechanistically, a triazole intermediate is first formed by Cu-catalyzed alkyne-azide cycloaddition between 1-(prop-2-ynyl)indoline-2,3-dione and arylazide in the presence of *in situ* generated Cu(I). Knoevenagel condensation with malononitrile and subsequent Michael addition with the cyclic 1,3-dicarbonyl compound and cyclocondensation gives rise to the target structures in excellent yields.

All dyes **35** and **36** (*λ*_*max,abs*_ = 239–287 nm) exhibit fluorescence in methanol in a range from 366 to 417 nm with large Stokes shifts (Δ $\tilde{v}$ = 8,800–25,000 cm^−1^). In addition, compound **35a** is the strongest antibacterial agent against *Staphylococcus aureus* and *Bacillus subtilis* in this series.

Hybridizing several biologically active moieties in a single molecule *via* MCR also applies to chromophore **37**, a scaffold consisting of oxindole, pyrrole, and pyrazole (Jamwal et al., 2013; Karrouchi et al., 2018). Oxindole bearing pyrrolo[2,3-*c*]pyrazole **37** is prepared by an acid-promoted sequential three-component reaction between benzoylacetonitriles, phenylhydrazine, and 3-phenacylideneoxindoles in the sense of the chromophore approach (Scheme 10B) (Nazeri et al., 2020). In the first step, phenylhydrazine reacts with benzoylacetonitrile *via* cyclocondensation. This is followed by Michael addition with 3-phenacylideneoxindoles and concluded by cyclocondensation to give the desired products.

Absorption maxima of the violet fluorescent compounds **37** can be detected at around 220 and 355 nm. Electron-donating groups on the aromatic ring of the pyrrolo[2,3-*c*]pyrazoles display a slight redshift causing a decrease of the absorption and photoluminescence intensity.

Isoindoles are likewise important scaffolds of natural products and pharmaceuticals (Speck and Magauer, 2013; Kaur Bhatia, 2017; Csende and Porkoláb, 2018). The 3-substituted isoindolinone derivatives **38** and **39** have potential as cell sensors or drug carriers. The reaction proceeds as a Lewis acid-catalyzed, solid-phase MCR between chiral β-keto lactam, an aldehyde, an isocyanide, and a dienophile mediated by microwave energy. The synthesis of chromophore **38** employs immobilized aldehydes on a solid phase, whereas **39** uses immobilized dienophiles (Supplementary Material S7) (Massarano et al., 2020).

The 3-substituted isoindolinone derivatives **38** and **39** display significant fluorescence with large Stokes shifts (Δ $\tilde{v}$ = 3,900 nm). For example, dye **39a** shows an absorption maximum at 447 nm (*ε* = 5,300 L mol^−1^ cm^−1^) and an emission maximum of 542 nm (*Φ*_*F*_ = 0.3). Low cytotoxicity, water solubility, and rapid cell penetration of dyes **39a** and **39b** make them promising candidates as molecular probes for cell sensing and cell-penetrating transport agents.

**SCHEME 11:**
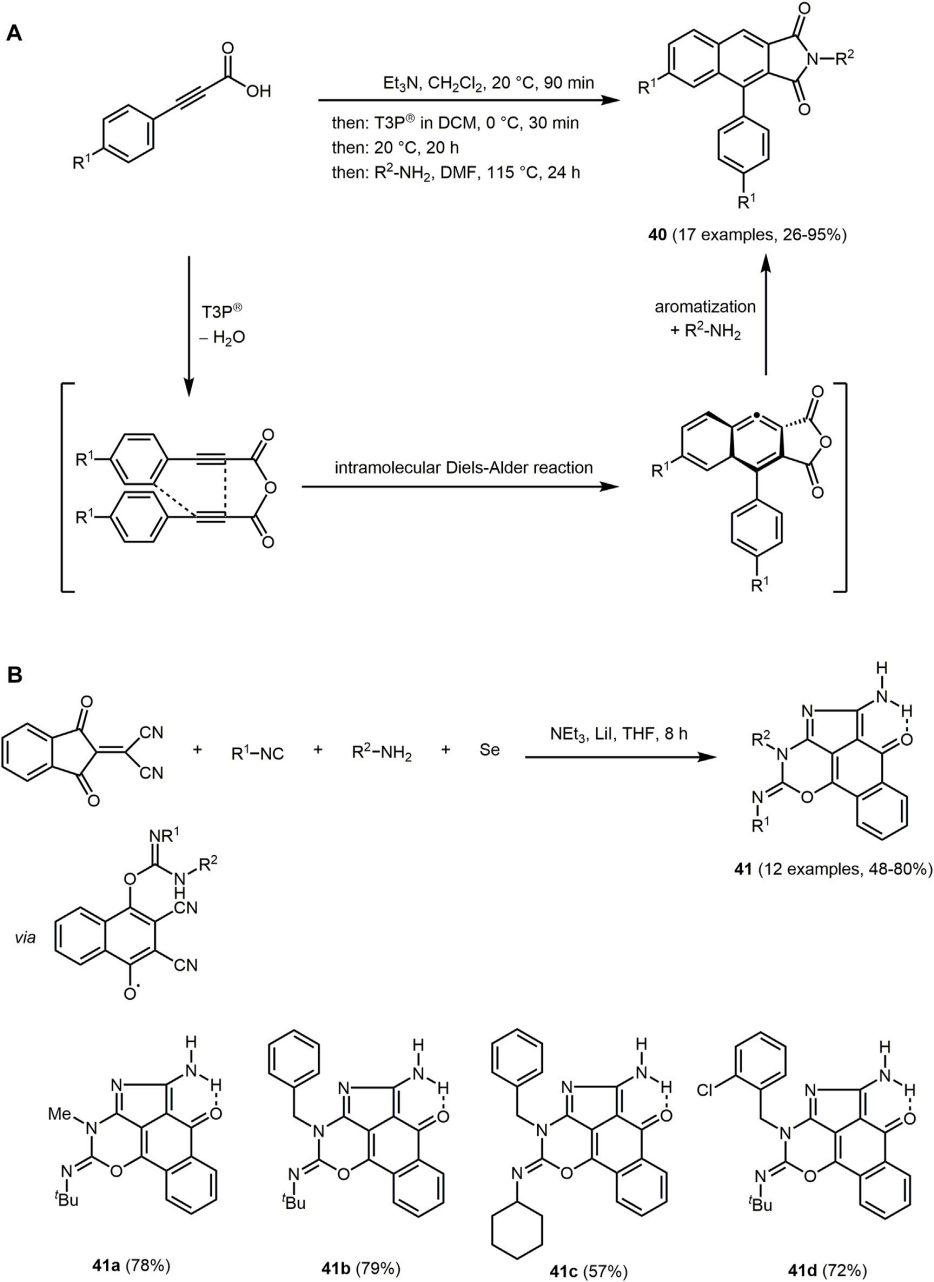
**(A)** Diversity-oriented one-pot process for the synthesis of 4-aryl-1*H*-benzo[*f*]isoindole-1,3(2*H*)-diones **40** (Denissen et al., 2017b). **(B)** Iodine catalyzed, selenium assisted sequential multicomponent synthesis of benzo-oxazino-isoindoles **41** and selected examples (Sedighian et al., 2021).

Blue-luminescent 4-aryl-1*H*-benzo[*f*]isoindole-1,3(2*H*)-diones **40** are formed by consecutive pseudo three-component synthesis of arylpropiolic acids and amines in the sense of the chromophore concept (Scheme 11A) (Denissen et al., 2017b). Arylpropiolic acids are *in situ* activated by *n*-propylphosphonic acid anhydride (T3P^®^) to give anhydrides. Intramolecular Diels–Alder reaction followed by aromatization forms the tricyclic anhydrides that are reacted with amines to give a substance library of tricyclic imide target compounds in 26% and 95% yield.

Intensive blue and greenish luminesce can by observed for several dyes **40** in solution at low concentration (Supplementary Material S8).

The UV/Vis spectra of the 4-aryl-1*H*-benzo[*f*]isoindole-1,3(2*H*)-diones **40** display two distinct absorption maxima in a range from 259 to 274 nm (ε ≈ 55,000 L mol^−1^ cm^−1^) and 359–379 nm (ε ≈ 3,500 L mol^−1^ cm^−1^). Electron-donating substituents R^1^ shift the absorption and emission maxima to longer wavelengths, accompanied by a significant increase in luminescence as seen for dye **40b** (Supplementary Material S8). Suppression of luminescence can also be caused by free rotation of the aryl substituent on the imide. Therefore, 4-aryl-1*H*-benzo[*f*]isoindole-1,3(2*H*)-diones **40** also possess AIE, where the intramolecular motion is suppressed upon aggregation. For instance, the fluorescence quantum yield of compound **40a** thereby increases more than eightfold in the solid state compared to emission in solution.

Another approach to access chromophores with an isoindole core proceeds *via* sequential MCR of the Knoevenagel adduct of ninhydrin and malononitrile, isocyanide, amine, and elemental selenium. The iodide-catalyzed process very likely involves a radical ring enlargement and furnishes luminescent benzo-oxazino-isoindole derivates **41** under mild reaction condition according to the chromophore concept (Scheme 11B) (Sedighian et al., 2021). Due to intramolecular hydrogen bonding, compounds **41** exhibits keto-enol tautomerism.

The dyes **41** are intensively luminescent. The absorption behavior in different protic and aprotic solvents shows minor changes in the absorption maxima (as detected at about 490 nm for derivative **41b**). In comparison, the emission spectrum displays a clear blueshift in methanol compared to the emission maxima in aprotic solvents. This shift might be attributed to hydrogen bonding of dyes **41** with hydrogen bond accepting solvents, lowering the ground state and increasing the excited state energies of the chromophores, resulting in a hypsochromic shift (Zakerhamidi and Sorkhabi, 2015).

Pyridoindolizine are particularly interesting to their biological properties as also indolizines (Sharma and Kumar, 2014; Venugopala et al., 2017). Pyrido[2,3-*b*]indolizine-10-carbonitriles **42** and **43** are formed by pseudo three-component reactions of *N*-(cyanomethyl)pyridinium salts with enaminones or vinamidinium perchlorates, respectively (Supplementary Material S9) (Sokolova et al., 2019). The proposed mechanism commences with a base-promoted dimerization of pyridinium salt. The elimination of pyridinium hydrochloride and aromatization leads to the formation of aminoindolizine, which condenses with 1,3-dielectrophiles to produce pyridoindolizines **42** or **43**.

Both pyridoindolizines exhibit strong green fluorescence with maxima in a range from 448 to 490 nm and with quantum yields of up to 0.81.

## 4 Six-membered heterocycles

### 4.1 Oxygen heterocycles

Chromenes can be regarded as benzo[*b*]pyrans. Depending on the positioning of the double bond in the pyran ring, two isomers arise, 2*H*-chromene and 4*H*-chromene. Coumarins are derived from 2*H*-chromenes, however, by oxidation of the 2-position to the oxidation state of an acid derivative, they are lactons. Coumarins absorb blue-green light and have found application as laser dyes (Winters et al., 1974; Abdel-Mottaleb et al., 1992; Yang et al., 2007), in chemosensors (Yao et al., 2009; Jung et al., 2011), in OLEDs (Puthumana and Damodaran, 2018), or in DSSCs (Hara et al., 2003; Wang et al., 2007; Seo et al., 2011). In addition, 4*H*-chromenes and coumarins are represented in a wide spectrum of pharmaceuticals (Montagner et al., 2008; Smyth et al., 2009; Thakur et al., 2015; Rawat and Verma, 2016; Ngabaza et al., 2017; Soni et al., 2019; Mishra et al., 2020). Due to their applications in the biomedical field, their preparation by sustainable methods for minimizing the usage and generation of toxic organic substances has become increasingly important. Therefore, several green methods have been reported for the synthesis of compounds with a coumarin or chromene core (Molnar et al., 2020; da Silveira Pinto et al., 2018; Ghosh et al., 2015; Mekheimer et al., 2010).

**SCHEME 12:**
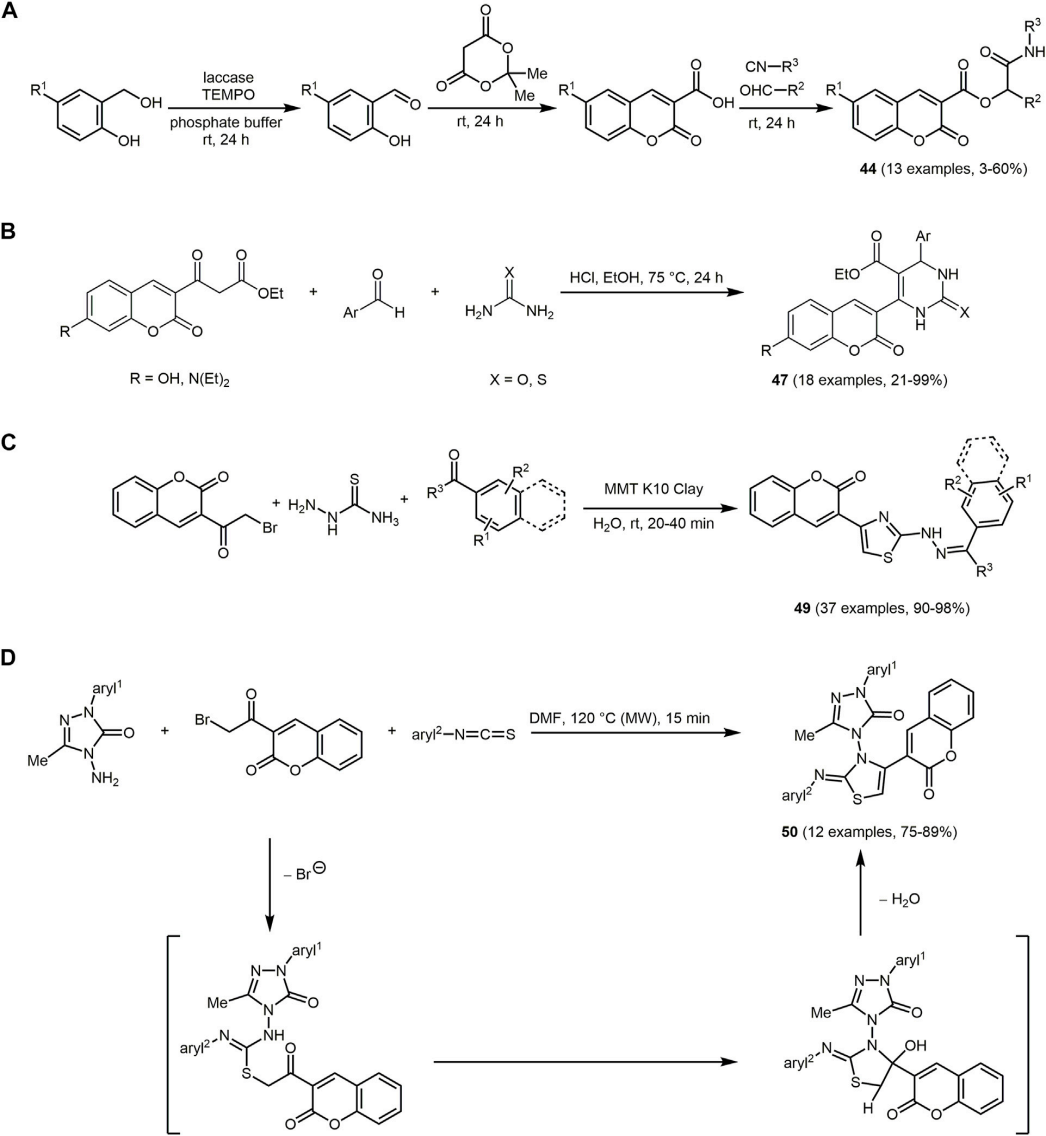
**(A)** Three-step one-pot synthesis of α-acyloxy carboxamides **44** (Paprocki et al., 2020). **(B)** Biginelli reaction to synthesize 3,4-dihydropyrimidin-2(1*H*)-one/thiones **47** in the sense of the scaffold concept (Vitório et al., 2015). **(C)** MMT K10 clay catalyzed three-component synthesis of diversified HTC derivatives **49** (Godugu et al., 2020). **(D)** Microwave-induced synthesis of coumarin-3-yl-thiazol-3-yl-1,2,4-triazolin-3-ones **50** (Shaikh et al., 2018).

An interesting sustainable approach is the three-step one-pot synthesis of α-acyloxy carboxamides **44** by Passerini reaction in sodium phosphate buffer solution as a medium. The three steps of the sequence starting from salicylic alcohol derivatives include a *Trametes versicolor* laccase catalyzed aerobical oxidation, aldol condensation and 6-O-glucose ester promoted Passerini MCR. By modification of alcohols, aldehydes, or isocyanides, α-acyloxy carboxamides containing a coumarin scaffold **44** are obtained in variable yields (Scheme 12A) (Paprocki et al., 2020). The synthesis can also be started with the third step employing coumarin-3-carboxylic acids as starting materials in the terminal Passerini reaction.

The obtained fluorescent α-acyloxy carboxamides **44** (*λ*_*max,em*_ = 415 nm) are well-suited as probes for enzyme activity profiling.

Another approach to produce coumarin-based dyes proceeds *via* condensation of 3-acetyl coumarin with acetyl chloride, an aromatic aldehyde, and acetonitrile to obtain blue light emitting fluorescent dyes named ‘Beta Fluors’ **45** (Supplementary Material S10) (Soumya et al., 2014). Phenyl boronic acid acts as a green catalyst for this reaction.

All chromophores **45** absorb with longest wavelength maxima between exhibit 303–346 nm and emit blue light from *λ*_*max,em*_ = 382–436 nm with large Stokes shifts.

The three-component reaction of 3-acetyl-7-(diethylamino)-2*H*chromen-2-one, 4-chlorobenzaldehyde, ethyl cyanoacetate provides D-π-A based coumarin-pyridone conjugate (CPC) **46** with a yield of 87% (Supplementary Material S11) (Manjunatha et al., 2022).

The absorption and emission spectra of the prepared CPC **46** are located in the range of 451–460 nm and 532–549 nm, respectively. Apart from the large Stokes shift, the molecule exhibits positive solvatochromic behavior. The peak at 490–510 nm in the solvatochromic absorption spectrum can be attributed to intramolecular charge transfer (ICT) from the donor (coumarin building block) to the acceptor (pyridone building block). Due to its special photophysical and electrochemical properties, this could be utilized as a fluorescent labeling agent not only for the visualization of latent fingerprints on various surfaces, but also as a detection of nitrite (NO_2_) *via* cyclic voltammetry (CV) and chronoamperometry (CA).

*Via* a Biginelli multicomponent reaction coumarin–dihydropyrimidinone dyads **47** starting from coumarin β-ketoester derivatives, various aldehydes and (thio)urea can be formed with yields ranging from 21% to 99% (Scheme 12B) (Vitório et al., 2015).

The synthesized chromophores **47** exhibit blue fluorescent properties, which can be slightly affected by the electronic properties of the aryl residue on the dihydropyrimidinone moiety, although there is no direct conjugation with the coumarin core. Based on the internal charge transfer process, the 3,4-dihydropyrimidin-2(1*H*)-one/thione, are promising candidates for novel chemical and biological probes in addition as useful p*H* indicators. As a selective Hg^2+^ chemosensor, chromenone-pyrazoles derivatives **48a** and **48b** can be applied, which are formed *via* a solvent-free one-pot sequence of salicylaldehyde derivative, 4-hydroxy-6-methyl-2*H*-pyran-2-one and hydrazine formed using SrFe_12_O_19_ as a catalyst. Through an one-pot multicomponent reaction using SrFe_12_O_19_ chromenone-pyrazole derivatives can be synthesized (Supplementary Material S12) (Ziarani et al., 2022).

The fluorescence properties of the prepared chromenone-pyrazole derivatives **48** are strongly determined by the residue on the pyrazole group. Thus, only the prepared non-aromatic introduced residues reveal fluorescence properties. The fluorescence emission of **48a** and **48b** in ethanol are at λ_ex_ = 348 nm and λ_ex_ = 300 nm, respectively.

Also, *via* simple work-up which even does not require purification *via* column chromatography, donor–acceptor type hydrazinyl thiazolyl coumarins (HTCs) **49** can be obtained (Scheme 12C). HTCs exhibits antioxidant, antimicrobial, and antibacterial activities (Kalluraya et al., 2001; Arshad et al., 2011; Osman et al., 2012; KhanYusufzai et al., 2017). Only a few MCRs to form HTCs are reported to date (Ibrar et al., 2016; Sujatha and Vedula, 2018). The water-mediated three-component reaction is catalyzed by a reusable, solid acid catalyst (montmorillonite (MMT) K10 clay) (Godugu et al., 2020). The wide variation of aromatic/hetero-aromatic aldehydes and aromatic ketones allows establishing a considerable substance library with excellent yields.

Most HTC derivatives **49** display bright fluorescence in chloroform (*λ*_*max,em*_ = 409–511 nm) with large Stokes shifts. Electron-donating groups in *para*/*meta* positions of the aromatic ring stemming from aldehydes/ketones result in bathochromic shifts. Furthermore, the electrochemical properties indicate that these compounds can be used as hole transporting materials.

The dyes **50** are chromophores that also contain coumarin and thiazole moieties and can be produced by an efficient microwave-induced, uncatalyzed one-pot reaction following the chromophore concept (Scheme 12D) (Shaikh et al., 2018). Components **50** also possess anticancer activity.

Fluorescence of compounds **50** is detected in the visible blue to green region. The chromophores **50** exhibit a bathochromic shift for both emission and absorption maxima with increasing solvent polarity. In non-protic solvents, the emission maxima is redshifted due to an intermolecular charge transfer process (ICT).

Further access to coumarin-based chromophores can be achieved by Ugi reaction of aromatic aldehydes, diamines, coumarin-3-carboxylic acid, and alkyl isocyanides producing coumarin-3-carboxamides containing lipophilic spacers **51** in the sense of the scaffold approach (Supplementary Material S13) (Balalaie et al., 2012). The Ugi 4CR adducts exhibit bright fluorescence at 544 nm in chloroform.

Chromenopyridinones derivatives are also bioactive (Fayed et al., 2021) and find application in the treatment of bronchial asthma (Ukawa et al., 1985) and they show anticancer activities (Bizarro et al., 2018). Furthermore, these heterocycles can be applied for the construction of chemosensors (Kozhevnikov et al., 2003). The one-pot three-component condensation of 4-hydroxycoumarins with ammonium acetate and 3-formylchromones gives highly substituted chromenopyridinone derivatives **51** in an environmentally benign solid-state melt reaction catalyzed by *L*-proline (Supplementary Material S14) (Paul and Lee, 2016). Mechanistically, the process can be rationalized by *L*-proline catalyzed Knoevenagel condensation of 4-hydroxycoumarin and 3-formylchromones *via* an iminium ion intermediate, followed by cyclocondensation with NH_4_OAc. The product **50** is thus obtained by a ring opening-ring closing sequence. The process follows the chromophore concept and produces the dyes in high yields (75%–93%).

Dye **52a** strongly emits in the range of 450–550 nm and the highest fluorescence intensity was detected in MeOH and the lowest in non-polar solvents. The methyl group in the 6-position of the coumarin ring accounts for the strong emission of component **52a**. In addition, electron-donating components as R^1^ and R^2^ groups are instrumental for ICT.

Further approaches to sustainable syntheses of chromenopyridine fluorophores following the chromophore concept are one-pot condensation sequences with salicylaldehyde derivatives, various malononitriles and selected O- or N-nucleophiles (Supplementary Material S15) (Zonouzi et al., 2013). This process can be applied to produce chromenopyridine derivatives such as dialkylamino-5*H*-chromeno[2,3-*d*]pyrimidin-2-yl-phenols **53**, dialkylamino-5*H*-chromeno[2,3-*d*]pyrimidin-2-yl-phenols **54** and 4-alkoxy-5*H*-chromeno[2,3-*d*]pyrimidines **55**.

All dye **53**–**55** show blue to green fluorescence with broad maxima upon excitation at 290 nm. Furthermore, the structures of the derivates **53a** and **54a** exhibit interesting, exchangeable, intramolecular H-bonding, which open an access to new phenol containing pharmacophores.

Compounds consisting of a phenazine and chromene core have found application in medicinal chemistry (Laursen and Nielsen, 2004). Blue emission is also observed from regioisomeric benzo[*a*]chromeno-phenazines **56** and **55** (Harichandran et al., 2018). The condensation of 1,2-phenylenediamine, 2-hydroxynaphthalene-1,4-dione, 2-hydroxy benzaldehydes, and 1,3-diketones catalyzed by IRA-400 Cl exclusively produces products **56**, whereas amberlite IR 120 H^+^ resin as a catalyst leads to a mixture of **56** and **57** (Supplementary Material S16). Advantageously, anion and cation exchange amberlite resins are both reusable. The intermediates are formed by the Knoevenagel condensation of phenylenediamine and 2-hydroxynaphthalene-1,4-dione, 2-hydroxy benzaldehydes and 1,3-diketones. The sequence finally concludes by Michael addition and condensation in the sense of the chromophore approach.

The absorption spectra of dyes **56** and **57** are characterized by two to three distinct maxima, with the longest wavelength absorptions in a range from 414 to 422 nm. The emission maxima of the chromophores **56** and **57** can be detected at wavelengths ranging from 462 to 498 nm and 450–468 nm, respectively. Most dyes **56** give fluorescence quantum yields ranging from less than 0.01 up to 0.10, as also the chromphores **57** reveal quantum yields of around or less than 0.1. The compounds **56a** and **57a** have shown to be suitable for the detection of Fe(III) and Cu(II) ions and can thus be used as chemical sensors.

An approach to increase sustainability of common reactions is the use of solvent-free syntheses to reduce organic solvents. As mentioned before, the multicomponent synthesis of fluorescent 4*H*-chromene derivatives **58** can be mediated by amberlite IRA-400 Cl resin, which functions both as the solvent and the reusable catalyst (Supplementary Material S17) (Harichandran et al., 2017). 2-Hydroxybenzaldehydes, 1,3-diketones, and nucleophiles (Nu) react involving by Knoevenagel condensation and subsequent Michael addition in excellent yields.

The UV/Vis spectra of the 4*H*-chromen dyes **58** display absorption maxima ranging from 252 to 394 nm and emission maxima in a range from 405 to 462 nm, blue fluorescence. Chromophore **58b** has been found to be the best fluorophore in the series. Compounds **58a** and **58b** are the only derivatives containing an electron donating amino group at the R^1^ position. These chromophores exhibit smaller Stokes shifts and higher fluorescence quantum yield (Δ $\tilde{\nu }$ ≈ 6,400 cm^−1^, *Φ*_*F*_ = 0.03–0.09) than the other congeners.

**SCHEME 13:**
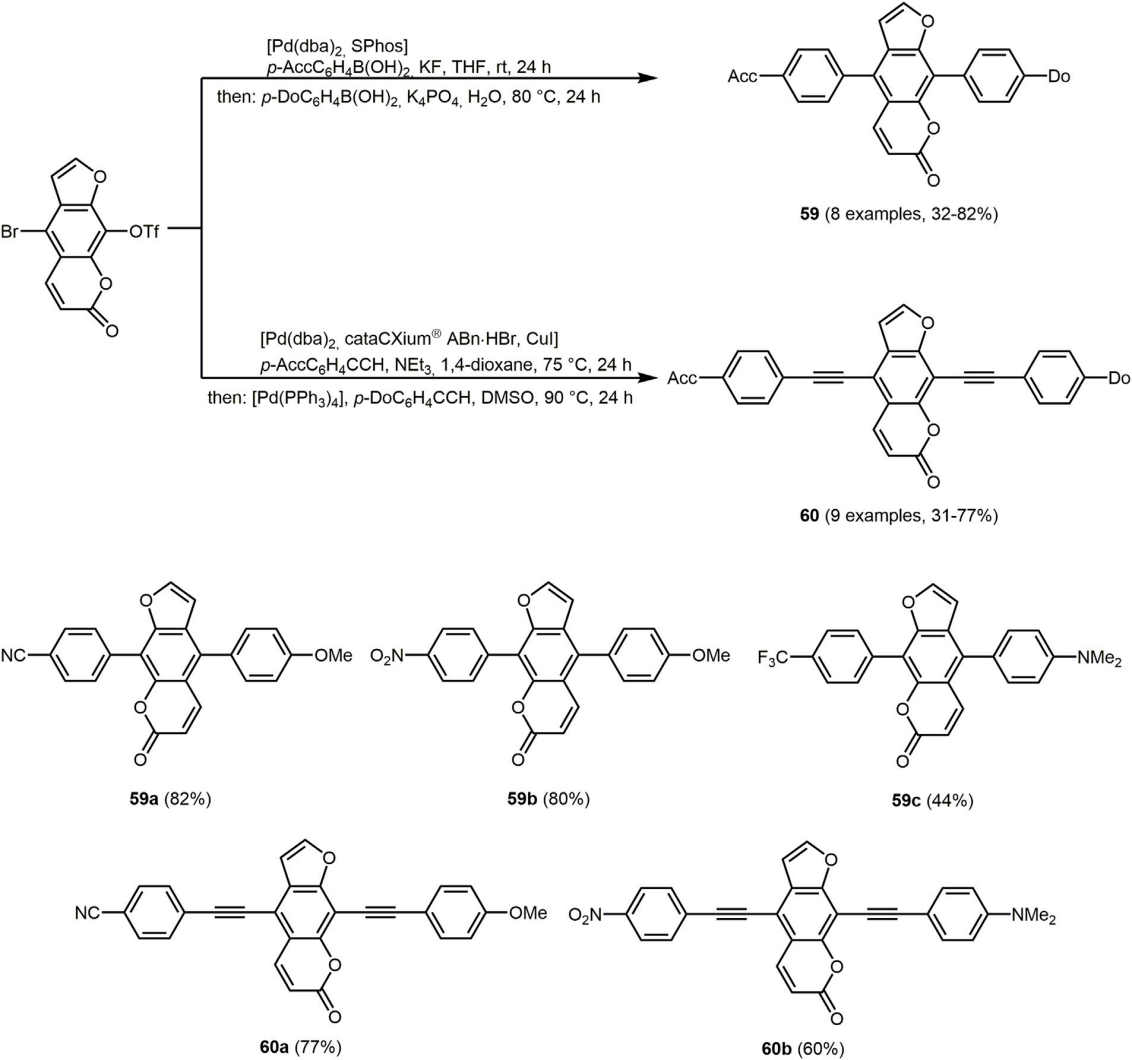
Consecutive three-component Suzuki−Suzuki and Sonogashira−Sonogashira synthesis of 8-donor-5-acceptor-substituted psoralens **59** and **60** in the sense of the scaffold concept and selected derivates (Geenen et al., 2020).

Furo[3,2-*g*]chromen-7-one, better known as psoralen, is a tricyclic donor-acceptor heterocycle consisting of an electron-rich furan and electron-poor α-pyrone fused to a central benzene core. Starting from bromo-triflato-functionalized psoralen donors and acceptors can be specifically coupled by sequentially Pd-catalyzed consecutive Suzuki-Suzuki and Sonogashira-Sonogashira multicomponent reactions to give donor-acceptor psoralens **59** and **60**, i.e. orthogonally oriented cruciform structures (Scheme 13) (Geenen et al., 2020). Cross-shaped molecules allow access to functional chromophores with interesting properties resulting from significant changes in the orientation of the transition dipole vector (Zucchero et al., 2010; Le et al., 2015; Xu et al., 2018).

Depending on the selection of the donor and acceptor components, the photophysical properties of the 8-donor-5-acceptor-substituted psoralens **59** and **60** can be fine-tuned. The optical properties of the donor-acceptor psoralens are characterized by large Stokes shifts, partially high fluorescence quantum yield in solution, and strong solid-state fluorescence. Of particular interest are observed solvatochromism, acidochromism, and aggregation-induced emission (Supplementary Material S18). In addition, the pronounced charge transfer character of the longest wavelength absorption band is confirmed experimentally and computationally.

**SCHEME 14:**
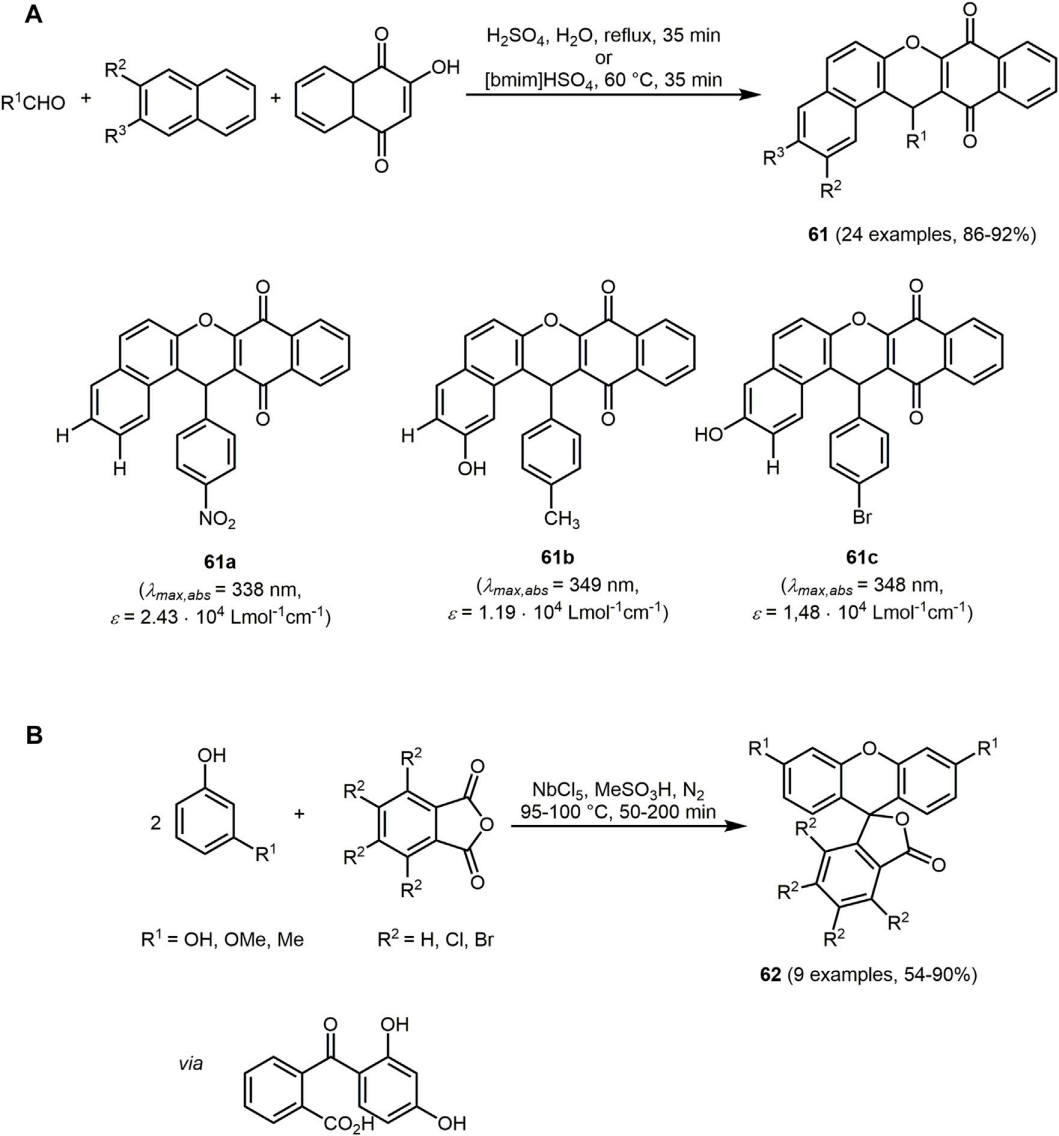
**(A)** Domino synthesis of 14-aryl-14*H*-dibenzo[*a,i*]xanthene-8,13-diones **61** and selected fluorophores (Khurana et al., 2012). **(B)** One-pot synthesis of fluoresceins **62** using NbCl_5_ as a catalyst (Sacoman Torquato da Silva et al., 2017).

Fluorescence of xanthene-containing chromophores **61** can also be observed in natural products (Bhowmik and Ganguly, 2005). Due to their photophysical properties, they are mainly applied in dyes and can function for instance as p*H*-sensitive fluorescent materials for visualization of biomolecules (Knight and Stephens, 1989; Shabir et al., 2018). In addition, these compounds have various biologically active properties, such as anticancer, antibacterial and antioxidant (Kidwai et al., 2005; Giri et al., 2010; Raschip et al., 2020). The antioxidant activity of 14-aryl-14*H*-dibenzo[*a,i*]xanthene-8,13-diones **61** containing a hydroxy group at R^2^ or R^3^ was tested using the 2,2-diphenyl-1-picrylhydrazyl radical scavenging assay (Khurana et al., 2012). The electron-donating groups on the aldehyde (R^1^), as dyes **61b** and **61c**, enhance this antioxidant characteristics. Possess antioxidant features. 14-Aryl-14*H*-dibenzo[*a,i*]xanthene-8,13-diones **61** can be prepared *via* condensation of aldehydes, 2-hydroxynaphthalene-1,4-diones, and 2-naphthol/2,7-dihydroxynaphthalenes/2,6-dihydroxynaphthalenes catalyzed either by H_2_SO_4_ or ionic liquid 1-butyl-3-methylimidazolium hydrogen sulfate ([bmim]HSO_4_) (Scheme 14A). For xanthenes in general, many green approaches have been reported to involve environmentally benign catalysts such as ionic liquids (Burange et al., 2021). Another advantage of the domino MCR following the chromophore approach is the simple purification by crystallization in ethanol. The synthesized compounds **61** exhibit absorption bands with maxima in the range from 333 to 355 nm with extinction coefficients between 10,800 and 27,500 L mol^−1^ cm^−1^.

Fluoresceins can also be categorized as xanthene dyes. These are characterized by interesting photophysical properties such as high molar absorptivity, high fluorescence quantum yields, and high photostability. Typically, fluoresceins absorb in the range from 400 to 700 nm (Sjöback et al., 1995; Song et al., 1998). Depending on the p*H* value, fluorescein is present as an anion, cation, or in its neutral form, which influences the photophysical properties (Martin and Lindqvist, 1975; Diehl and Markuszewski, 1989). This p*H* dependency can be exploited for the application as an indicator (Kim et al., 2011). The UV/Vis spectra of the fluorescein derivatives **62** were measured in NaOH so that the fluorescein derivatives are present in the dianionic form (p*H* > 8) (Sacoman Torquato da Silva et al., 2017). The dianionic form shows longest wavelength absorption and emission maxima and highest fluorescence quantum yields due to its conjugated system. The absorption (*λ*_*max,abs*_ = 476–512 nm) and emission (*λ*_*max,em*_ = 515–525 nm) of dyes **62** lie in the typical range of fluorescein and high fluorescence quantum yields are realized (*Φ*_*F*_ = 0.60–0.93). Derivatives containing halogens show a bathochromic shift in emission and absorption behavior as well as a decrease in fluorescence intensity. The fluorescein dye derivatives can be formed by a pseudo three-component reaction of phenol and phthalic anhydride derivatives in the sense of the chromophore approach (Scheme 14B). This MCR can be catalyzed by the electrophilic NbCl_5_. The reaction proceeds in two steps *via* twofold Friedel-Crafts reaction and subsequent carbonyl addition. In addition, the interesting photophysical of fluoresceins **62** make them suitable for application in DSSC.

### 4.2 Nitrogen heterocycles

Pyridine (azine) is found in many bioactive natural products, such as vitamin B_6_ (Hill et al., 1996) or nicotinamide adenine dinucleotide, which is a pivotal coenzyme in the metabolism (Noctor et al., 2006). Furthermore, pyridine is an important synthetic building block for pharmaceuticals (Chang et al., 2005; Reddy et al., 2006; Altaf et al., 2015; Nagham Mahmood Aljamali, 2021). Pyridine is an excellent moiety for the assembly of fluorescent compounds due to its strong electron withdrawing properties, good rigid structure, and strong coordination ability (Xie et al., 2021). Numerous chemosensors with a pyridine core are reported (Hirano et al., 2000; Jiang and Guo, 2004; Machado et al., 2014; Ma et al., 2017). For instance, a fluorescent chemosensors can be used for the detection of Fe(III) and Hg(II) ions based on an ON-OFF mechanism. The fluorescent 2-amino-6-methyl-4-phenyl-nicotinonitrile **63** is synthesized *via* multicomponent condensation following the chromophore concept (Supplementary Material S19) (Koner et al., 2012).

The coordination of Fe(III) ions and 2-aminopyridine-based compound **63** results in an increase of the absorption intensity of both bands *λ*_*max,abs*_ = 246 and 335 nm. In contrast, Hg(II) ions lead to a decrease in absorption intensity with a slight bathochromic shift. In the presence of Fe(III) and Hg(II) ions, the fluorescence spectrum shows quenching of emission intensity by 81% and 55%, respectively. Quenching is very likely caused by the paramagnetic nature of the Fe(III) ions and by the heavy metal ion effect for Hg(II). Due to the small spectral shift upon emission decrease, photo-induced electron transfer (PET) is a plausible mechanism. It is noteworthy that selective quenching occurs only for Fe(III) and Hg(II) ions, which makes the chromophore a suitable chemosensor for these two metal ions. Iron is involved in many biological processes and its selective detection can be a valuable tool for biological studies (Sigel and Sigel, 1998; Que et al., 2008).

**SCHEME 15:**
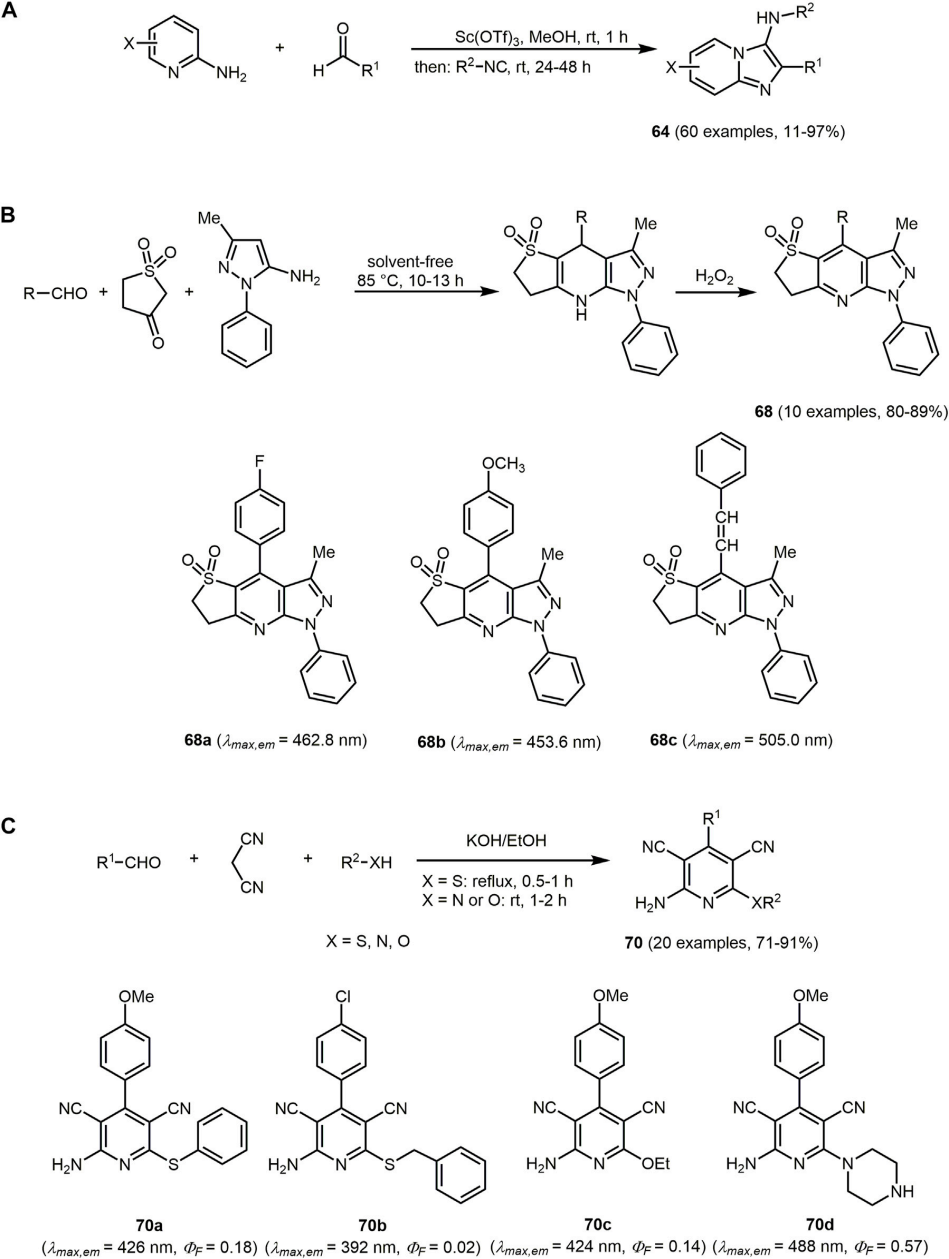
**(A)** Groebke-Blackburn-Bienaymé synthesis of fluorescent α,β-substituted imidazo[1,2-*a*]pyridines **64** *via* the chromophore approach (Burchak et al., 2011). **(B)** Solvent-free MCR synthesis of pyrazole[3,4-*b*]thieno[2,3-*e*]pyridine derivatives **68** in the sense of the chromophore approach and selected fluorophores (Yao et al., 2014). **(C)** Pseudo four-component synthesis of functionalized 2-amino pyridine dyes **70** and selected derivates (Khan et al., 2012b).

The Groebke-Blackburn-Bienaymé (GBB) three-component reaction of heterocyclic amidines, aldehydes, and isocyanides furnishes α,β-substituted imidazo[1,2-*a*]pyridines **64** (Scheme 15A) (Burchak et al., 2011), an important source of bioimaging probes due to their pharmacophore. Imidazo[1,2-*a*]pyridines have been shown to possess antiviral (Gueiffier et al., 1998), antiulcer (Starrett et al., 1989), antipsychotic (Marcinkowska et al., 2016), and antidiabetic activities (Kercher et al., 2007).

In general, the absorption band of imidazo[1,2-*a*]pyridine is located near 280 nm and an efficient fluorescence band is observed at 370 nm (Stasyuk et al., 2012). The emission properties of the dyes **64** are mainly affected by the amidine and aldehyde building blocks. Thus, the products starting from methyl 2-aminoisonicotinate, methyl 2-aminoisonicotinate, pyrimidin-2-amine, and pyrazin-2-amine fluoresce intensively in solution. All the mentioned imidazo[1,2-*a*]pyridines exhibit intrinsic fluorescence with a broad range of colors and they are also promising for use in chemosensors. The fluorescence mainly depends on the electron-donating effect and the degree of conjugation of aldehyde moiety (Supplementary Material S20).

The GBB synthesis of imidazo[1,2-*a*]pyridines **65** proceeds under mild conditions and short reaction times giving good yields after simple non-aqueous workup (Supplementary Material S21) (Khan et al., 2012a; Khan et al., 2012b). In this variant of the three-component condensation, bromodimethylsulfonium bromide (BDMS) serves as the catalyst. Thereby, even sterically demanding amidines can be transformed with good yields. The released water from condensation reacts with BDMS to liberate HBr which protonates the imine. Mechanistically, the key step of the formation of imidazo[1,2-*a*]pyridines **65** is a [4 + 1] cycloaddition followed an aromatizing 1,3-H shift.

Absorption maxima of the fluorescent compounds **65** can be detected at around 250 and around 335 nm. A chlorine substituent at C_2_-position of the imidazo[1,2-*a*]pyridine results in a slight redshift of the absorption maxima. A 2,4-methoxy substituent on the aromatic ring shifts the emission maximum to a longer wavelength (*λ*_*max,em*_ = 470 nm).

To synthesize tosylmethyl isocyanide (TOSMIC) fused imidazo[1,2-*a*]pyridine **66**, the GBB reaction is carried out with 2-amino pyridine, TOSMIC and a wide variation of aldehydes in the sense of the chromophore concept (Supplementary Material S22) (Shukla et al., 2022).

The obtained imidazo[1,2-*a*]pyridine derivatives **66** display a blue color under the UV lamp. Based on the aldehyde component, the fluorescence properties can be modified.

The pseudo three-component reaction of mucobromic acid and two molecules of a series of 2-substituted benzimidazoles provides benzo[4,5]imidazo[1,2-*a*]pyridine derivates **67** in moderate to good yields. Mechanistically, a nucleophilic substitution of benzimidazoles with mucobromic acid occurs in the presence of the base potassium carbonate. After decarboxylation, Michael addition of another benzimidazole is performed. With the aid of the base, a dehydrohalogenation reaction proceeds and the desired product is obtained by cyclization (Supplementary Material S23) (Yang et al., 2022). *Via* Michael addition, other NH-containing heterocyclic nucleophiles can also be introduced to the system.

The introduced heterocyclic rings in C_1_ position of benzo[4,5]imidazo[1,2-*a*]pyridine **67** lead to the cancellation of the coplanarity of the whole molecule and hinder the strong intermolecular π-π interaction and the tight π-π stacking between the neighboring molecules. These electrostatic interactions are able to constrain intramolecular motion in the solid state, causing an enhancement of fluorescence in the aggregation state. **67a** could be used to detect picric acid, a nitroaromatic explosive.

Pyrazole[3,4-*b*]thieno[2,3-*e*]pyridines **68** contain pyrazoles and thienyl moieties fused to a central pyridine core and can be accessed by Hantzsch dihydropyridine synthesis followed by a solvent-free oxidative aromatization with H_2_O_2_ (Scheme 15B) (Yao et al., 2014). The aromatic substituent of the aldehyde can be electron-withdrawing or electron-releasing and it influences the emission behavior. The dyes **68** feature a blue to green fluorescent donor-π-conjugated acceptor system with emission maxima in a range from 430 to 505 nm.

Pyrene moieties can be placed to pyridines by the chromogenic one-pot pseudo four-component pyridine synthesis starting from 1-acetylpyrene, arylaldehydes, and ammonium acetate in acetic acid to form 4-aryl-2,6-di (pyren-1-yl)pyridines **69** in good yields ranging from 65% to 86% (Supplementary Material S24) (Asaadi et al., 2019).

The dyes fluoresce in a range of 434–464 nm with quantum yields of 0.10–0.17. The fluorescence is increased by electron-donating groups (**69c**) due to the conjugation of the system, where electron-withdrawing groups (**69d**) diminish the fluorescence.

Highly substituted pyridines can be formed *via* potassium hydroxide catalyzed pseudo four-component reaction of aldehydes, malononitrile and nucleophiles, such as thiols, alcohols or amines in the sense of the chromophore concept (Scheme 15C) (Khan et al., 2012b). Mechanistically, one molecule of malononitrile reacts *via* a Knoevenagel condensation with aldehyde and the other malononitrile undergoes a Michael addition with the previously formed electrophile followed by a concomitant nucleophilic addition to the cyano-substituent of the adduct. The functionalized 2-amino pyridines **70** are formed *via* cyclization and oxidative aromatization.

The UV/Vis spectra of 2-amino-3,5-dicarbonitrile-6-thio-pyridines **70** display absorption maxima in a range from 329 to 356 nm and emission maxima in a range from 386 to 451 nm. The highest quantum yield in comparison to other thio-pyridine derivatives (*Φ*_*F*_ = 0.00–0.05) is detected for compound **70a** (*Φ*_*F*_ = 0.18), which possesses a strongly electron-donating group at the C_4_ aryl substituent of the pyridine ring. The amino and oxo pyridine derivatives exhibit quantum yields of 0.27–0.57 and 0.10 to 0.14, respectively. The chromophores are potential candidates as new fluorescent probes or luminescence materials. Furthermore, it was reported that when sterically hindered aldehydes are used not the expected thio-pyridine derivatives but the corresponding 1,4-dihydropyridines (DHPs) were obtained.

Pyridine is the oxidation (dehydrogenation) product of 1,4-dihydropyridine (1,4-DHP), which also occurs in coenzymes nicotinamide adenine dinucleotide (NADH) and nicotinamide adenine dinucleotide phosphate (NADPH). Synthetic derivatives of 1,4-DHP are ubiquitous and cover a wide spectrum of biological activities, including vasodilators (Schachinger et al., 2001; Safak and Simsek, 2006), antibacterials (Huang et al., 2009; Sellamuthu et al., 2017) and antioxidatives. As a drug, DHP is used as a calcium channel blocker in the treatment of hypertension (Young, 1984; Edraki et al., 2009; Poondra et al., 2013). 1,4-DHPs, which are available by Hantzsch synthesis, are also used as dyads in photoinduced electron-transfer systems (Fasani et al., 2006; Jimenez et al., 2009; Al-Awadi et al., 2012).

**SCHEME 16:**
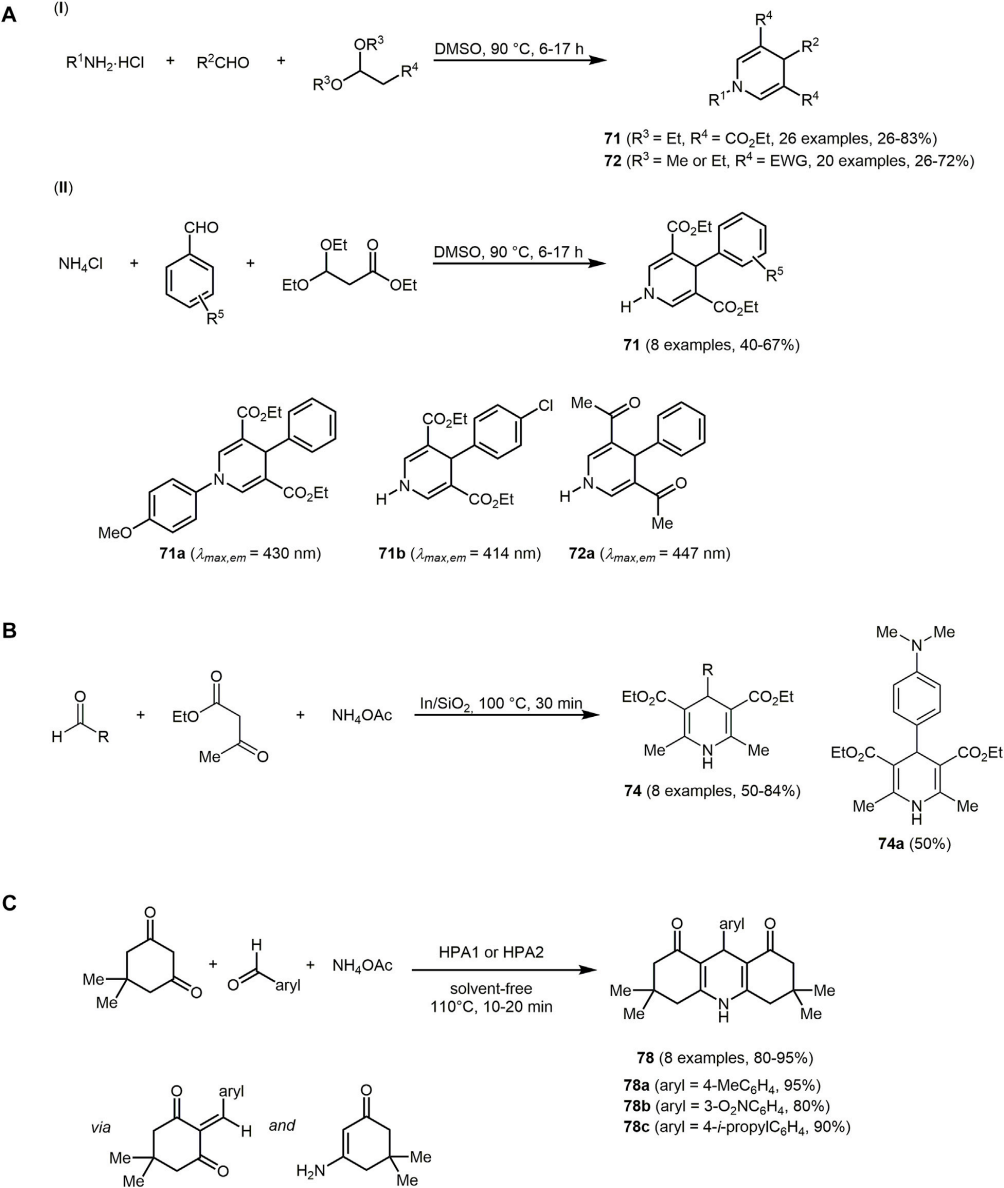
**(A)** Catalyst-free pseudo four-component synthesis of 3,4,5-substituted 1,4-dihydropyridines **71** and **72** and selected fluorophores (Sueki et al., 2014). **(B)** Synthesis of photoactiv Hantzsch 1,4-DHPs **74** as well as one selected derivate **74a** (Affeldt et al., 2012). **(C)** Pseudo four-component synthesis of 1,8-dioxodecahydroacridines **78** with the green catalysts HPA1 or HPA2 (Baradaran-Sirjani et al., 2018).

1,4-Dihydropyridines **71** and **72** are formed *via* catalyst-free MCR of amine hydrochloride salts or ammonium chloride, aldehydes, and acetals (Scheme 16A) (Sueki et al., 2014). Ammonium chloride and 3,3-diethoxypropionate activated by protons react to give an imine intermediate that tautomerizes to the enamine. This enamine intermediate forms with an aldehyde upon elimination of ethanol an α,β-unsaturated imine. Subsequently, the enamine intermediate reacts with the α,β-unsaturated imine *via* Michael anellation to form tetrahydropyridine. Upon elimination of ammonia 1,4-DHP is generated. Based on the two approaches A and B, a wide range of various 3,4,5-trisubstituted 1,4-DHPs with ethoxycarbonyl groups at the 3- and 5-positions **71** can be formed (Scheme 16A I). Further modification can be achieved by changing the ethoxycarbonylgroups to other electron-withdrawing groups (EWGs) and the corresponding 3,4,5-substituted 1,4-DHPs **71** are obtained in moderate to good yields in the sense of the chromophore concept (Scheme 16A II).

The MCR products **71** and **72** exhibit fluorescence in the range from 403 to 542 nm and quantum yields up to 0.94. 1,4-DHPs with extended π-conjugation exhibit emission maxima at longer wavelengths. Photophysical properties such as the fluorescence wavelength and the quantum yield can be varied by modifying the substituents of the 1,4-DHPs.

A green approach to 1,4-DHPs is presented by the one-pot five-component synthesis of 1,2,3-triazole-linked pentasubstituted 1,4-DHPs **73** under ultrasonic irradiation at room temperature or under MW irradiation with PEG-400 as a medium (Supplementary Material S25) (Singh et al., 2013). PEG-400 is an inexpensive, biologically compatible, non-toxic and recyclable solvent. Initially, a CuAAC occurs between aryl azide and the propargylated benzaldehyde derivative, forming a 1,2,3-triazole derivative. Subsequently, the disubstituted 1,2,3-triazole-linked DHPs are produced by the Hantzsch condensation involving Knoevenagel condensation and Michael addition.

All chromophores **73** display three distinct absorption maxima in the UV/Vis spectra, the first around *λ*_*max,abs*_ = 230 nm, the second around 255 nm, and the third around 365 nm. Also, the 1,2,3-triazole-linked pentasubstituted 1,4-DHPs show strong fluorescence in solution with emission maxima between 439 and 451 nm. It is worth noting that the substituent on the phenyl ring has only a minor influence on the fluorescence properties. Moreover, the compounds **73** are proven to possess antibacterial, antifungal and antioxidant properties.

The In/SiO_2_ catalyzed Hantzsch reaction of various substituted aryl aldehydes provides access to blue-green fluorescent 1,4-DHPs **74** (Scheme 16B) (Affeldt et al., 2012)

The UV/Vis spectra of **74** exhibit the absorption maxima at around 350 nm. The emission maxima can be detected in a range of 402–516 nm with large Stokes shifts ranging from 4,200 to 11,000 cm^−1^. In particular, for derivatives **74a** the largest Stokes shift is measured, due to intramolecular charge transfer mechanism in the excited state from the dimethylaniline to the dihydropyridine chromophores.

Similarly, hydroxyphenylbenzoxazole, a heterocycle with interesting photopyhsical properties such as large Stokes shift, ESIPT or dual fluorescence emission, can be introduced as an aldehyde component in the Hantzsch reaction (Rodembusch et al., 2007; Grando et al., 2009). The addition of 5,5-dimethylcyclohexane-1,3-dione and/or ethyl acetoacetate provide 1,4-DHPs **75**–**77** (Supplementary Material S26) (Affeldt et al., 2014).

The three synthesized 1,4-DHPS **75**, **76** and **77** display absorption in the UV region and emission in the blue-green region. The photophysical study reveals that the hydroxyphenylbenzoxazole and DHP fluorophores in the hydroxyphenylbenzoxazole-DHP structure behave independently after excitation. In addition, ESIPT emission can be observed.

The pseudo four-component reaction for the synthesis of polyfunctionalized derivatives of 1,4-DHP and 1,8-dioxodecahydroacridines can be efficiently catalyzed by two Preyssler heteropolyacids, H_14_[NaP_5_W_29_MoO_110_] (HPA1) and H_14_[NaP_5_W_30_O_110_] (HPA2), under solvent-free conditions (Baradaran-Sirjani et al., 2018). The reaction is catalyzed by both polyoxometalate anions and cations. The cations activate the carbonyl groups in aromatic aldehydes and dimedones due to their Lewis acidic nature and the anions abstract the α-proton of the dimedone furnishing 1,8-dioxodecahydroacridines **78** in excellent yields based on the chromophore concept (Scheme 16C).

The emission spectra of **78c** recorded in different solvents and at different temperatures exhibit a maximum at around 520 nm under all conditions.

Based on a pseudo five-component reaction of cyclopentanone, two molecules of aromatic aldehyde, *N*-pyridinium substituted ortho-hydroxyaryl methyl ketone and ammonium acetate 2-(ortho-hydroxyaryl)cyclopenta[*b*]pyridines **79** and **80** are synthesized with yields ranging from 19% to 87% (Supplementary Material S27) (Batalin et al., 2021). For the aromatic aldehyde either benzaldehyde or up to 3 methoxy groups with various substitution patterns on the aromatic ring were furnished, which influenced the yields and the amount of pyridium salt added. The reaction proceeds *via* a modified Kröhnke reaction (Yan et al., 2007), in which the first step is an aldol condensation between the cyclopentanone and the aromatic aldehyde in the presence of ammonium acetate. The resulting E-cross conjugated dienone undergoes a Michael reaction with the pyridime salt and forms the intermediate illustrated in the Supplementary Material S27, which after elimination of the pyridinium cation forms the unsaturated 1,5-diketone. In the final step, the nucleophilic addition of ammonia leads to the compounds **79** or **80**.

The derivatives exhibit interesting fluorescence properties such as excited-state intramolecular proton transfer (ESIPT) in solution. Excitation maxima are measured at 374 nm and emission maxima at 434 nm under neutral conditions. In acidic conditions, excitation maxima and emission maxima for compounds **79** and **80** are found at 332–476 nm and 484–606 nm, respectively. An increase in acid concentration results in either an increase in fluorescence intensity or quenching of fluorescence in the case of newly formed fluorophores in solution, according to the specific derivative. In the crystalline state, double emission due to ESIPT was observed with fluorescence maxima of the enolimine tautomers ranging from 414 to 426 nm. The fluorescence maxima of the keto amine tautomers are within the region of 594–624 nm with high Stokes shifts.

**SCHEME 17:**
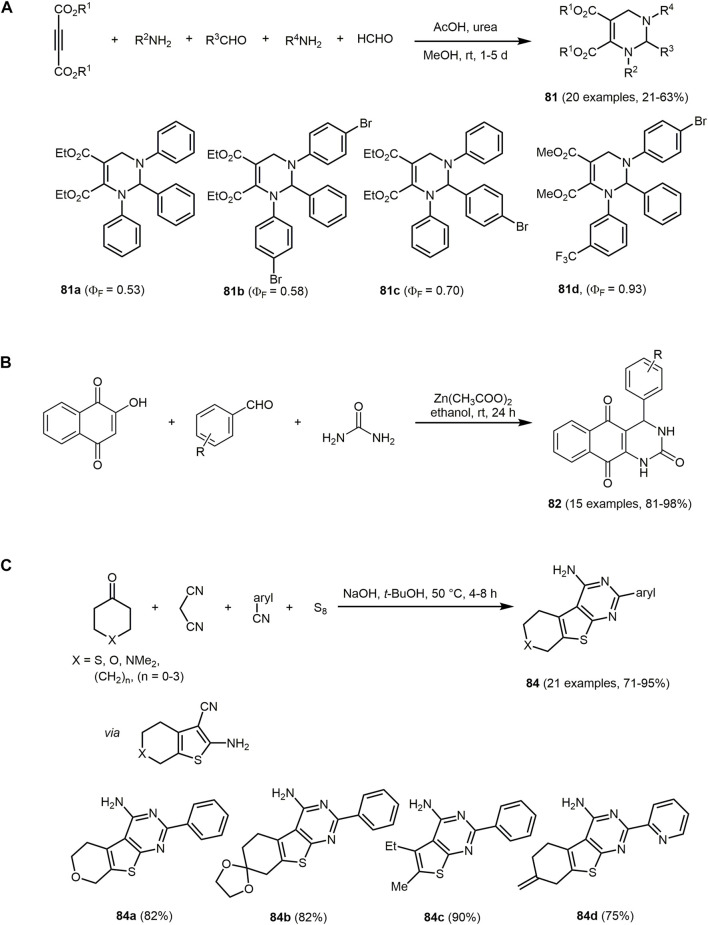
**(A)** Five-component reaction of tetrahydropyrimidines **81** and selective dyes with quantum yields above 0.50 (Zhu et al., 2013). **(B)** Synthesis of disperse dyes with a dihydropyrimidinone scaffold **82** *via* one-pot multicomponent reaction (Patel et al., 2022). **(C)** Modified Gewald synthesis of 2-arylthieno[2,3-*d*]pyrimidin-4-amines **84** and selected examples (Abaee et al., 2017).

Pyrimidine derivatives display biological and pharmacological characteristics, including antitumor (Atwal et al., 1989; El-Subbagh et al., 2000), antibacterial (Cieplik et al., 2011; Selvam et al., 2015) and anticancer (Joshi et al., 2016; Mangal and Jangid, 2016) properties. Pyrimidine nucleosides as well as alkaloids and antibiotics have been isolated from natural sources (Lagoja, 2005). Access to different pyrimidine derivatives is possible for example *via* Biginelli MCR of aldehydes, urea and methylene active compounds under dielectric heating (Dabiri et al., 2007; Tu et al., 2009). In addition, some chromophores with a pyrimidine core show interesting photophysical properties. C_6_ unsubstituted tetrahydropyrimidines **81** exhibit aggregation-induced emission enhancement (AIEE) and size-independent emission (SIE) characteristics (Zhu et al., 2013). They emit blue or green fluorescence in aggregates (*λ*_*max,em*_ = 399–491 nm) with fluorescence quantum yields of up to 0.93. For derivatives with aromatic substituents in the R^2^ and R^4^ position, a strong emission in aggregates can be observed compared to substituents with alkyl groups. The derivative **81a** shows SIE characteristics since emission maximum of suspension particles, powder, film and crystals are identical at and appear at 434 and 484 nm, respectively. The synthesis is performed under mild conditions and catalyzed by the organocatalyst urea. The five-component reaction allows the construction of a substance library in which all starting materials except aldehyde are varied giving yields between 21% and 63% in the sense of the chromophore approach (Scheme 17A). The reaction contains four elementary steps starting with an aminovinylation, followed by aza-ene-type reaction, nucleophilic addition, and cyclization.

Recently, *via* Biginelli reaction 3,4-dihydropyrimidin-2-(1*H*)-one derivatives **82** with excellent yields are formed. The reaction of Lawson, various aromatic aldehydes and urea is catalyzed using zinc acetate (Scheme 17B) (Patel et al., 2022).

The 3,4-dihydropyrimidin-2-(1*H*)-ones derivative **82** display well-defined color regions such as red, orange and yellow with good intensity. The UV-visible absorption spectra in DMF exhibit absorption maxima in the region from 399 to 493 nm.

Further dihyropyrimidines derivatives can also be prepared *via* the Biginelli reaction. By employing fluorescent β-ketoamides, hybrid fluorescent 3,4-dihydropyrimidine-2-(thi)ones **83** can be obtained (Supplementary Material S28) (de Souza et al., 2020).

The synthesized dihydropyrimidines derivatives **83** exhibit fluorescence in solution with large Stokes shifts, due to a proton transfer process. Moreover, fluorophores reveal double fluorescence emission. The emission at short wavelengths is attributed to the excited enol forms and at longer wavelengths to the tautomeric species, which can be associated with the intramolecular excited state proton transfer process (ESIPT). Further studies confirm a cytotoxic activity of the compounds and their potential application as fluorescence probes.

Pyrimidines can also be employed as metal ion sensors. For example, an acetonitrile solution of 2-arylthieno[2,3-*d*]pyrimidyl-4-amine **84d** changes color from colorless to yellow at higher concentrations of Pd(II) ions, which is visible to the naked eye. The absorption spectrum reveals a decrease of the three bands of the dye (*λ*_*max,abs*_ = 221, 244 and 332 nm) on expense of a new band with an isosbestic point at 362 nm in presence Pd(II) ions. The synthesis of 2-arylthieno[2,3-*d*]pyrimidin-4-amines **84** proceeds *via* a modified Gewald reaction (Gewald et al., 1966) with four components, where the additional component exploits the reactivity of the two neighboring functional groups of the classical three-component product. The starting materials are various α-methylene bearing ketones, malononitrile, aryl or heteroarylnitrile derivatives and elemental sulfur, which form functionalized products in good to excellent yields in the sense of the chromophore approach (Scheme 17C) (Abaee et al., 2017).

Another chemosensor but for the detection of Cu(II) ions are furo[2,3-*d*]pyrimidines-2,4[1*H*,3*H*]-diones **85**. The chromophores consist of fused furopyrimidine and are generated *via* multicomponent strategy with a chromophore approach (Supplementary Material S29) (Kumar et al., 2017). The MW assisted three-component reaction of 1,3-dimethylbarbituric acid, benzaldehyde and respective isocyanides corresponding isocyanides proceeds *via* Knoevenagel condensation, [4 + 1] cycloaddition, and a 1,3-H shift to form two furopyrimidinones in excellent yields.

The furo[2,3-*d*]pyrimidines-2,4[1*H*,3*H*]-diones **85** display dual channel sensing of Cu(II) ions in solution and in the membrane phase. The electroanalytic study exhibits an ion selective electrode response toward Cu(II) ion in membrane phase. In the presence of Cu(II) ions, the absorption maximum decreases at the longest wavelength of the four absorption maxima (*λ*_*max,abs*_ = 210, 271, 325 and 375 nm) and results in a decolorization of the initially yellow solution. The emission spectra are not affected by Cu(II) ions. The proposed complex based on the ^1^H NMR study shows that the furan oxygen atom and the alkylamino NH group are directly involved in the coordination of Cu(II) ions (Supplementary Material S30). The coordination can be cleaved by sequestering agents such as EDTA. The chemical sensor is recoverable and efficiently reused several times.

Another MCR to synthesize pyrimidine chromophores is the three-component reaction of 5-amino-1*H*-pyrazole-4-carbonitrile, *p*-substituted benzoylacetonitriles and triethylorthoesters (Supplementary Material S31) (Ghotekar et al., 2009). The cyclocondensation forms pyrazolo[1,5-*a*]pyrimidines **86** and **87** *via* two methods in good yields in the sense of the chromophore concept. If the reaction proceeds in toluene with triethylamine as a catalyst 7-(4-aryl) pyrazolo[1,5-*a*]pyrimidine-3,6-dicarbonitriles **86** are obtained, whereas catalysis with HCl in ethanol furnishes 7-amino-6-(4-aroyl)pyrazolo[1,5-*a*]pyrimidine-3-carbonitriles **87**.

All pyrazolo[1,5-*a*]pyrimidines display intense fluorescence. The compounds **86** show absorption maxima between 267 and 296 nm and emission maxima between 304 and 332 nm. The amino group at the C_7_-position of compound **87** enhances the optical properties (*λ*_*max,abs*_ = 336–360 nm, *λ*_*max,em*_ = 393–414 nm) in comparison to the aryl group of compounds **86**. If dimedone is replaced by benzoylacetonitriles in the synthesis, pyrazolo[1,5-*a*]quinazolines can be prepared through cyclocondensation in refluxing toluene.

Quinoxaline can be considered as a benzo[*b*]fused pyrazine, which leads to a strongly electron-deficient π-system, enabling its application in chromophores for DSSCs (Chang et al., 2011; Pei et al., 2012; Wu and Zhu, 2013). A variety of quinoxaline derivatives show inherent fluorescence with significant solvatochromic shifts in the emission bands (Woody et al., 2011; Schaffroth et al., 2013). Quinoxaline derivatives can be accessed *via* various MCRs, which can proceed in a domino to sequential or consecutive manner. The activation-alkynylation-cyclocondensation (AACC) and glyoxylation-alkynylation-cyclocondensation (GACC) sequences provide a simple and elegant way to synthesize various quinoxalines *via* MCR (Gers-Panther et al., 2017; Merkt et al., 2018; Biesen and Müller, 2021).

**SCHEME 18:**
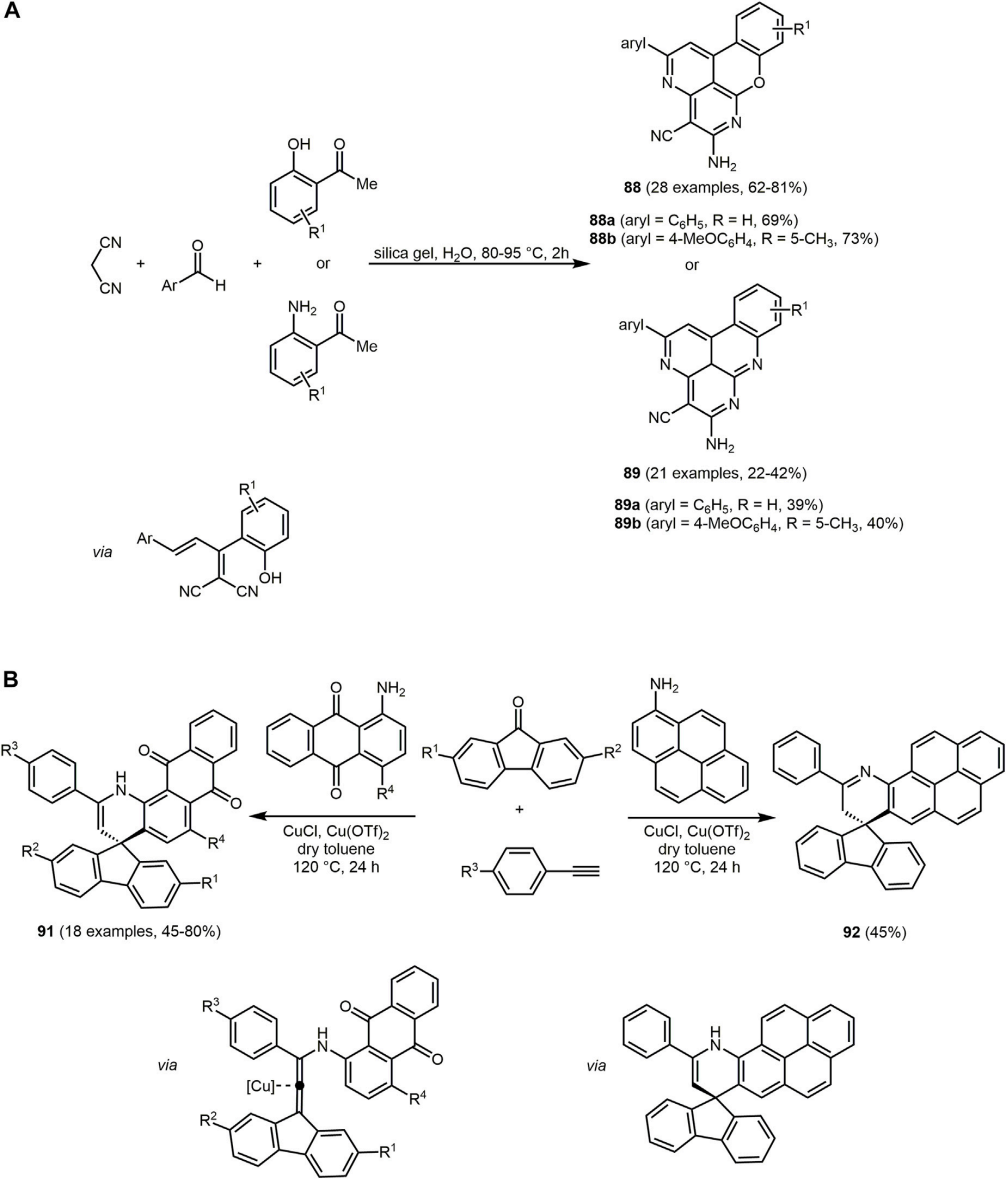
**(A)** Silica gel-catalyzed one-pot syntheses of 5-amino-2-aryl-3*H*-chromeno[4,3,2-*de*][1,6]naphthyridine-4-carbonitriles **88** and 5-amino-2-aryl-3*H*-quinolino[4,3,2-*de*][1,6]naphthyridine-4-carbonitriles **89** (Wu et al., 2010). **(B)** Synthesis of spirofluorenonaphthoquinolines **91** and **92** through MCR of 9-fluorenones, aryl alkynes, and aminoanthraquinones 1-aminopyrene (Meerakrishna et al., 2016).

Moreover, the condensation of aromatic aldehyde and substituted 2-hydroxyacetophenone or 2-aminoacetophenone and malononitrile gives rise to 5-amino-2-aryl-3*H*-chromeno[4,3,2-*d,e*][1,6]naphthyridine-4-carbonitriles **88** and 5-amino-2-aryl-3*H*-quinolino[4,3,2-*d,e*][1,6]naphthyridine-4-carbonitriles **89** in the sense of a pseudo five-component MCR (Scheme 18A) (Wu et al., 2010). The process proceeds in aqueous medium and is catalyzed by silica gel, an easily available, inexpensive, and non-toxic substance. First, a chalcone is formed by aldol condensation of the aldehyde and the 2-hydroxyacetophenone, which then reacts with malononitrile. Subsequent cyclization and condensation with another malononitrile take place to give the desired products upon aromatization.

The tetracycles **88** and **89** show strong fluorescence in EtOH upon irradiation with UV light (*λ*_*exc*_ = 360 nm), also with high fluorescence quantum yields. Moreover, emissions in the visible, allow applications as fluorescent probes, OLEDs, or luminescent materials. In fact, naphthyridine derivatives have already been used as luminescent materials for molecular recognition due to their planar rigid structure (Peng et al., 2005; Lu et al., 2006).

Nitroquinolines **90** can be produced by reacting *p*-nitroaniline, benzaldehydes and phenyl acetylene in presence of the Lewis acid niobium pentachloride under mild conditions (Supplementary Material S32) (dos Santos et al., 2017).

The optical properties of the compounds **90** can be altered by substituents on the benzaldehyde. The two absorption bands appear in a range of 250–280 nm and 325–393 nm, ascribed to the π-π*-transition and *n*-π*-transition. Electron-donating substituents cause a bathochromic shift in the absorption spectra. The nitroquinolines can be reduced with hydrazine monohydrate in the presence of 10% Pd/C to give aminoquinolines, which exhibit high quantum yields up to 0.83.

If ketones are used instead of aldehydes in the three-component reaction in the sense of the scaffold approach, access to substance libraries of highly conjugated, fluorescent spirofluorenonaphthoquinolines **91** are formed (Scheme 18B) (Meerakrishna et al., 2016). Using aryl alkynes with electron-donating groups in the copper-catalyzed reaction of ketones, alkynes, and amines (KA^2^ coupling) lead to higher yields due to the increased nucleophilicity of the copper acetylide. Mechanistically, the reaction proceeds by Cu(I)-catalyzed nucleophilic addition of phenylacetylide to 9-fluorenone to give a propargyl alcohol, which reacts with aminoanthraquinone forming an amino allene that undergoes intramolecular arylation and subsequent aromatization. Structurally different spirofluorenophenalenoquinoline derivatives **92** are obtained by using 1-aminopyrene as the amino component in an analogous reaction.

The absorption spectra of chromophores **91** are generally characterized by an absorption maximum between 537 and 614 nm, whereas dye **92** shows two absorption maxima 391 and 412 nm. The compounds **91** exhibit orange-red fluorescence, while pyrenospirofluoreno-naphthoquinoline **92** shows emission in the deep blue region with the highest Stokes shifts (Δ $\tilde{\nu }$ = 3,738 cm^−1^). The emissions of **91** are accompanied with low quantum yields presumably caused by the ketone bridge (Usta et al., 2009; Jacques et al., 2014).

The isatin-based spiro compounds **93** are formed *via* Knoevenagel condensation between isatins and malononitrile, followed by a Michael-type addition of *meta*-phenylenediamine, subsequent intramolecular cyclization and tautomerization (Supplementary Material S33) (Kundu et al., 2013).

All isatin-based spiro compounds **93** show similar absorption and emission spectra. A red shift of the emission maxima can be observed by increasing the solvent polarity and fluorescence quantum yields and lifetimes are also affected by the polarity of the solvent. The solvatochromism is characteristic for quinolines (Mataga and Tsuno, 1957; Atkinson and Speakman, 1971). Furthermore, the isatin-based spiro compounds **93** can be used as an ON-OFF switch chemosensor for Cu(II) ions.

**SCHEME 19:**
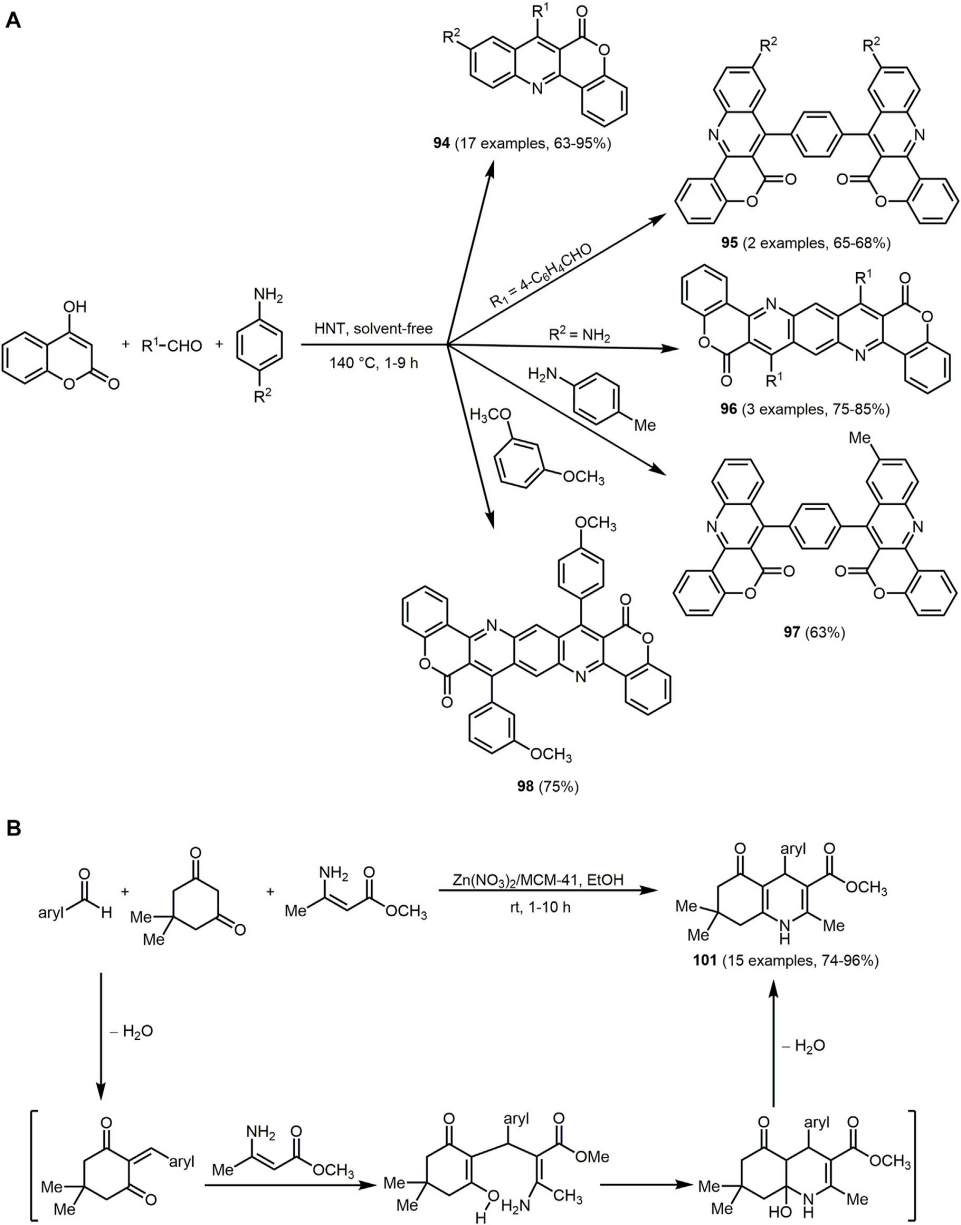
**(A)** Green one-pot three-component synthesis of chromeno[4,3-*b*]quinolin-6-ones **94**–**98** (Ataee-Kachouei et al., 2019). **(B)** Three-component synthesis of hexahydroquinolin-5-ones **101** (Oskuie et al., 2020).

A three-component reaction of 4-hydroxycoumarin, aldehydes and primary amines produces a huge substance library of quinoline chromophores **94** (Scheme 19A) (Ataee-Kachouei et al., 2019). The synthesis of chromeno[4,3-*b*]quinolin-6-ones and their symmetrical and unsymmetrical dyes **95**–**98** is catalyzed by naturally occurring halloysite nanotube (HNT) with the general formula of (Al_2_(OH)_4_Si_2_O_5_⋅2 H_2_O) (Rawtani and Agrawal, 2012). The solvent-free conditions, excellent yields, short reaction times and the low-cost, environmentally friendly and reusable catalyst characterize this reaction procedure as a green approach. The fluorophores **94**–**98** fluoresce blue and green and emission maxima can be detected in a range from 400 to 535 nm.

Coumarin-fused dihydroquinolines **99** are available in excellent yields (82%–94%) in the sense of the chromophore approach from 4-hydroxycoumarin, aldehydes and aromatic amines with bismuth triflate as a catalyst in water under microwave irradiation (Supplementary Material S34) (Khan et al., 2014). Mechanistically, Schiff base formation followed by condensation with 4-hydroxycoumarin generates the intermediate that reacts by carbonyl condensation with the aromatic amine followed by 6 π -electrocyclization and isomerization to furnish the dyes **99**. Some of the dyes **99** are highly fluorescent and exhibit high quantum yields of up to 0.65.

The catalyst-free three-component reaction of various α,β-unsaturated aldehydes, 2-hydroxy-1,4-naphthoquinone and several 5-aminopyrazoles afford pentacyclic pyran-fused pyrazolobenzo[*h*]quinoline derivatives **100**, which incorporate four bioactive components such as pyran, pyridine, pyrazole and α-naphthol (Supplementary Material S35) (Yadav et al., 2022). It is suggested that the α,β-unsaturated aldehyde reacts with 2-hydroxy-1,4-naphthoquinone *via* a Knoevenagel reaction. This is followed by the C-nucleophilic attack of 5-aminopyrazole *via* the C_4_ position. The two subsequent cyclizations yield the desired product **100**.

Nearly all of the synthesized derivatives fluoresce strongly under UV light. The quantum yields are in the range of 0.24–0.42 with Stokes shift of about 200 nm. The emission maxima are found at 490–514 nm.

The Zn/MCM-41-catalyzed (ZnNO_3_-impregnated MCM-41) unsymmetrical Hantzsch three-component condensation of various aryl aldehydes, dimedone and methyl-3-aminocrotonate proceeds under mild conditions and gives rise to the formation of hexahydroquinolin-5-ones **101** (Scheme 19B) (Oskuie et al., 2020).

The absorption maxima of the hexahydroquinolin-5-ones **101** can be detected at around 365 nm and emission at around 450 nm. The compounds **101** exhibit high Stokes shifts ranging from 4,800 to 5,800 cm^−1^ and moderate quantum yields up to 0.28. In addition, some of the synthesized hexahydroquinolines show anticancer activities.

The benzoanellated quinolinones **102** can be synthesized *via* one-pot reaction of 6-methoxy-1,2,3,4-tetrahydro-naphthalin-1-one, trimethoxybenzaldehyde, ethyl cyanoacetate and ammonium acetate under microwave irradiation (Supplementary Material S36) (Khan and Asiri, 2015; Zayed and Kumar, 2017). The 2-oxo-quinoline-3-carbonitrile derivative **102b** also shows antibacterial properties.

The intramolecular charge transfer band in the absorption spectrum and emission solvatochromicity of the donor-acceptor quinoline dyes **102** account for polar excited states. The quinoline-based chromophores **102a** and **102b** achieve quantum yields of up to 0.40 and 0.59, respectively.

The sequential three-component synthesis of homophthalonitrile, *o*-hydroxybenzaldehyde, and a nucleophile gives rise to chromenoisoquinolines (Festa et al., 2017). If (aza)indole acts as the nucleophile, 12-(1*H*-indol-3-yl)12*H*-chromeno[2,3-*c*]isoquinolin-5-amines **103** are obtained in the sense of the chromophore approach (Supplementary Material S37) (Festa et al., 2019).

The UV/Vis spectra of indol-3-yl substituted dyes **103** show similar absorption and emission maxima at around 354 and 415 nm, respectively. The highest fluorescence quantum yields (*Φ*_*F*_ = 0.42–0.70) are obtained in polar and protic solvents. In addition, the compounds **103** undergo reversible fluorescence quenching under acidic conditions. These optical properties result from the localized electron density of the frontier orbitals on the isoquinolinamine moiety and the equal energy gaps of the associated frontier molecular orbitals.

Phenothiazines are electron-rich heterocyclic organic π-systems and, thus, they are interesting donors in dyes. As a consequence of their substitution pattern they tunable with respect to reversible oxidation potentials and luminescence (Sun et al., 2005; Sasaki et al., 2007; Miura et al., 2010). In general, donor-acceptor systems are utilized in molecular electronics and photonics (Armstrong et al., 2001; Lai et al., 2001; Richter, 2004; Dini, 2005; Kulkarni et al., 2005), in OLEDs (Kraft et al., 1998; Mitschke and Bäuerle, 2000; Kulkarni et al., 2004; Walker et al., 2011), as well as in photovoltaic devices (Hoppe and Sariciftci, 2004; Walker et al., 2011; Lin et al., 2012b). Remarkably, *N*-substituted phenothiazines are used for the construction of AIE compounds (Zhang et al., 2015b; Okazaki et al., 2017; Chen et al., 2019; Gong et al., 2019; Ekbote et al., 2020) resulting from their non-planar “butterfly” shaped conformation, which is folded along the *S,N* axis (Bell et al., 1968; Klein et al., 1985). Access to phenothaizines in the sense of an MCR can be achieved, for example, *via* an Ugi reaction (Bay et al., 2013; Bay et al., 2014; Bay and Müller, 2014).

**SCHEME 20:**
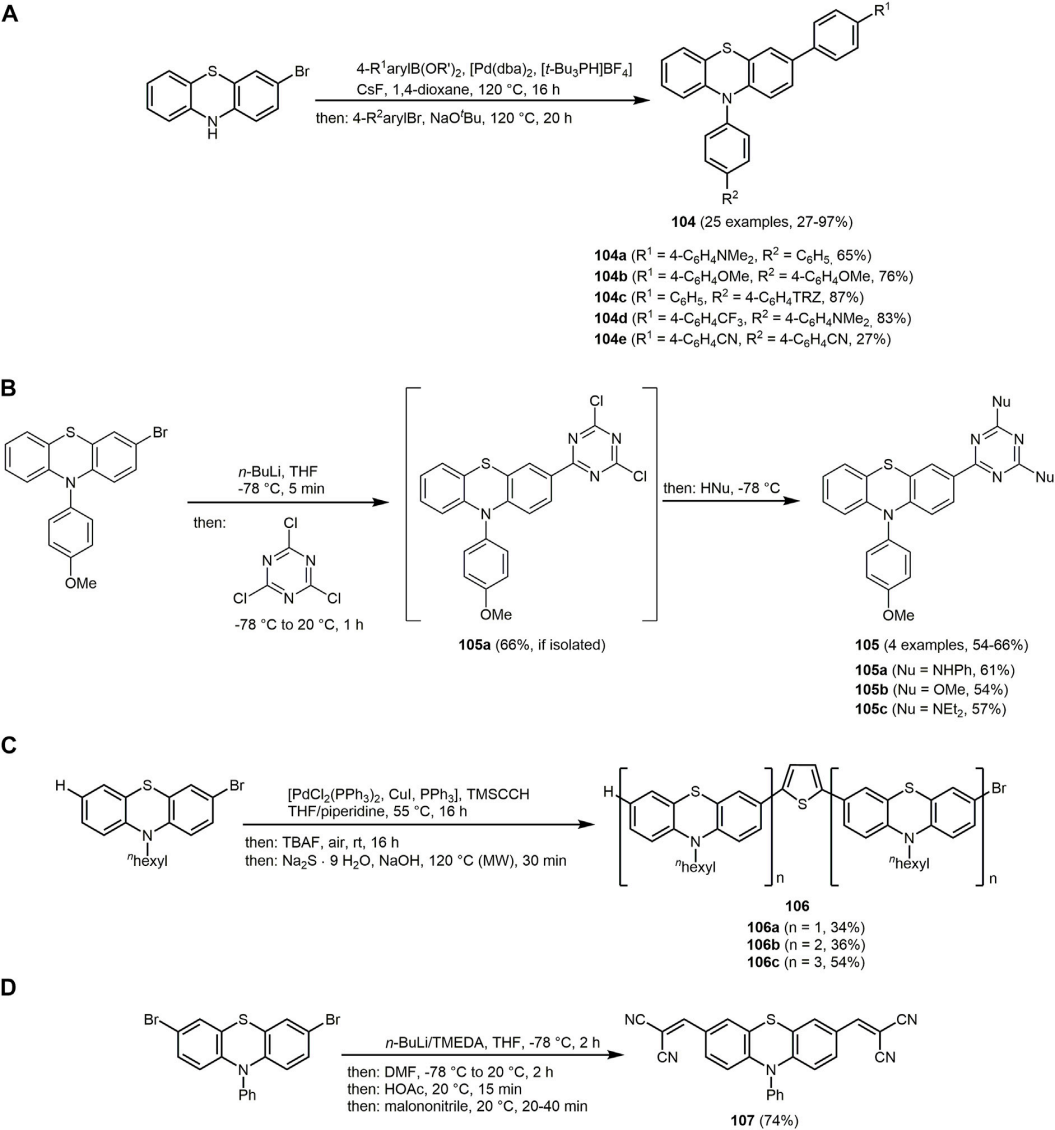
**(A)** Sequentially Pd-catalyzed arylation-amination consecutive three-component synthesis of 3,10-diaryl 10*H*-phenothiazines **104** (TRZ = 2,4-diphenyl-1,3,5-triazine) (Mayer et al., 2020; Mayer and Müller, 2021). **(B)** One-pot sequence to synthesize disubstituted phenothiazine-triazine dyes **105** (Kloeters et al., 2022). **(C)** Pseudo five-component Sonogashira–Glaser cyclization synthesis of thienyl-bridged oligophenothiazines **106** (Urselmann et al., 2016). **(D)** One-pot LiForK synthesis of a 3,7-diacceptor substituted phenothiazine **107** (May and Müller, 2020).

Just recently, 3,10-diaryl phenothiazines **104** were generated *via* a sequentially Pd-catalyzed three-component arylation-amination sequence in a one-pot fashion (Scheme 20A) (Mayer et al., 2020; Mayer and Müller, 2021). The consecutive Suzuki arylation-Buchwald-Hartwig amination sequence was applied to obtain 25 different examples of 3,10-diarylphenothiazines **104** in moderate to very good yields by varying arylboronic acids or esters as well as the aryl bromides with electron-donating and electron-withdrawing substituents in the *para*-position.

The electron properties of the 3,10-diaryl 10*H*-phenothiazines derivatives **104** can be fine-tuned by varying electronic substituents on the phenothiazine that extend the π-electron conjugation and the fundamental conformational change from *intra*- to *extra*-configuration. Using an elaborated structure-property relationship 3D diagram based on the correlation between the first oxidation potential ( $E_{0}^{0/+1}$ ) and the Hammett parameters $\sigma_{p}^{+}$ (R^1^) and $\sigma_{p}$ (R^2^), a prediction or tailoring of the oxidation potential is possible. The modification of the electronic substituents allows to tune the emission color over the entire spectral range from blue to red.

Placing the heterocycle 1,3,5-triazine instead of the aryl radical in 3-position, also a considerable color spectrum can be covered by modulating the acceptor strength of the triazine moiety in the solid state under excitation under UV light (λ_exc_ = 365 nm). The triazine building blocks have immense importance due to their extraordinary biological activities, especially in chemical medicine (Verma et al., 2020). Synthetically, after brominelithium exchange of the starting component 3-bromo-*N*-anisyl-phenothiazine with BuLi and introduction of the trichloro-1,3,5-triazine core by subsequent double nucleophilic substitution, the phenothiazine-triazine chromophores **105** can be obtained (Scheme 20B) (Kloeters et al., 2022).

In general, compounds **105** show high fluorescence quantum yields in solution and in the solid state. By tuning the substitution pattern on the triazine, photophysical properties such as thermally activated delayed fluorescence (TADF) and white light emission can occure. Thus, solvatochromism studies of electron-deficient substituted triazine chromophores reveal strong charge transfer character and a small singlet-triplet energy gap, hence these derivatives in particular **105a** identify as TADF candidates. While the electron rich triazine dyes show a reversible shift of the spectral emission upon protonation. Furthermore, a white light emission can be observed for derivatives **105d** (Supplementary Material S38).

The phenothiazine moiety has also been implemented in linear (Sailer et al., 2008) or cyclic (Memminger et al., 2008) oligomer topologies as well as diphenothiazinyl dumbbells linked by heterocycles (Franz et al., 2009; Hauck et al., 2010; Jahnke et al., 2014). The synthesis of symmetrical thienyl-bridged oligophenothiazine dumbbells **106** is feasible *via* a consecutive pseudo five-component Sonogashira-Glaser cyclization sequence (Scheme 20C) (Urselmann et al., 2016).

The absorption spectra of compounds **106** exhibit four absorption bands. Three appear at shorter wavelengths, which can be assigned to the phenothiazinyl units, and the longest wavelength maximum can be attributed to the central 2,5-di (hetero)aryl-substituted thiophene moiety. The molar decadic extinction coefficient increases with the number of phenothiazinyl units. The thienyl-bridged oligophenothiazines **106** emit in a wavelength range from 506 to 521 nm with large Stokes shifts between 4,800 and 5,600 cm^−1^, which are characteristic for oligophenothiazines (Sailer et al., 2008). Fluorescence quantum yields of chromophores **106** range from 0.15 to 0.18. A cathodic shift of oxidation potentials is observed for the series with increasing number of phenothiazinyl electrophore units. A consistently reversible oxidation range can be demonstrated for compound **106c**. Molecular modelling reveals lowest energy conformers that exhibit a sigmoidal and helical structure. TD-DFT calculations and even semiempirical ZINDO (Zerner’s intermediate neglect of differential overlap) calculations confirm the trends of the absorption bands with the longest wavelengths. Thus, the charge transfer can be largely assigned between the electrophore moieties from the neighboring phenothiazinyl moieties to the central thienyl unit.

Diacceptor substituted phenothiazine **107** can be accessed *via* a lithium formylation-Knoevenagel condensation (LiForK) sequence (May and Müller, 2020). The consecutive pseudo five-component reaction initiated by bromine-lithium exchange forms an acceptor-donor-acceptor conjugate (Scheme 20D) (May and Müller, 2020).

*Via* the same reaction sequence, the heterocyclic topologically analoguous 2,6-diacceptor-substituted dithieno[1,4]thiazines **108** and **109** can also be prepared (Supplementary Material S39). Further *syn*- and *anti-anti* dithieno[1,4]thiazine isomers with diacceptor (**110a** and **111a**) and bisdonor (**110b** and **111b**) substitution pattern can be synthesized *via* the pseudo three-component reaction of *N*-phenyl dithieno[1,4]thiazine and 4-bromobenzonitrile or 4-iodoanisole *via* dilithiation-lithium-zinc exchange-Negishi coupling in yields ranging from 20% to 83%.

The interactions between the substituents in the dithieno[1,4]thiazines (**108** and **109**) is stronger than in the corresponding phenothiazine **107** since dithieno[1,4]thiazines are generally higher polarizable. Dithieno[1,4]thiazines **108** and **109** are characterized by cathodically shifted oxidation potentials and red-shifted, more intense absorption bands compared to the corresponding phenothiazines **107**. The analysis of the structure-property relationships points out that the photophysical and electrochemical properties as well as the electronic structure are significantly determined by the thiophene anellation mode of the products **108** and **109**. Thus, strong acceptors in *syn-syn*-dithieno[1,4]thiazines **108** and **110a** possess a rather folded structure as well as weak fluorescence (*Φ*_*F*_ = 0.01). In contrast, equally substituted *anti-anti* isomers **109** and **111a** show an almost planar ground state geometry and very intense near-infrared fluorescence (*Φ*_*F*_ = 0.52). In principle these red light or NIR emitters can be promising for potential application in biomedical imaging (Hong et al., 2017) or OLED-devices (Qu et al., 2006).

## 5 (Hetero)Arene

**SCHEME 21:**
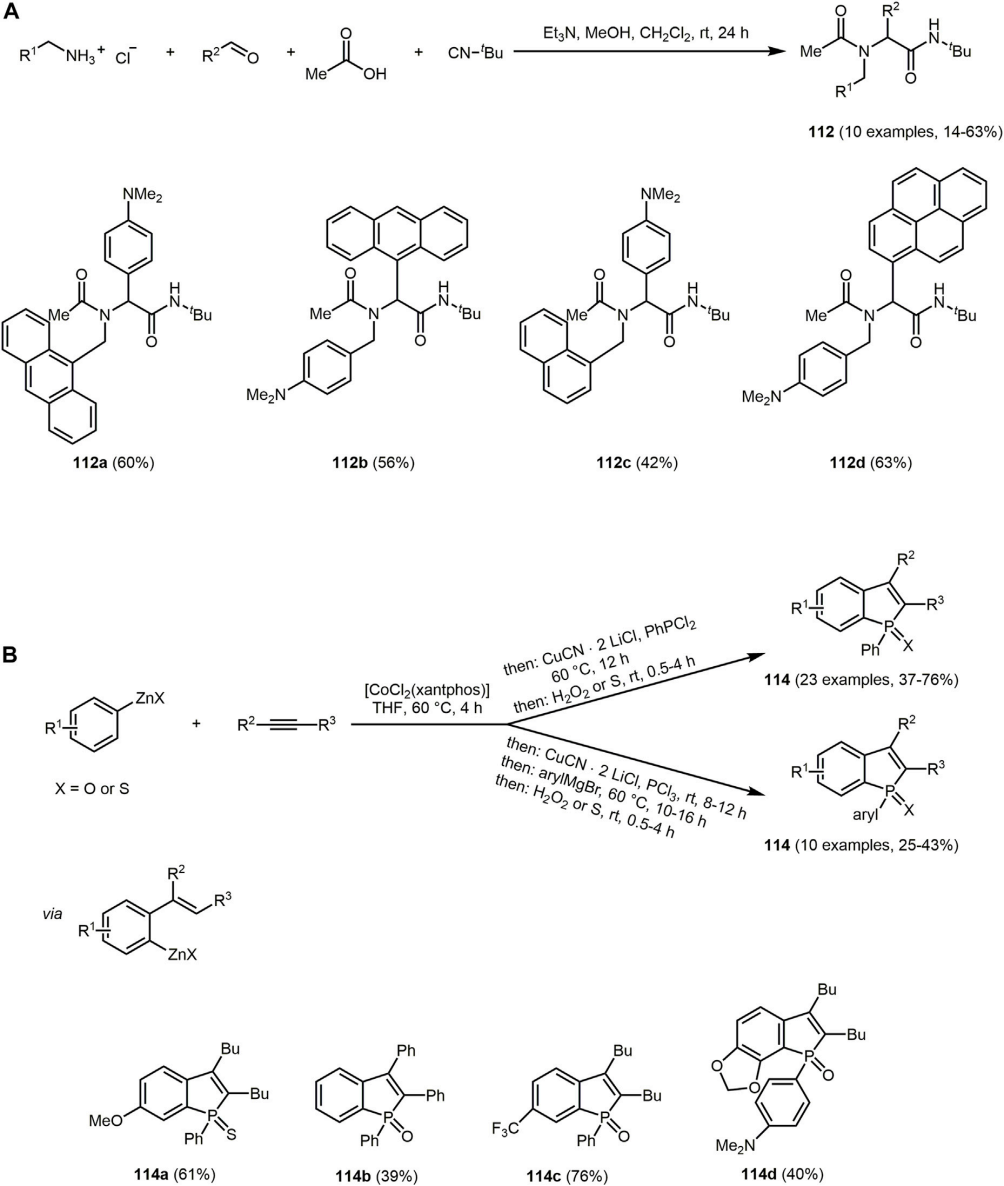
**(A)** One-pot Ugi 4CR synthesis of donor-acceptor dyads **112** and selected examples (Ochs et al., 2019). **(B)** One-pot synthesis of functionalized benzo[*b*]phosphole derivatives **114** and selected fluorophores (Wu et al., 2014).

Exciplexes (excited complexes) as well as excimers (excited dimers) are emitting charge transfer complexes, which are formed by excitation of one of the constituent chromophores, which collides with a second chromophore that is in the electronic ground state (Balzani, 2001; Balzani and Venturi, 2003). The Ugi four-component reaction provides an access to unimolecular exciplex emitting dyads **112** consisting of an *N,N*-dimethylaniline moiety as a donor and anthracene, naphthalene or pyrene as acceptor chromophores in the sense of the scaffold approach (Scheme 21A) (Ochs et al., 2019).

The chromophores of the donor-acceptor dyads **112** are electronically decoupled in the ground state and electronically coupled in the excited state. This results in exciplex emissions due to the polar nature of the excited state. The formation of the exciplex depends on partial electron transfer with matching redox potentials and on spatial proximity of the donor and acceptor units. Furthermore, the *N,N*-dimethylaniline-acceptor chromophores **112** are capable of photoinduced intramolecular electron transfer (PIET) and exhibit emission solvatochromism with red-shifted emission upon increasing solvent polarity (Supplementary Material S40). Based on TD-DFT calculations, the qualitative assignment of the experimental absorption and emission bands is possible.

1,4-Diarylbuta-1,3-diene derivatives are applied in liquid crystals, illuminants, and non-linear optical materials (Bartkowiak et al., 2001; Davis et al., 2003; Davis et al., 2004; Denmark and Tymonko, 2005; Abraham et al., 2006; Davis et al., 2008; Das et al., 2010). In addition, some MCRs are known for the synthesis of these chromophores (Zhang and Larock, 2003; Shibata et al., 2005; Horiguchi et al., 2008). A palladium-catalyzed three-component reaction of aryl iodides, diarylacetylenes, and cinnamic acids furnishes 1,4-diarylbuta-1,3-dienes **113** in the sense of the chromophore concept (Supplementary Material S41) (Yamashita et al., 2011). The sequence is initiated by oxidative addition of aryliodide with *in situ* generated Pd (0) species followed by alkyne insertion and ligand exchange with cinnamic acid giving a vinylpalladium carboxylate intermediate. Subsequent decarboxylation and reductive elimination give the products. 1,4-Diarylbuta-1,3-dienes **113** show solid state emission with maxima in a range from 440 to 540 nm.

Structurally diverse benzo[*b*]phospholes **114** are synthesized *via* facile regiocontrolled one-pot sequential coupling of an arylzinc reagent, an alkyne, dichlorophenylphosphane (or phosphorus trichloride and a Grignard reagent), and an oxidant (hydrogen peroxide or sulfur) (Scheme 21B) (Wu et al., 2014). For this MCR two common approaches with similar initiation steps can be applied. Cobalt-catalyzed migratory arylzincation forms the intermediate that reacts after transmetalation to an organic copper species with PhPCl_2_ followed by oxidation with hydrogen peroxide or sulfur powder. Alternatively, the formation of the benzo[*b*]phosphole oxides or benzo[*b*]phosphole sulfides **114** proceeds by reaction of the copper species with PCl_3_, followed by addition of Grignard reagents and final oxidation.

Most benzo[*b*]phosphole derivatives **114**, especially benzo[*b*]phosphole oxides, are fluorescent in solution. Electron-donating amino groups as substituents in the R^1^ position led to a significant redshift. In general, the longest wavelength absorption maxima appear between 317 and 394 nm. The emission maxima are located between 385 and 484 nm with fluorescence quantum yields of up to 0.93. In general, benzo[*b*]phospholes possess interesting optoelectronic properties and find application in organic electronic devices (Tsuji et al., 2009; Tsuji et al., 2010).

## 6 Azo chromophores

Azo dyes have a long history among synthetic dyes and are used in a wide range of applications, including in cosmetic, textile and paper industries (Şener et al., 2006; Benkhaya et al., 2017; Benkhaya et al., 2020). Azo dyes are characterized by one or more azo bridges as integral chromophore. The common preparation method of azo dyes is the diazotization of an aromatic primary amine followed by coupling with one or more electron-rich π-nucleophiles (Gürses et al., 2016). Due to the great importance and application of this dye class, several green syntheses of this class of compounds have been explored (Safari and Zarnegar, 2015; Nikpassand and Pirdelzendeh, 2016; Nikpassand et al., 2018; Nikpassand, 2020). For instance, the microwave assisted three-component reaction of arylazopyrazoles, benzaldehydes and dimedone proceed *via* Mannich cyclization and condensation to form pyrazoloquinazolinone azo dyes **115** (Supplementary Material S42) (Elgemeie et al., 2015). The absorption maxima of azo dyes **115** appear between 380 and 498 nm. In the presence of a nitro aryl substituent on the azo part, a bathochromic shift of the absorption maxima can be observed.

**SCHEME 22:**
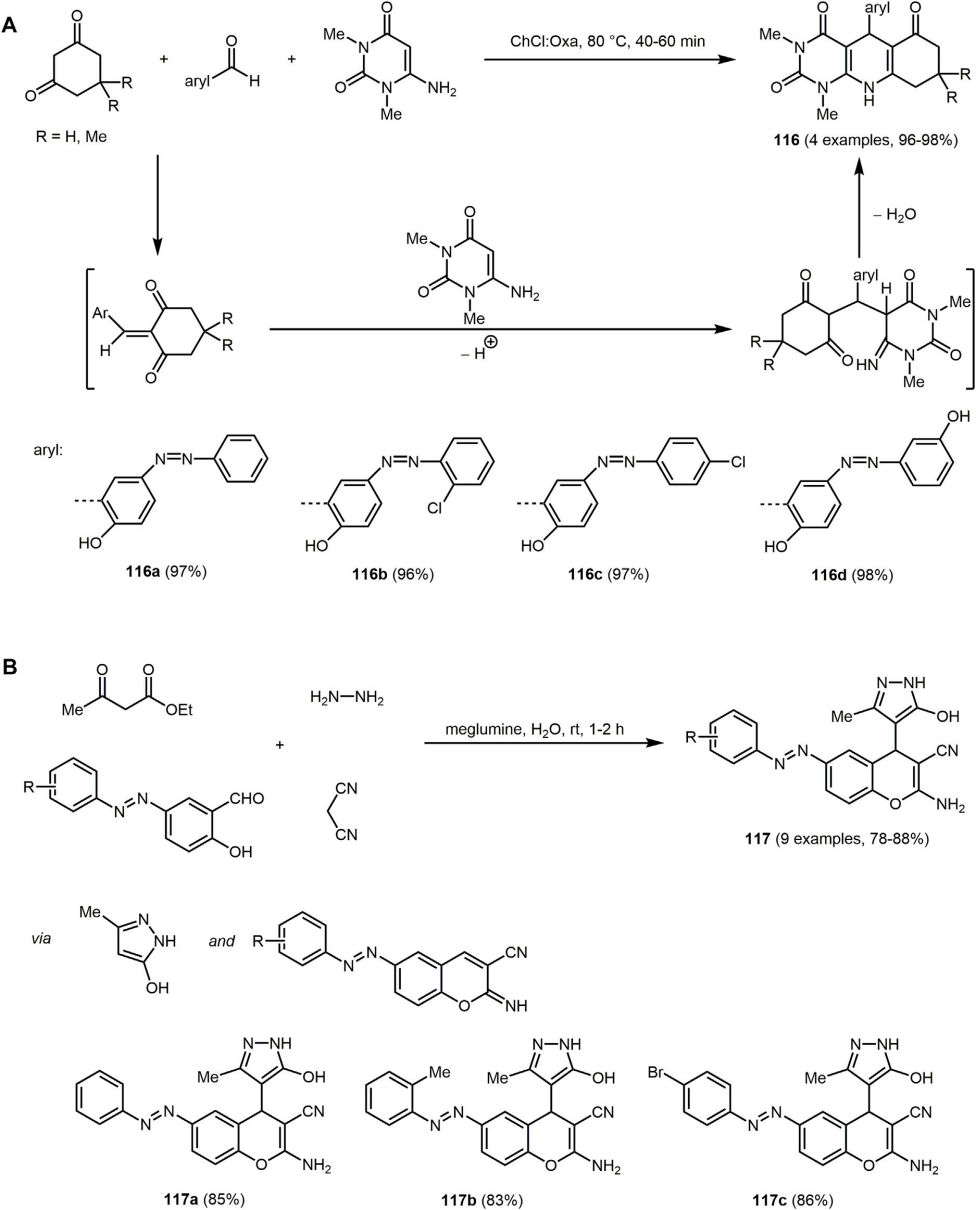
**(A)** Hantzsch synthesis of pyrimido[4,5-*b*]quinolines **116** and the four obtained derivates (Gholami et al., 2020). **(B)** Meglumine catalyzed one-pot synthesis of fluorescent 2-amino-4-pyrazolyl-6-aryldiazenyl-4*H*-chromene-3-carbonitriles **117** and selected derivates (Korade et al., 2021).

Azo pyrimido[4,5-*b*]quinoline derivatives **116** are prepared *via* an unsymmetrical Hantzsch synthesis with dimedone or 1,3-cyclohexadione, azo aldehydes, and 6-amino-1,3-dimethyluracil in the presence of choline chloride/oxalic acid (ChCl/Oxa) as a green solvent and recyclable substance catalyst (Scheme 22A) (Gholami et al., 2020). The UV/Vis spectra exhibit two absorption bands. The longest wavelength absorptions are found between 352 and 362 nm.

An energy efficient four-component synthesis starting from ethyl acetoacetate, hydrazine hydrate, azo salicylaldehydes, and malononitrile forms in the presence of meglumine (*N*-methyl-*D*-glucamine) as an organocatalyst fluorescent 2-amino-4-pyrazolyl-6-aryldiazenyl-4*H*-chromene-3-carbonitriles **117** in good to excellent yields (Scheme 22B) (Korade et al., 2021)

Absorption maxima of the dyes are found in the range of 290–294 nm and can be ascribed to the n–π* transitions. In contrast to many azo compounds the dyes **117** fluoresce with large Stokes shifts (Δ $\tilde{\nu }$ ≈ 17,100 cm^−1^) and emission maxima lie in a narrow range between 582 and 586 nm.

## 7 Miscellaneous

### 7.1 Metalcomplex dyes

Organoboron complexes have been known for quite some time (Michaelis, 1894; Michaelis and Richter, 1901), but only in the 21st century their fluorescent properties were recognized as favorable for application in OLEDs (Entwistle and Marder, 2002; Entwistle and Marder, 2004; Jäkle, 2010; Rao and Wang, 2011). For example, boron Schiff bases have indeed been employed in OLEDs (Vidyasagar et al., 2019) and as non-linear optical chromophores (Reyes et al., 2002; Lamère et al., 2006; Muñoz et al., 2008; Jiménez-Pérez et al., 2015a; Jiménez-Pérez et al., 2015b). In addition, they are used in bioimaging (António et al., 2019; Ibarra-Rodríguez et al., 2019; Russo et al., 2020).

**SCHEME 23:**
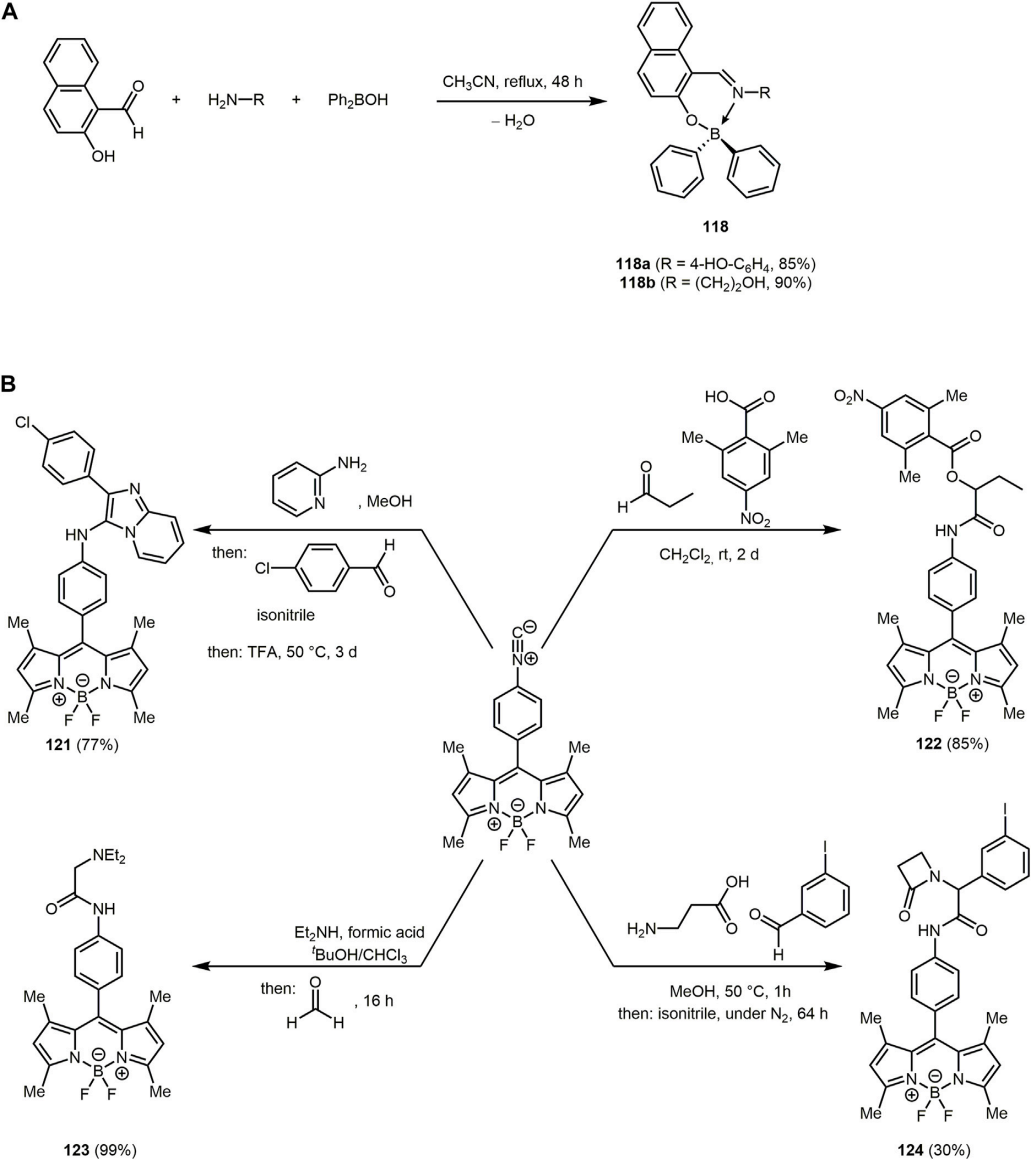
**(A)** One-pot synthesis of boron Schiff base dyes **118** (Corona-Lopez et al., 2021). **(B)** Derivatization of an isonitrile functionalized BODIPY dye *via* various MCR to synthesize compounds **121**–**124** (Vazquez-Romero et al., 2013).

Recently, a multicomponent synthesis of two boron Schiff bases **118a** and **118b** through the condensation reaction of 2-hydroxynaphthaldehyde with the corresponding amines and *in situ* generated diphenylborinic acid has been reported (Scheme 23A) (Corona-Lopez et al., 2021).

The longest wavelength absorption maxima can be detected between 396 and 404 nm. The emission maxima lie between 474 and 525 nm with large Stokes shifts, but low quantum yields. In addition, boron complexes **118a** and **118b** can be classified as semiconductors based on the determined optical band gaps *E*_*g*_ between 2.57 and 2.78 eV for the n- π * electronic transitions of the substituent groups.

Further tetracoordinated boron atoms bearing Schiff bases **119** can be synthesized *via* a three-component condensation reaction starting from damnacanthal as a biogenic component in a remarkably rapid manner (Supplementary Material S43) (Garcia-Lopez et al., 2022).

The organoboron esters **119** are fluorescent and exhibit emission maxima at around 400 nm. Notably, the oscillator strength of the electronic transition can be influenced by the position of the substituent. Thus, the derivative with the nitro group substituent in 4-position displays a larger extinction coefficient. Similar to the organoboron complexes **118**, the chromophores **119** also show low quantum yields around 0.01 and can also be classified as semiconductors due to the optical band gaps (*E*_*g*_ ≈ 252 eV). As a result of the damnacanthal utilized as the starting material, **119** possess biological activity and in particular **119b** showed cytotoxicity activity against MDA-MB-231 breast cancer cells.

A fast and efficient approach to chromophores applicable also in biological fields such as boron hydrazone dyes **120**, can be achieved by microwave-assisted domino multicomponent condensation reaction of diverse aryl aldehydes, benzoylhydrazide, or 4-nitrobenzoylhidrazine, and diphenyl boronic acid (Supplementary Material S44) (Molina-Paredes et al., 2019).

The UV/Vis spectra of boron complexes **120** exhibit either one or two absorption maxima and longest wavelength absorption bands can be detected in a range from 368 to 448 nm with molar extinction coefficients ε between 13,000 and 55,000 M^−1^ cm^−1^. The emission maxima appear between 420 and 520 nm with moderate Stokes shifts (Δ $\tilde{\nu }$ = 2,980–3,400 cm^−1^) for most complexes, except for dye **120c** (Δ $\tilde{\nu }$ = 7,330 cm^−1^). Although the organoboron dyes have low fluorescence quantum yields, they can be used to stain silk fibroin. Therefore, they can be employed in the development of scaffolds for tissue engineering due to their confirmed non-toxicity.

Further organoboron complexes have low cytotoxicity are employed in the medical diagnostics, especially as p*H* indicators and cell markers (Baruah et al., 2006; Yang et al., 2013; Liu et al., 2015; Yang et al., 2016). In particular, 4,4-difluoro-4-bora-3a,4a-diaza-s-indacene (BODIPY) scaffold, which exhibits excellent photophysical properties, are commonly encountered in fluorescent probes (Loudet and Burgess, 2007; Ulrich et al., 2008; Boens et al., 2012; Kolemen and Akkaya, 2018). Emission and absorption typically below 600 nm as well as small Stokes shifts and high quantum yields are characteristic of fluorophores containing a BODIPY core (Gürses et al., 2016). Using a series of multicomponent reactions starting from isonitrile functionalized BODIPY dye allows for derivatization of this framework (Scheme 23B) (Vazquez-Romero et al., 2013). The isonitrile-BODIPY scaffold was previously synthesized starting from BODIPY aniline and subsequently functionalized by Groebcke-Bienaymé-Blackburn (**121**), Passerini (**122**), and Ugi reaction (**123** and **124**).

The emission and absorption of the fluorescent BODIPY dyes **123** lie in the typical range of BODIPY fluorophores and exhibit quantum yields of up to 0.61. The dyes **121** can be utilized as a fluorescent probe for *in vivo* imaging of phagocytosing macrophages.

Moreover, by varying the functional group of the BODIPY dye further substance libraries of BODIPY complexes can be obtained by MCR. The Passerini reaction of formyl-containing BODIPY derivatives with benzoic acid and *t*-butyl isocyanide yields highly substituted BODIPY dyes **125** and **126** (Supplementary Material S45) (Ramirez-Ornelas et al., 2016). The formyl-containing BODIPY complex are prepared *via* Liebeskind−Srogl cross-coupling or Vilsmeier reaction starting from Biellmann BODIPYs (Goud et al., 2006).

The photophysical properties of the BODIPY dyes are not affected by the ligations to the *para* position of the 8-phenyl group or to 2-position directly on the BODIPY core, but rather are influenced by the free motion of the 8-aryl. For example, boronic complexes containing an unhindered 8-phenyl (**125a** and **126a**) show a low fluorescence response, due to free rotational motion of the ring. In contrast, high quantum yields (*Φ*_*F*_ = 0.82) are detected for aryls with methylation at the *ortho* positions (**126b**), as mesityl groups prevent the aryl from rotating freely due to a higher rotational barrier. In addition, these complexes can be used to stain blood cells with very intense and stable signals at a very low exposure time.

**SCHEME 24:**
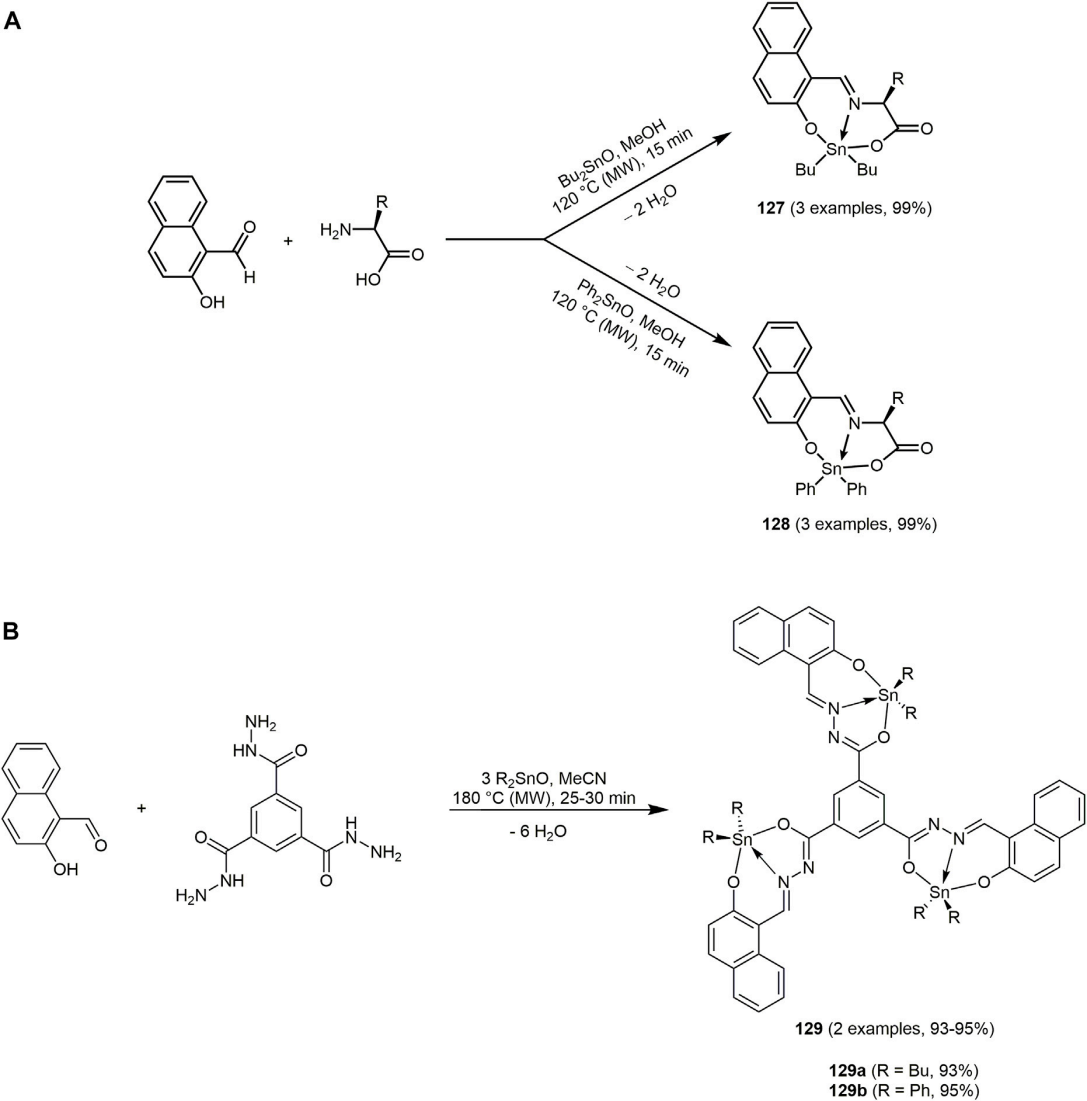
**(A)** One-pot condensation reaction to synthesized pentacoordinate and chiral organotin compounds **127** and **128** derived from amino acid based Schiff bases (Lara-Ceron et al., 2017). **(B)** Microwave-assisted pseudo seven-component condensation reaction to synthesize two fluorescent pentacoordinated organotin complexes **129** derived from Schiff bases with three central tin atoms (Canton-Diaz et al., 2021).

Luminescence in metal complexes typically results from metal-to-ligand charge transfer in the excited state (Fredericks et al., 1979; Striplin and Crosby, 1994). Therefore, their photophysical properties are characterized by high Stokes shifts and long luminescence lifetimes, which are especially essential in the bioanalytical field (Ma et al., 2014; Albada and Metzler-Nolte, 2016). For instance, organotin compounds derived from Schiff base complexes can be applied as analytical luminescent chemosensors for the identification of metals (Vinayak and Nayek, 2019). In addition, since these complexes are capable of staining silk fibroin, they can potentially be used as scaffolds for tissue engineering (Lara-Ceron et al., 2017). Organotin complexes with *n*-butyl (**127**) and phenyl residues (**128**) can be prepared by microwave-assisted three-component condensation reaction of 2-hydroxy-1-naphthaldehyde, *L*-amino acids, and diorganotin oxides (Scheme 24A). Intrinsically fluorescent amino acids, such as tryptophan, tyrosine, and phenylalanine can be thereby implemented.

The UV/Vis and fluorescence spectra of compounds **127** and **128** display similar maxima. Two absorption maxima are observed at 420 nm, which can be assigned to n-π* transitions of the carboxylate and imine groups, and at 335 nm, which can be assigned to the π-π* intraligand charge transfer within the naphthyl segment. The absorption maximum of the π-π* transition also differs from the free ligands (*λ*_*max,abs*_ = 303 nm). The bathochromic shift of the bands for the tin complexes can be attributed to azomethine-N→Sn coordination. The emission can be detected at a maximum of 465 nm fluorescence quantum yields ranging from 0.08 to 0.21.

By replacing the amine with just one primary amine group by a component with three reactive moieties, such as benzene-1,3,5-tricarbohydrazide, organotin compounds with a C_3_-symmetric Schiff base **129** with excellent yields can be generated (Scheme 24B) (Canton-Diaz et al., 2021).

The photophysical properties of the two organotin complexes with three central tin atoms are similar to those with one central tin atom in which [*N*-(2-oxido-1-naphthaldehyde)-4-hydroxybenzyhydrazidate] was employed (Jiménez-Pérez et al., 2015b). However, the extinction coefficient of **129a** exhibits a larger value (ε = 68,400 M^−1^ cm^−1^) and thus a larger oscillator strength. Theoretical calculations attribute the S_0_→S_1_ excitation to natural transition orbitals. The tin atoms do not interact electronically, as both organotin complexes show quantum yields in chloroform of approximately 0.52, with lifetimes of about 3 ns. Both complexes show green emission in solution.

Sn(IV)-porphyrins have an affinity for oxygen donor ligands (Arnold and Blok, 2004). Therefore, these complexes are suitable for the construction of axially coordination bound multiporphyrin arrays (Redman et al., 2001; Prodi et al., 2002; Scandola et al., 2006; Shetti et al., 2012). In a one-step procedure *meso*-pyridyl Sn(IV)-porphyrin, *meso*-hydroxyphenyl-21,23-dithiaporphyrin, and Ru(II)-porphyrin react to give Sn(IV)-porphyrin-based oligomers **130** with yields ranging from 60% to 70% (Supplementary Material S46) (Dvivedi et al., 2014). Here, the Ru(II)-porphyrins are coordinated as peripheral ligands to the *meso*-pyridyl group(s) of the Sn(IV)-porphyrin.

The similar overlapping absorption bands of all three constituent monomers with slight shifts in their absorption maxima and the weak interaction of the porphyrin units in the oligomers observable in the electrochemical study indicate that the various porphyrin units of compound **130** act as decoupled supramolecular arrays. However, the steady state fluorescence study shows emission quenching in presence of the Ru(II)-porphyrin units, presumably caused by strong spin-orbit coupling.

### 7.2 Peptoidic dyes

**SCHEME 25:**
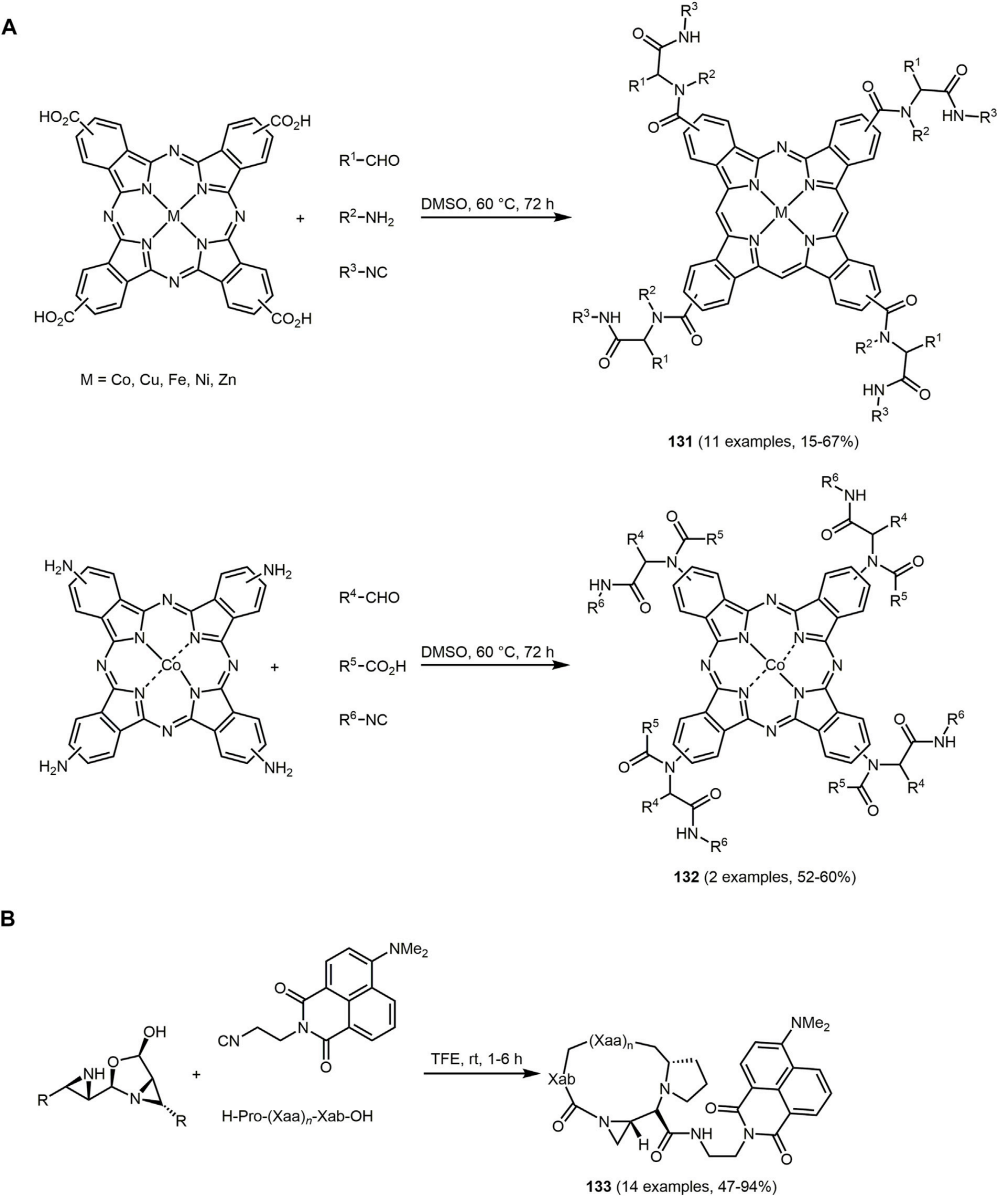
**(A)** One-pot catalyst-free Ugi 4CR for the synthesis carboxamide-modified-metallophthalocyanines **131** and **132** (Afshari et al., 2019). **(B)** One-pot synthesis of fluorophore decorated macrocyclic peptides **133** (Rotstein et al., 2011).

Phthalocyanines are structurally related to porphyrins. This macrocyclic chromophore can be employed in Ugi 4CR in a scaffold approach to access sidechain modified metallophthalocyanines **131** and **132** (Scheme 25A) (Afshari et al., 2019). The electrochemical and optical properties as well as the solubility of phthalocyanines can be adjusted by rational tuning of the metal center as well as modification and functionalization of substrates. In the case of dye **131**, where cobalt, copper, iron, nickel, and zinc ions are implemented as metal centers, the phthalocyanine acts as the carboxylic acid component in the Ugi reaction. For the synthesis of the cobalt complex **132**, tetra-amino cobalt (II) phthalocyanine acts as the starting material, underling the substrate diversity of the synthetic route.

The phthalocyanines containing carboxamide moieties **131** and **132** are characterized by high stability, diminished aggregation and increased of monomerization, resulting in good solubilty of the metallophthalocyanines in common solvents, especially in water.

Peptides perform a wide range of physiological functions in the human organism and possess biochemical properties that are valuable for drug development (Sánchez and Vázquez, 2017). Therefore, synthetic peptides have found numerous applications in the cellular arena and tagging them with fluorophores makes them suitable fluorescent imaging agents and activity-based probes (Blum et al., 2005; Loving and Imperiali, 2009; Sainlos et al., 2009; Baumes et al., 2010; Kwan et al., 2011). However, peptides have limited stability against proteolysis *in vivo* (Henninot et al., 2018; Lee et al., 2019). Cyclic peptides possess higher *in vivo* proteolytic stability and better cellular permeability (Matsuzaki et al., 1997; Gudmundsson et al., 1999; Fletcher et al., 2008). A possible synthetic route to fluorophore decorated macrocyclic peptides **133** is offered by the multicomponent reaction of peptides, aziridine aldehydes and isocyanides bearing a solvatochromic fluorophore (Scheme 25B) (Rotstein et al., 2011). The fluorescent peptide macrocycles **133** are fluorescent and their maximum emission incorporated di- and tripeptides lies in the range from 495 to 500 nm.

## 8 Conclusion and outlook

MCRs are a valuable tool for synthesizing functional organic chromophores with unique photophysical and electrochemical features. Two strategies, the scaffold and the chromophore concepts, have to provide the targeted structures, where chromophores are either ligated to a scaffold (which also can constitute a new chromophore) or where the chromophore, mostly linear and cyclic conjugated systems, is formed in a chromogenic fashion. Besides aiming for new chromophores, arrays of established chromophores, or providing systems for establishing systematic structure property relationships, in recent years diversity-oriented syntheses relying on green approaches involving environmentally friendly solvents, catalysts and purification processes have become increasingly important and intellectually challenging in the field of MCRs. Tailormade functional π-systems accessed by MCR not only provide fluorophores with AIE effects for extensive application possibilities as luminescent materials in optoelectronics (OLED, OFET, DSSC, OPV), but also reach by analytics applications beyond to life science and biomedical engineering. Furthermore, newly explored effects, such as AIE (aggregation-induced emission) or TADF (thermally activated delayed fluorescence), demand suitable tunable chromophores. Therefore, existing and still undiscovered MCRs will also provide synthetic solutions for tackling new actual and future scientific problems in chromophore research, which has become an evergreen in chemical sciences.

## Author’s Note

Dedicated to the memory of Prof. Dr. Mikhail Krasavin (1975–2023).
