# Supplementary figures and images for: Multicomponent synthesis of chromophores – The one-pot approach to functional π-systems
Source: Front Chem. 2023 Mar 17;11:1124209. doi: 10.3389/fchem.2023.1124209 (PMC10065161; doi:10.3389/fchem.2023.1124209)

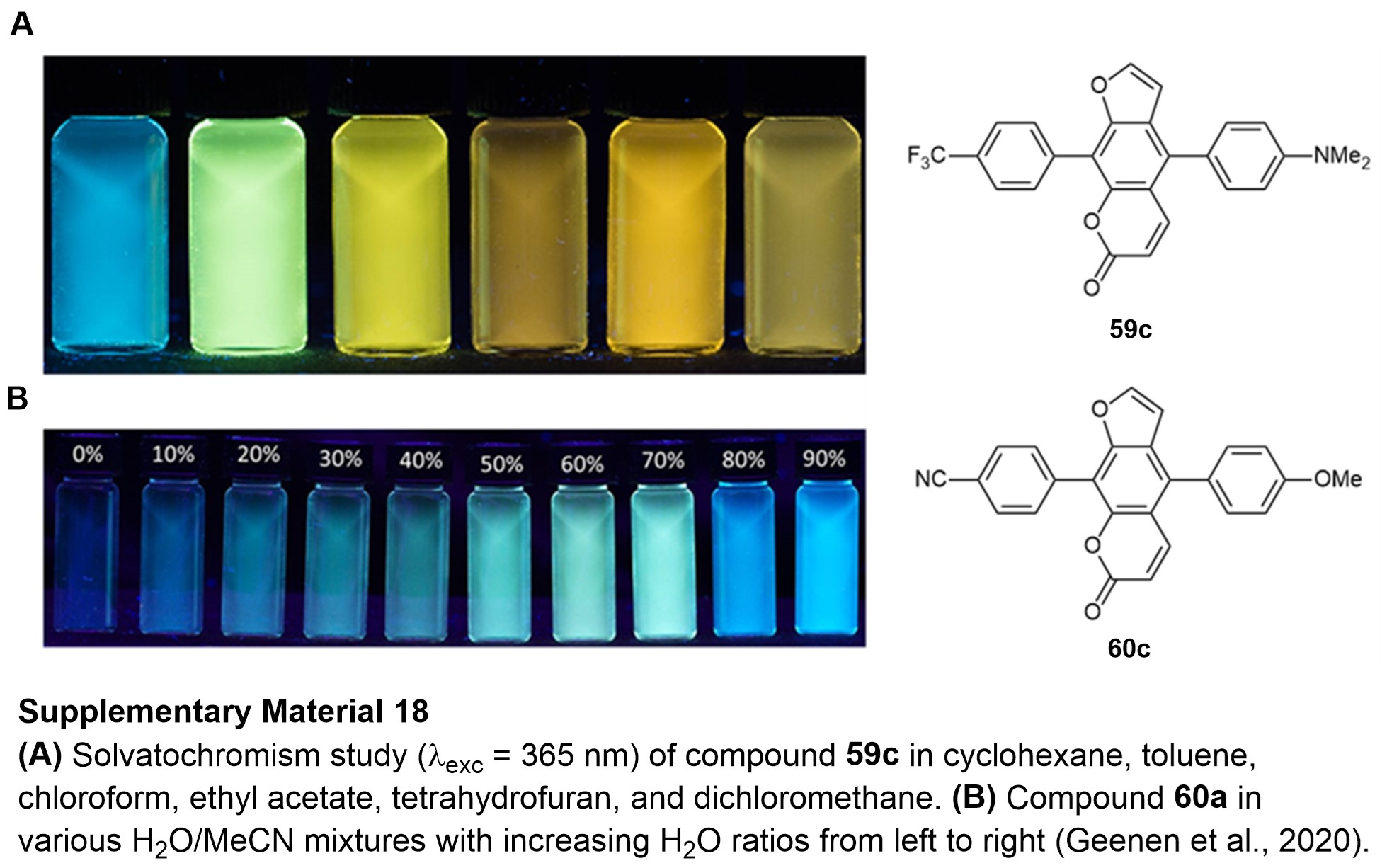

Supplement: Supplementary file 1 [file DataSheet1.zip › S18.jpg]

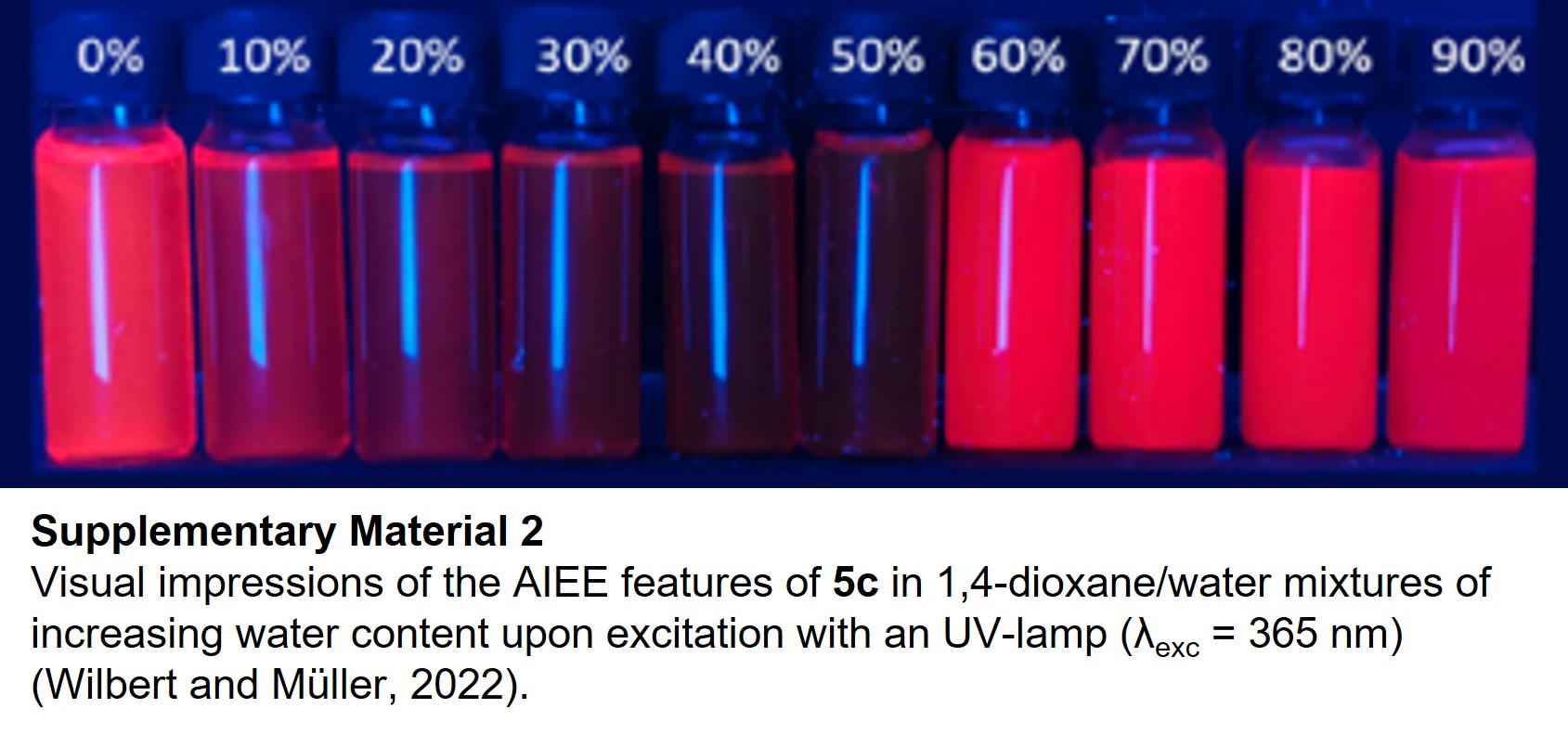

Supplement: Supplementary file 1 [file DataSheet1.zip › S2.jpg]

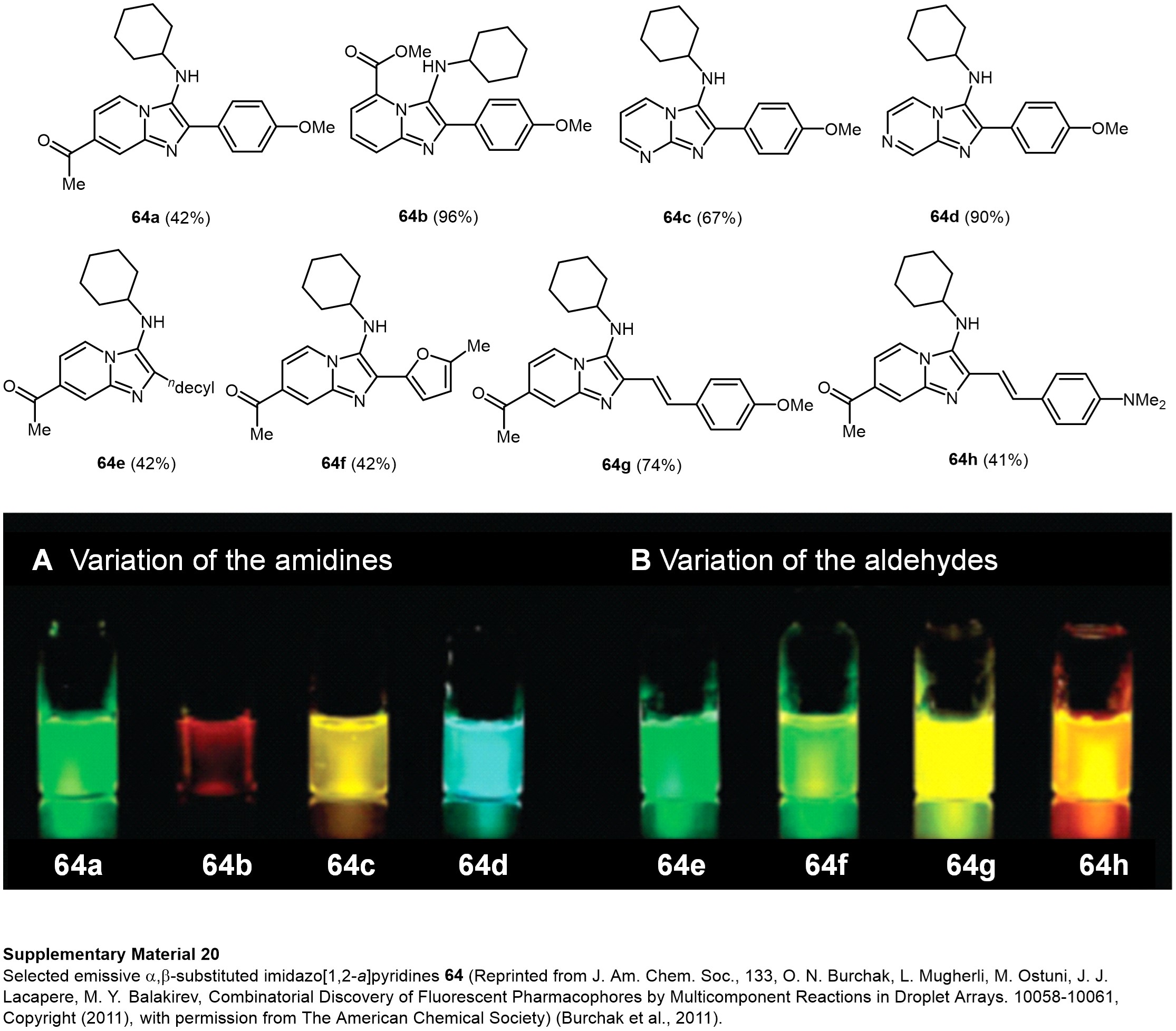

Supplement: Supplementary file 1 [file DataSheet1.zip › S20.jpg]

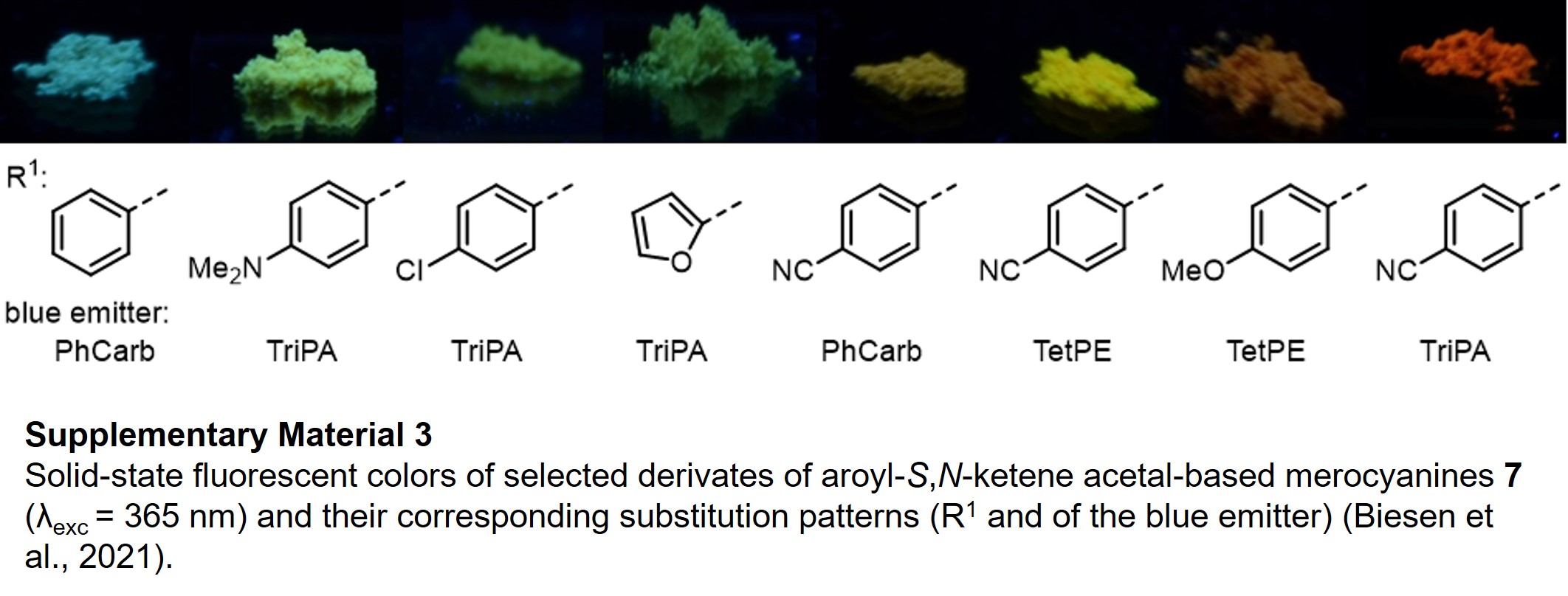

Supplement: Supplementary file 1 [file DataSheet1.zip › S3.jpg]

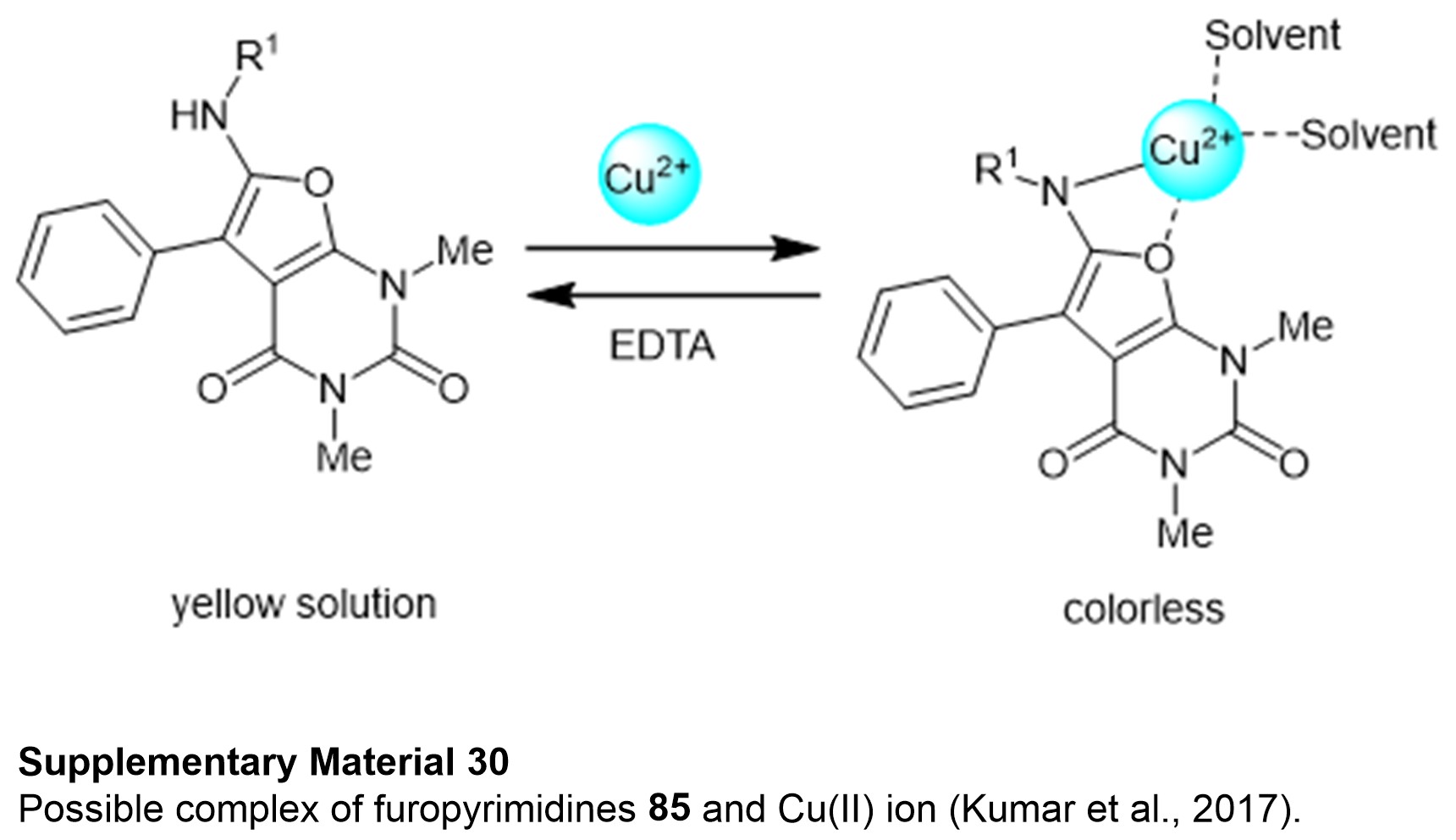

Supplement: Supplementary file 1 [file DataSheet1.zip › S30.jpg]

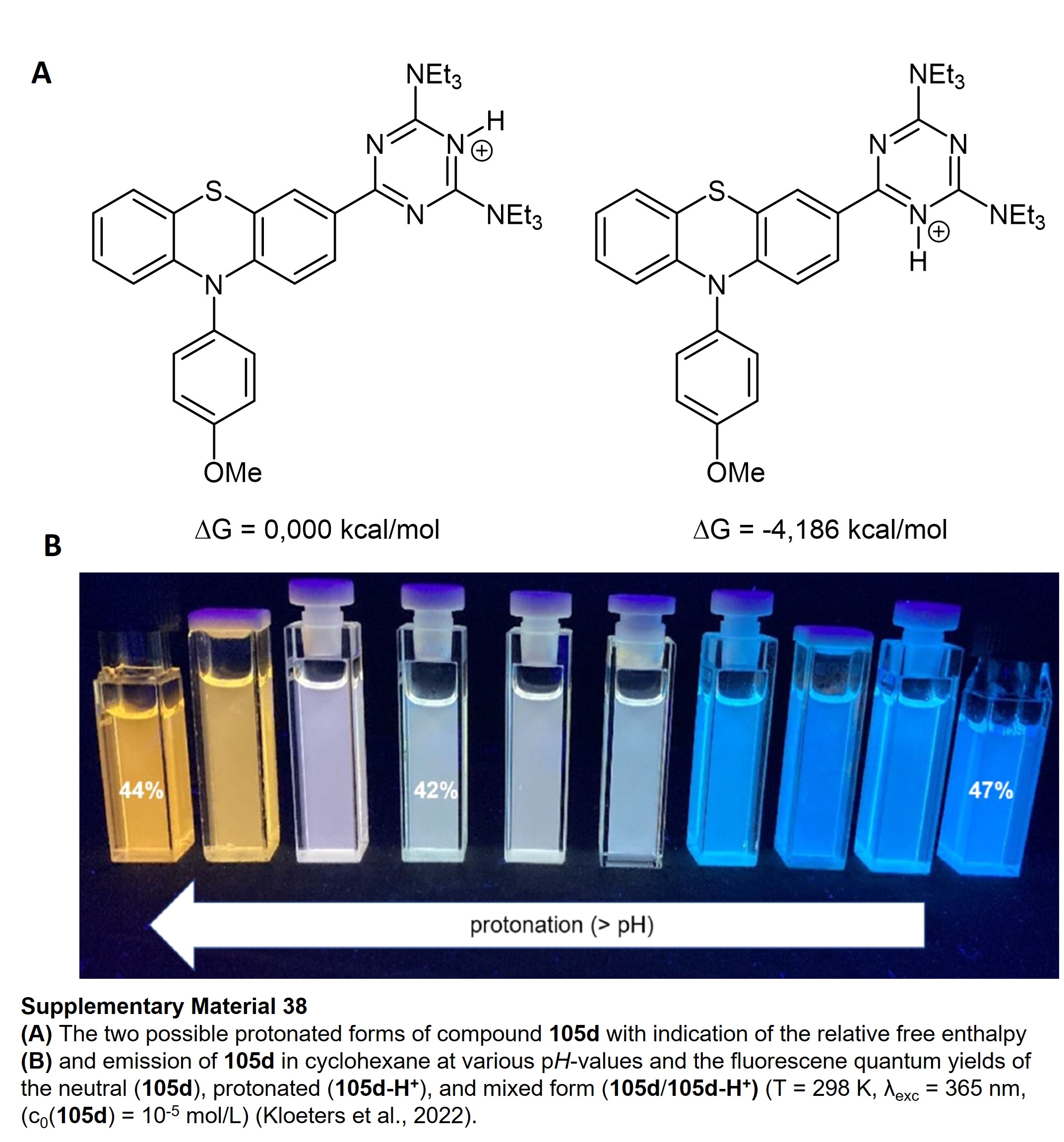

Supplement: Supplementary file 1 [file DataSheet1.zip › S38.jpg]

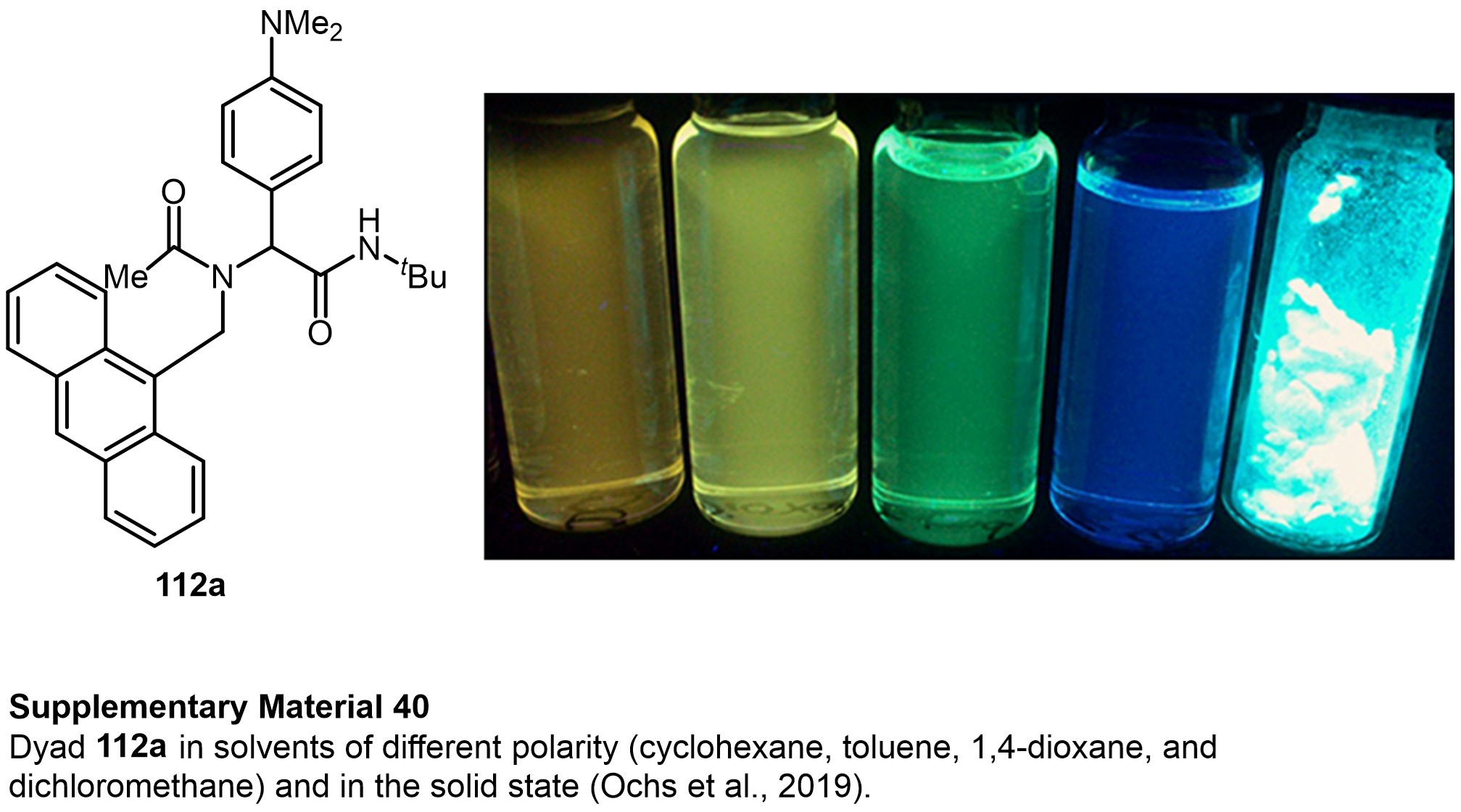

Supplement: Supplementary file 1 [file DataSheet1.zip › S40.jpg]

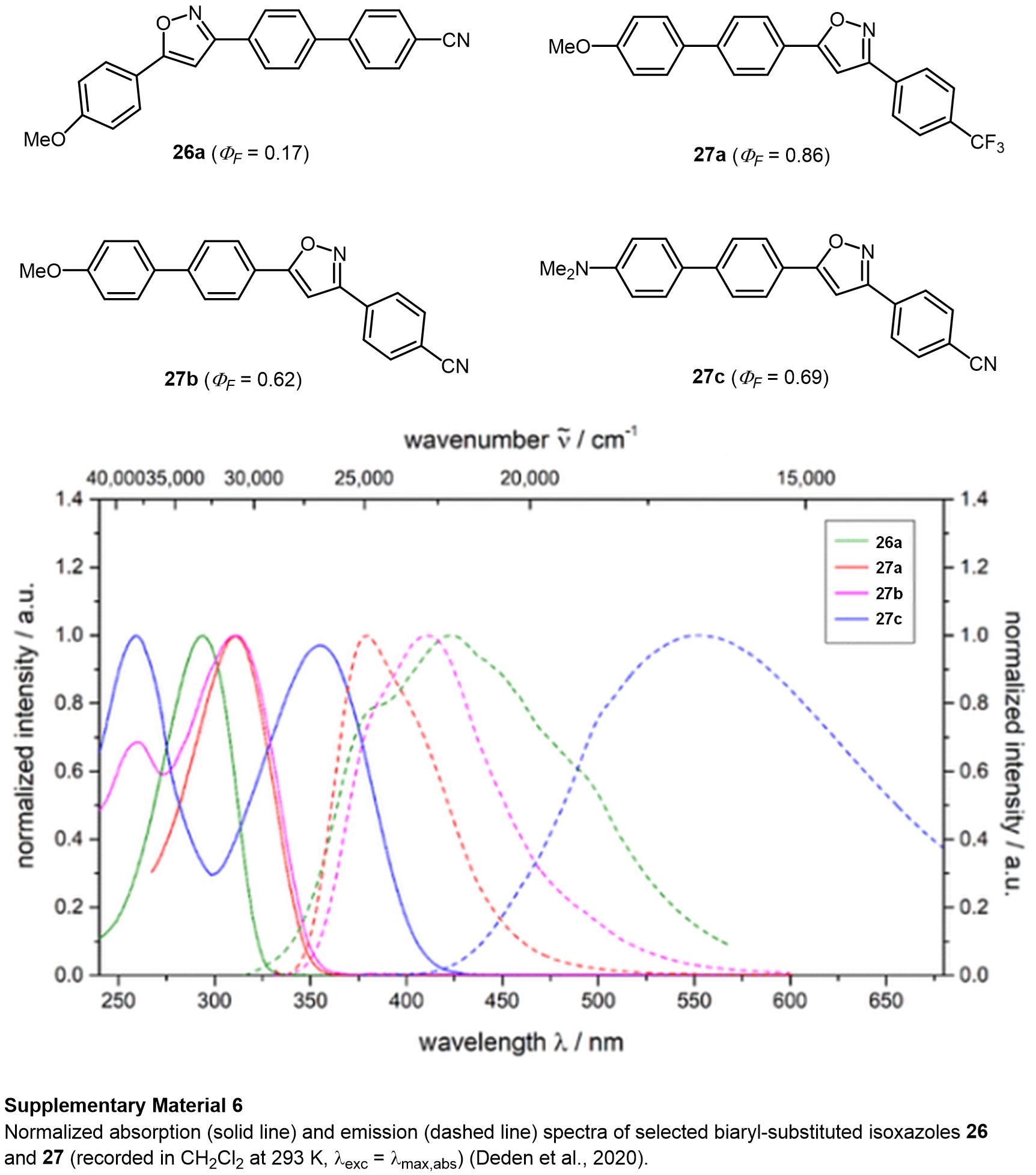

Supplement: Supplementary file 1 [file DataSheet1.zip › S6.jpg]

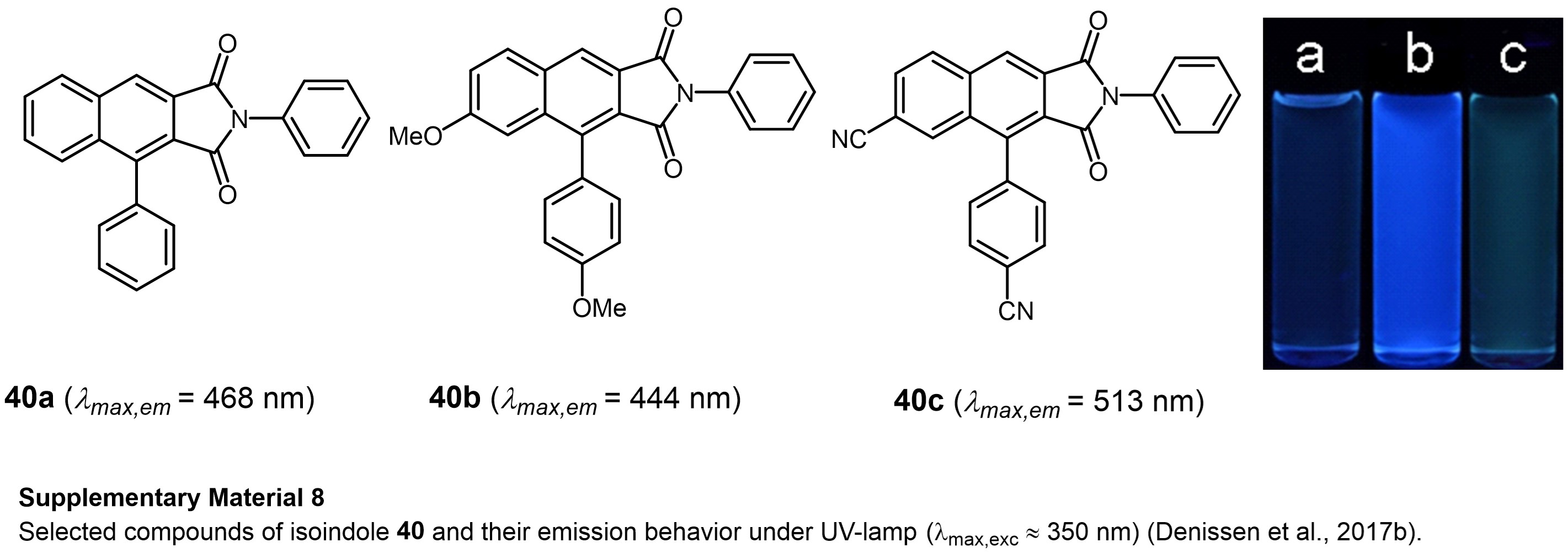

Supplement: Supplementary file 1 [file DataSheet1.zip › S8.jpg]
